# Blood pressure, hypertension and the risk of atrial fibrillation: a systematic review and meta-analysis of cohort studies

**DOI:** 10.1007/s10654-022-00914-0

**Published:** 2023-01-10

**Authors:** Dagfinn Aune, Yahya Mahamat-Saleh, Elsa Kobeissi, Tingting Feng, Alicia K. Heath, Imre Janszky

**Affiliations:** 1 Department of Epidemiology and Biostatistics, School of Public Health, Imperial College London, St. Mary’s Campus, Norfolk Place, Paddington, London, W2 1PG UK; 2 Department of Nutrition, Bjørknes University College, Oslo, Norway; 3 Department of Endocrinology, Morbid Obesity and Preventive Medicine, Oslo University Hospital, Oslo, Norway; 4 International Agency for Research on Cancer, Lyon, France; 5 Global Health Institute, American University of Beirut, Beirut, Lebanon; 6 Norwegian Registry for Vascular Surgery, Trondheim, Norway; 7 Department of Public Health and Nursing, Norwegian University of Science and Technology, Trondheim, Norway; 8 Department of Global Public Health, Karolinska Institutet, Stockholm, Sweden

**Keywords:** Blood pressure, Hypertension, Atrial fibrillation, Systematic review, Meta-analysis, Cohort studies

## Abstract

Elevated blood pressure and hypertension have been associated with increased risk of atrial fibrillation in a number of epidemiological studies, however, the strength of the association has differed between studies. We conducted a systematic review and meta-analysis of the association between blood pressure and hypertension and atrial fibrillation. PubMed and Embase databases were searched for studies of hypertension and blood pressure and atrial fibrillation up to June 6th 2022. Cohort studies reporting adjusted relative risk (RR) estimates and 95% confidence intervals (CIs) of atrial fibrillation associated with hypertension or blood pressure were included. A random effects model was used to estimate summary RRs. Sixty eight cohort studies were included in the meta-analysis. The summary RR was 1.50 (95% CI: 1.42–1.58, I^2^ = 98.1%, n = 56 studies) for people with hypertension compared to those without hypertension (1,080,611 cases, 30,539,230 participants), 1.18 (95% CI: 1.16–1.21, I^2^ = 65.9%, n = 37 studies) per 20 mmHg increase in systolic blood pressure (346,471 cases, 14,569,396 participants), and 1.07 (95% CI: 1.03–1.11, I^2^ = 91.5%, n = 22 studies) per 10 mmHg increase in diastolic blood pressure (332,867 cases, 14,354,980 participants). There was evidence of a nonlinear association between diastolic blood pressure and atrial fibrillation with a steeper increase in risk at lower levels of diastolic blood pressure, but for systolic blood pressure the association appeared to be linear. For both systolic and diastolic blood pressure, the risk increased even within the normal range of blood pressure and persons at the high end of systolic and diastolic blood pressure around 180/110 mmHg had a 1.8–2.3 fold higher risk of atrial fibrillation compared to those with a blood pressure of 90/60 mmHg. These results suggest that elevated blood pressure and hypertension increases the risk of atrial fibrillation and there is some increase in risk even within the normal range of systolic and diastolic blood pressure.

**Supplementary Information:**

The online version contains supplementary material available at 10.1007/s10654-022-00914-0.

## Introduction

Atrial fibrillation presents a considerable public health burden and is the most common type of arrhythmia affecting around 1–2% of the general population, increasing to around 10% of persons by 80 years of age [1]. Five million incident cases were diagnosed worldwide in 2010 [2] and the prevalence of atrial fibrillation has been estimated at 33 million in 2015 [3]. In the USA the prevalence of atrial fibrillation has been projected to increase from 2.3 million in 1996–1997 to 5.6 million by 2050 [4]. Patients with atrial fibrillation are at increased risk of a number of complications, most notably stroke, heart failure, dementia and all-cause mortality [5, 6]. Several risk factors for atrial fibrillation have been established including age, sex, diabetes, coronary heart disease, heart failure, smoking, alcohol, obesity, low physical activity and possibly high intensity physical activity [7–15].

Elevated blood pressure is the leading cause of death and disability-adjusted life-years (DALYs) globally with 10.4 million deaths and 218 million DALYs attributable to elevated systolic blood pressure in 2017 according to the Global Burden of Disease Study [16]. Although elevated blood pressure is an established risk factor for several cardiovascular diseases, data regarding blood pressure and risk of atrial fibrillation have to our knowledge not been summarized in a meta-analysis. A large number of cohort studies have investigated the association between hypertension and the risk of atrial fibrillation [7–9, 17–65], and most of these found an increased risk [7, 8, 17–22, 24–31, 33, 34, 36–49, 51–62, 64, 65], with few studies reporting no association [9, 23, 32, 35, 50, 63], however, the strength of the association has differed considerably between studies with reported relative risks reported ranging from 0.93 to 2.85 [7–9, 17–59]. In addition, several studies have investigated the association between systolic [8, 18, 20, 29, 33, 34, 39, 41, 42, 44, 46, 51, 55, 63, 65–80] or diastolic [8, 29, 33, 39, 41, 44, 46, 51, 55, 67–70, 72–74, 79, 81] blood pressure and risk of atrial fibrillation with most studies reporting increased risk for increasing systolic blood pressure [18, 20, 29, 34, 39, 41, 44, 46, 51, 55, 65–72, 74–80], while results have been more mixed for diastolic blood pressure with some showing an increased risk [39, 44, 51, 55, 67–69, 72, 79, 82] but other studies showing no association [8, 29, 33, 46, 70, 73, 74, 81], or even reduced risk [41, 77] with higher diastolic blood pressure.

Establishing whether hypertension and elevated blood pressure increases the risk of atrial fibrillation would be important from a preventive point of view as it is a risk factor that could be modified by diet, physical activity, weight control and pharmaceutical drugs [83]. In addition it would be useful to better characterize the strength and shape of the dose–response relationship between blood pressure and atrial fibrillation to clarify whether the association is dose-dependent or if there are threshold effects. We conducted a systematic review and meta-analysis of cohort studies on hypertension and blood pressure in relation to the risk of atrial fibrillation to clarify the strength and shape of the dose–response relationship, and to identify potential sources of heterogeneity in the results.

## Material and methods

### Search strategy and inclusion criteria

We searched Pubmed, and Embase databases up to June 9th 2022 for eligible studies. The search strategy is provided in the Supplementary Text. We followed standard criteria for conducting and reporting meta-analyses [84]. In addition, we searched the reference lists of the identified publications for further studies.

### Study selection

We included published retrospective and prospective cohort studies and nested case–control studies within cohorts that investigated the association between blood pressure or hypertension and the risk of atrial fibrillation (any type). Retrospective case–control studies were excluded because of their potential for recall bias and selection bias and cross-sectional studies were excluded because of difficulties in establishing cause and effect relationships. Estimates of the relative risk adjusted for at least one confounding factor had to be available with the 95% confidence intervals (CIs) in the publication. Conference abstracts, grey literature and non-English publications were not included. When multiple publications were available from the same study, the study with the largest number of cases was used in general. However, overlapping publications were used in specific subgroup analyses by sex or ethnicity, when the article used for the main analysis did not report such stratified analyses. Overlapping publications that reported risk estimates in three categories or more were also used for the nonlinear dose–response analyses (as the nonlinear analysis requires categorical data) if the article included in the main analysis only reported risk estimates on a continuous scale. A list of the excluded studies can be found in Supplementary Table 1. DA, YMS, EK and TF did the study selection in duplicate and any disagreements were resolved by discussion.

### Data extraction

The following data were extracted from each study: The first author’s last name, publication year, country where the study was conducted, study period, sample size, number of cases and participants, exposure (hypertension, systolic blood pressure, or diastolic blood pressure), subgroup (e.g. sex, race), relative risks (RRs) and 95% CIs for hypertension versus no hypertension or for increments in systolic or diastolic blood pressure and variables adjusted for in the analysis. DA did the data extraction and it was checked for accuracy by YMS.

### Statistical methods

We calculated summary RRs (95% CIs) of atrial fibrillation for participants with hypertension compared with participants without hypertension and for systolic and diastolic blood pressure using the random-effects model by DerSimonian and Laird [85] which takes into account both within and between study variation (heterogeneity). The average of the natural logarithm of the RRs was estimated and the RR from each study was weighted by the inverse of its variance. Linear dose–response analyses were conducted per 20 mmHg for systolic blood pressure and per 10 mmHg for diastolic blood pressure (consistent with previous studies [86–88]) using the method of Greenland and Longnecker [89]. For studies that reported blood pressure by ranges we estimated the midpoint for each category by calculating the average of the upper and lower cut-off points. For open-ended categories we used the width of the adjacent interval to estimate an upper or lower cut-off value for the extreme category. Fractional polynomial models were used to investigate a potential nonlinear association between systolic and diastolic blood pressure and risk of atrial fibrillation [90]. A log-likelihood test was used to test for nonlinearity [91].

Heterogeneity between studies was evaluated using Q and I^2^ statistics [92]. I^2^ is an estimate of how much of the heterogeneity that is due to between study variation rather than chance. I^2^-values of 25%, 50% and 75% indicates low, moderate and high heterogeneity respectively. We conducted main analyses (all studies combined) and stratified by study characteristics such as sample size, number of cases, whether prevalent cases were excluded or not, geographic location, study quality and by adjustment for confounding factors to investigate potential sources of heterogeneity and we used meta-regression analyses to test for differences in summary estimates between subgroups. Study quality was assessed using the Newcastle Ottawa scale which rates studies according to selection, comparability and outcome assessment with a score range from 0 to 9 [93].

Publication bias was assessed using Egger’s test [94] and by inspection of funnel plots. The statistical analyses were conducted using the software package Stata, version 13.1 software (StataCorp, Texas, US).

## Results

**Fig. 1:**
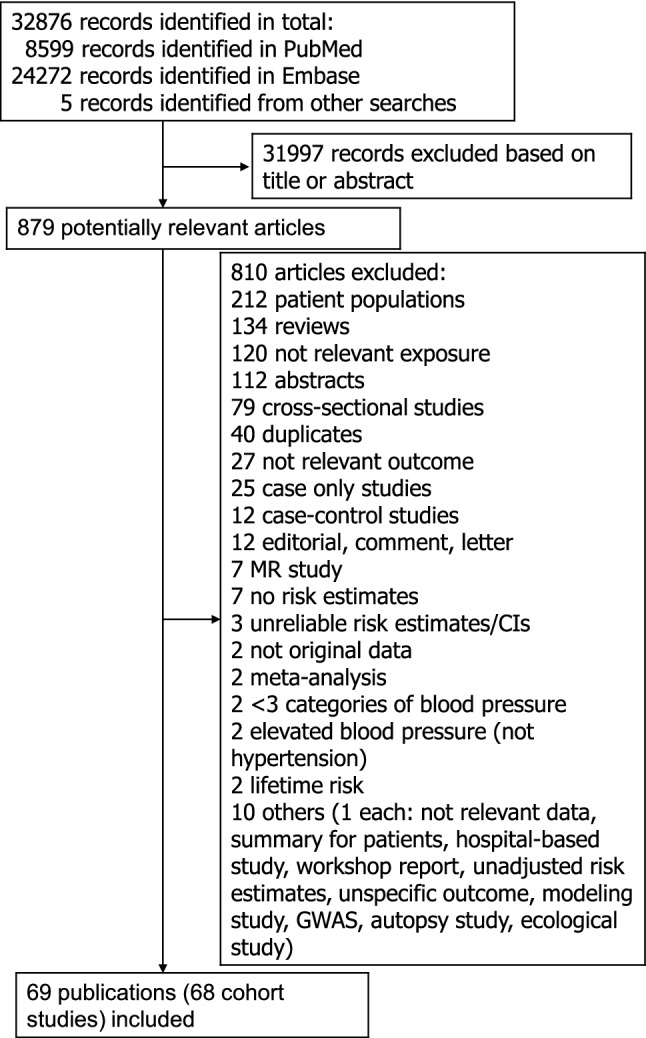
Flow-chart of study selection

From a total of 32,876 records that were identified by the search we included a total of 69 publications [7–9, 17–82] with data from 68 cohort studies (two of these were nested case–control studies within cohort studies [21, 66]) in the systematic review and meta-analysis of hypertension and blood pressure and atrial fibrillation (Fig. 1). Five of these publications were identified from separate searches on other risk factors for atrial fibrillation [9, 21, 32, 34, 42]. Each of two publications reported results from two studies combined [73, 81], and another publication reported results from six studies combined [65]. Two publications [42, 75] reported results from two separate studies each and one publication reported results from five separate studies [29], two of which were included in the main analysis (the other three were surpassed by more recent publications, but results of two of these duplicate studies were included in subgroup analyses by ethnicity). Twenty six studies (23 publications) [18, 20, 21, 25, 27–30, 33, 36, 42, 43, 48, 54, 64, 65, 69, 71–73, 75, 76, 78] were from Europe, twenty studies (25 publications) [7, 17, 19, 22, 24, 26, 29, 31, 34, 37, 40, 41, 45–47, 52, 53, 56, 59, 67, 68, 70, 74, 77, 81] were from North America, nineteen studies (19 publications) [23, 32, 35, 38, 39, 44, 49–51, 55, 57, 60–63, 66, 79, 80, 82] were from Asia, and three studies (3 publications) [8, 9, 58] were from Australia.

**Fig. 2:**
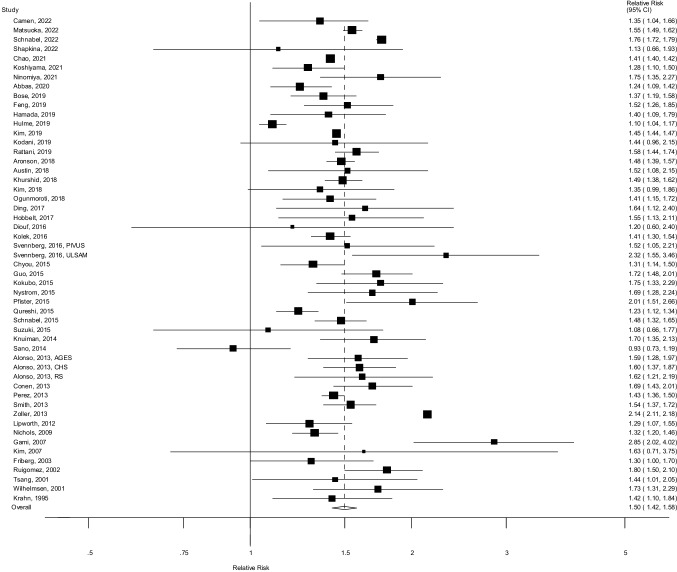
Hypertension and atrial fibrillation

Fifty six cohort studies (52 publications, 52 risk estimates) [7–9, 17–65] were included in the analysis of hypertension and atrial fibrillation risk including 1,080,611 cases and 30,539,230 participants (Fig. 1, Table 1). Data on hypertension and atrial fibrillation by ethnicity [29, 59] and sex [25] from three studies (ARIC, REGARDS, and Malmö Diet and Cancer Study) were only included in the respective subgroup analyses as the publications overlapped with more recent publications from the same studies which were used for the main analysis [27, 45, 56]. The summary relative risk for persons with hypertension compared to persons without hypertension was 1.50 (95% CI: 1.42–1.58, I^2^ = 98.1%, p_heterogeneity_ < 0.0001) (Fig. 2). There was no evidence of publication bias neither with Egger’s test (*p* = 0.74) or by inspection of the funnel plot (Supplementary Fig. 1). The summary RR ranged from 1.47 (95% CI: 1.42–1.52) when excluding the study by Zoller et al. [30] to 1.51 (95% CI: 1.43–1.59) when excluding the study by Sano et al. [53] (Supplementary Fig. 2).

**Table 1:** Cohort studies of hypertension and atrial fibrillation

First author, publication year, country	Study name or description	Study period	Number of participants, number of cases	Type of hypertension, subgroup	Comparison	Relative risk (95% confidence interval)	Adjustment for confounders
Krahn AD et al., 1995, Canada	Manitoba Follow-Up Study	1948–1992, 44 years follow-up	3983 men, age 18–62 years: 299 atrial fibrillation cases	Hypertension	Yes vs. no	1.42 (1.10–1.84)	Age
Wilhelmsen L et al., 2001, Sweden	Multifactor Primary Prevention Study	1970–1973–1996, 25.2 years follow-up	7495 men, age 47–55 years: 754 atrial fibrillation cases	Treatment for hypertension	Yes vs. no	1.73 (1.31–2.29)	Age
Tsang TSM et al., 2001, USA	Olmsted County–ECG	1990–1998–NA, 3.97 years follow-up	1655 men and women, age 65–105 years: 189 atrial fibrillation cases	Hypertension	Yes vs. no	1.44 (1.01–2.05)	Age, sex, history of valvular disease, congestive heart failure, myocardial infarction, diabetes mellitus
Ruigomez A et al., 2002, United Kingdom	UK General Practice Research Database	1996–1996, 1 year follow-up	1035 atrial fibrillation cases 5000 controls (nested case–control study) Men and women, age 40-89 years	Hypertension	Yes vs. no	1.80 (1.50–2.10)	Age, sex, cardiovascular morbidity
Friberg J et al., 2003, Denmark	Copenhagen City Heart Study	1981–1983 - 1991–1994–1994, 7 years follow-up	18,167 men and women, age 40–79 years: 379 atrial fibrillation cases	Arterial hypertension	Yes vs. no	1.3 (1.0–1.6)	Age, sex, prior myocardial infarction, diabetes mellitus, systolic blood pressure, ECG-LVH voltage, ECG-LVH ST/T, height, weight, BMI, FEV1, smoking status, alcohol, period
Gami AS et al., 2007, USA	Olmsted County Study	1987–2003, 4.7 years follow-up	3542 men and women, mean age 49 years: 133 atrial fibrillation cases	Hypertension	Yes vs. no	2.85 (2.02–4.02)	Age, sex, coronary artery disease, lowest nocturnal oxygen saturation
Kim HJ et al., 2007, Korea	Health Promotion Center, Samsung Medical Center	2001–2006, 3.7 years follow-up	16,568 men and women, median age 49 years: 61 atrial fibrillation cases	Hypertension medication use	Yes vs. no	1.63 (0.71–3.75)	Age, sex, history of coronary artery disease, BMI, fibrinogen, left atrial enlargement on ECG
Nichols GA et al., 2009, USA	Kaiser Permanente Northwest	1999–2008, 7.2 years follow-up	16,057 diabetes patients and 16,471 persons without diabetes, mean age 58.4 years: 1835 atrial fibrillation cases	Hypertension, all	Yes vs. no	1.32 (1.20–1.46)	Age, sex, race, smoking, BMI, systolic blood pressure, ischemic heart disease, valvular disease, hypertension, heart failure
				Hypertension, men	Yes vs. no	1.29 (1.13–1.46)	
				Hypertension, women	Yes vs. no	1.34 (1.15–1.55)	
Smith JG et al., 2010, Sweden	Malmo Diet and Cancer study	1991–1996–2005, 11.2 years follow-up	30,447 men and women, age 44–73 years: 1430 atrial fibrillation cases	Hypertension, men	Yes vs. no	1.78 (1.48–2.14)	Age
				Hypertension, women	Yes vs. no	1.74 (1.42–2.13)	
Lipworth L et al., 2012, USA	Southern Community Cohort Study	1999–2008, 5.7 years follow-up	8836 men and women, age ≥ 65 years: 1062 atrial fibrillation cases	Hypertension, all	Yes vs. no	1.29 (1.07–1.55)	Length of Medicare follow-up
				Hypertension, blacks	Yes vs. no	1.37 (1.05–1.80)	
				Hypertension, whites	Yes vs. no	1.19 (0.92–1.54)	
				Hypertension, men	Yes vs. no	1.18 (0.90–1.55)	
				Hypertension, women	Yes vs. no	1.38 (1.07–1.78)	
Smith JG et al., 2013, Sweden	Malmo Diet and Cancer study	1991–1996–2009, ~ 15.5 years follow-up	30,447 men and women, age 44–73 years: 2339 atrial fibrillation cases	Hypertension	Yes vs. no	1.54 (1.37–1.72)	Age, sex, BMI, history of myocardial infarction, diabetes, current smoking
Alonso A et al., 2013, USA	Cardiovascular Health Study	1989–1990, 1992–1993–2000, NA years of follow-up	5043 men and women, age 65 years: 624 atrial fibrillation cases	Hypertension, whites	Yes vs. no	1.63 (1.38–1.92)	Age, sex
				Hypertension, African Americans	Yes vs. no	1.31 (0.77–2.22)	
Alonso A et al., 2013, USA	Atherosclerosis Risk in Communities Study	1987–1989–1996–1998, -2005, NA years of follow-up	10,675 men and women, age 45–64 years: 419 atrial fibrillation cases	Hypertension, whites	Yes vs. no	2.02 (1.63.2.51)	Age, sex
				Hypertension, African Americans	Yes vs. no	2.31 (1.34–3.98)	
Alonso A et al., 2013, Iceland	Age, Gene/ Environment Susceptibility Reykjavik Study	2002–2011, NA years of follow-up	4469 men and women, mean age 76 years: 408 atrial fibrillation cases	Hypertension	Yes vs. no	1.59 (1.28–1.97)	Age, sex
Alonso A et al., 2013, Netherlands	Rotterdam Study	1997–2005, NA years of follow-up	3203 men and women, mean age 72 years: 177 atrial fibrillation cases	Hypertension	Yes vs. no	1.62 (1.21–2.19)	Age, sex
Conen D et al., 2013, USA	Women's Health Study	1992–1995–2011, 16.4 years follow-up	34,713 women, age ≥ 45 years: 796 atrial fibrillation cases	Hypertension, atrial fibrillation without LA enlargement	Yes vs. no	1.55 (1.25–1.92)	Age, height, body weight, diabetes, race, education, alcohol, smoking, exercise
				Hypertension, atrial fibrillation with LA enlargement	Yes vs. no	1.99 (1.49–2.65)	
Nyrnes A et al., 2013, Norway	Tromsø Study	1994–1995–2007, 11.1 years follow-up	22,815 men and women, age 25–96 years: 461/361 atrial fibrillation cases	Hypertension, men	Yes vs. no	1.98 (1.46–2.69)	Age, height, BMI, total cholesterol, HDL cholesterol, diabetes, palpitations, coronary artery disease
				Hypertension, women	Yes vs. no	1.40 (1.13–1.74)	
Zoller B et al., 2013, Sweden	Sweden Nationwide study	2000–2008, ~ 8 years follow-up	4,266,289 men and women, age ≥ 25 years: 101,985 atrial fibrillation cases	Hypertension, men	Yes vs. no	2.03 (1.99–2.07)	Age, neighborhood deprivation, marital status, family income, education, country of origin, urban/rural status, mobility, hospitalized for chronic lower respiratory disease, type 2 diabetes, alcohol, obesity, heart failure, coronary heart disease, hyperthyroidism
				Hypertension, women	Yes vs. no	2.32 (2.27–2.38)	
Perez MV et al., 2013, USA	Women's Health Initative	1994–1998–2007, 9.8 years follow-up	81,892 women, age 50–79 years: 8252 atrial fibrillation cases	Hypertension	Yes vs. no	1.43 (1.36–1.50)	Age, race/ethnicity, peripheral arterial disease, diabetes, hyperlipidemia, heart failure, coronary heart disease, BMI, postmenopausal hormone use, smoking status, alcohol, education
Knuiman M et al., 2014, Australia	Busselton Health Study	1994–1995–NA, 15 years follow-up	4267 men and women, age 25–84 years: 343 atrial fibrillation cases	Hypertension treatment	Yes vs. no	1.70 (1.35–2.13)	Age, sex, height
Sano F et al., 2014, Japan	Circulatory Risk in Communities Study	1991–1995–2000, 6.4 years follow-up	8602 men and women, age 30–80 years: 296 atrial fibrillation cases	Hypertension, all	Yes vs. no	0.93 (0.73–1.19)	Age, sex, alcohol, smoking status, BMI, hyperglycemia, hyperlipidemia, major ST-T abnormality, previous MI, heart failure
				Hypertension, men	Yes vs. no	1.02 (0.69–1.50)	
				Hypertension, women	Yes vs. no	0.89 (0.66–1.22)	
Pfister R et al., 2015, United Kingdom	EPIC-Norfolk	1993–1997–2009, 5 years follow-up (analysis restricted to first 5 years)	24,020 men and women, age 39–79 years: 236 atrial fibrillation cases	Hypertension treatment	Yes vs. no	2.01 (1.51–2.66)	Age, height, weight, current smoking, systolic blood pressure, diastolic blood pressure, diabetes mellitus, heart failure, myocardial infarction
Schnabel RB et al., 2015, USA	Framingham Cohort Study	1958–2007, 50 years follow-up	9511 men and women, age 50–89 years: 1544 atrial fibrillation cases	Hypertension, 1958–1967	Yes vs. no	1.71 (0.96–3.06)	Age, sex
				Hypertension, 1968–1977	Yes vs. no	1.63 (1.18–2.27)	
				Hypertension, 1978–1987	Yes vs. no	1.35 (1.06–1.71)	
				Hypertension, 1988–1997	Yes vs. no	1.68 (1.37–2.06)	
				Hypertension, 1998–2007	Yes vs. no	1.32 (1.08–1.60)	
Suzuki H et al., 2015, Japan	Fukushima Health Management Survey	2008–2010–2013, 1.4 years follow-up	12,410 men and women, age 40–90 years: 79 atrial fibrillation cases	Hypertension	Yes vs. no	1.08 (0.66–1.77)	Age, sex, obesity, excess ethanol intake, current smoking, hypertension
Nystrom PK et al., 2015, Sweden	Stockholm—60 year old men and women	1997–1999–2012, 13.6 years follow-up	4021 men and women, age 60 years: 285 atrial fibrillation cases	Hypertension, normal waist	Yes vs. no	2.54 (1.54–4.21)	Sex, birth country, smoking status, alcohol, moderate-intensity exercise, history of myocardial infarction
				Hypertension, medium waist	Yes vs. no	1.92 (1.10–3.33)	
				Hypertension, elevated waist	Yes vs. no	1.18 (0.77–1.79)	
Qureshi WT et al., 2015, USA	Henry Ford Exercise Testing (FIT) Project	1991–2009, 5.4 years follow-up	64,561 men and women, mean age 54.5 years: 4616 atrial fibrillation cases	Hypertension	Yes vs. no	1.23 (1.12–1.34)	Age, sex, race/ethnicity, hypertension, sedentary, obesity, family history of coronary artery disease, smoking, history of hyperlipidemia, known coronary artery disease, hypertensive response, thyroid medication use, digoxin use, lung medication use, beta blocker use, ACE inhibitor use, ARB inhibitor, calcium channel blocker use, lipid lowering medication use, METs
Guo Y et al., 2015, China	Yunnan Medical Insurance Database	2001–2012, 11 years follow-up	471,446 men and women, age ≥ 20 years: 866 atrial fibrillation cases	Hypertension	Yes vs. no	1.72 (1.48–2.01)	Age, sex, rheumatic heart disease, dilated cardiomyopathy, heart failure, hyperthyroidism, coronary artery disease, chronic obstructive pulmonary disease, diabetes, renal dysfunction, hyperlipidemia, peripheral vascular disease
Kokubo Y et al., 2015, Japan	Suita Study	1989–2006–2015, 12.8 years follow-up	6906 men and women, age 30–79 years: 253 atrial fibrillation cases	Hypertension	< 120/ < 80 mmHg	1.00	Age, sex, BMI, hypercholesterolemia, diabetes, smoking status, drinking status, cohort groups, chronic kidney disease, stroke, coronary heart disease, chronic heart failure, premature contractions
					120–139/80–89	1.20 (0.83–1.73)	
					≥ 140/ ≥ 90	1.53 (1.07–2.19)	
Chyou JY et al., 2015, USA	Truven Health MarketScan Commercial and Medicare Supplemental Databases	2007–2010, 3 years follow-up	3,007,874 men and women, all ages: 165,741 atrial fibrillation cases	Hypertension	Yes vs. no	1.31 (p < 0.0001)	Age, sex, heart failure, diabetes, coronary artery disease, chronic kidney disease, sleep apnea, chest pain, dizziness, palpitations, tachycardia unspecified, shortness of breath, respiratory other, respiratory—unspecified, geographic region, medications, use of internal/ external electrocardiographic recording device
Diouf I et al., 2016, Australia	Australian Diabetes, Obesity and Lifestyle study cohort	1999/2000–2004/2005, 5 years follow-up	5389 men and women, age ≥ 35 years: 53 atrial fibrillation cases	Hypertension	Yes vs. no	1.20 (0.60–2.40)	Age, sex, smoking status, usual number of alcoholic drinks, physical activity, level of education, BMI
Kolek MJ et al., 2016, USA	Vanderbilt University Medical Center Electronic Medical Records	2005–2010, 5 years follow-up	33,494 men and women, age ≥ 40 years: 2455 atrial fibrillation cases	Hypertension treatment	Yes vs. no	1.41 (1.30–1.54)	Age, race, height, weight, smoking status, systolic blood pressure, diastolic blood pressure, diabetes, heart failure, myocardial infarction
Svennberg E et al., 2016, Sweden	Uppsala Longitudinal Study of Adult Men (ULSAM)	1970—NA, 12.6 years follow-up	883 men, age 50 years: 113 atrial fibrillation cases	Antihypertensive treatment	Yes vs. no	2.32 (1.55–3.46)	Age, sex, smoking, BMI, systolic blood pressure, total cholesterol, HDL cholesterol, lipid-lowering treatment, type 2 diabetes, heart failure
Svennberg E et al., 2016, Sweden	Prospective Investigation of the Vasculature in Uppsala Seniors (PIVUS)	2001—NA, 10 years follow-up	978 men and women, age 70 years: 148 atrial fibrillation cases	Antihypertensive treatment	Yes vs. no	1.52 (1.05–2.21)	Age, sex, smoking, BMI, systolic blood pressure, total cholesterol, HDL cholesterol, lipid-lowering treatment, type 2 diabetes
O'Neal WT et al., 2017, USA	Reasons for Geographic And Racial Differences in Stroke (REGARDS) Study	2003–2007—NA, 9.4 years follow-up	13,688 men and women, mean age 63 years: 997 atrial fibrillation cases	Hypertension, whites	Yes vs. no	1.28 (1.11–1.48)	Age, sex, ethnicity, income, smoking status, diabetes, obesity, exercise, dyslipidemia, left ventricular hypertrophy, cardiovascular disease
				Hypertension, blacks	Yes vs. no	1.24 (0.90–1.71)	
Ding L et al., 2017, China	Shandong Multi-Center Health Check-up Longitudinal Study	2004–2014, 2.6 years follow-up	33,186 men and women, age 45–85 years: 134 atrial fibrillation cases	Hypertension	Yes vs. no	1.64 (1.12–2.40)	Age, sex
Hobbelt AH et al., 2017, Netherlands	PREVEND Study	1997—NA, NA	8042 men and women, mean age 48.5 years: 319 atrial fibrillation cases	Hypertension (anti-hypertensive treatment), atrial fibrillation without 2-year recurrence	Yes vs. no	1.50 (0.88–2.56)	Age, sex, BMI, heart rate, lipid-lowering treatment, MR-proANP, eGFR
				Hypertension (anti-hypertensive treatment), self-terminating atrial fibrillation	Yes vs. no	2.52 (1.19–5.33)	
				Hypertension (anti-hypertensive treatment), non-self-terminating atrial fibrillation	Yes vs. no	1.33 (0.85–2.08)	
Ogunmoroti O et al., 2018, USA	Multi-Ethnic Study of Atherosclerosis	2000–2002—NA, 11.2 years follow-up	6506 men and women, age 45–84 years: 709 atrial fibrillation cases	Hypertension/blood pressure, all	< 120/ < 80 mmHg	0.71 (0.58–0.87)	Age, sex, race/ethnicity, education, income, health insurance
					120–139/80–89	0.86 (0.72–1.03)	
					≥ 140/ ≥ 90	1.00	
				Hypertension/blood pressure, whites	< 120/ < 80 mmHg 120–139/80–89 ≥ 140/ ≥ 90	0.70 (0.53–0.93) 0.86 (0.67–1.11) 1.00	
				Hypertension/blood pressure, Chinese Americans	< 120/ < 80 mmHg 120–139/80–89 ≥ 140/ ≥ 90	0.73 (0.42–1.29) 1.17 (0.72–1.91) 1.00	
				Hypertension/blood pressure, African Americans	< 120/ < 80 mmHg 120–139/80–89 ≥ 140/ ≥ 90	0.76 (0.46–1.25) 0.89 (0.60–1.31) 1.00	
				Hypertension/blood pressure, Hispanic	< 120/ < 80 mmHg 120–139/80–89 ≥ 140/ ≥ 90	0.64 (0.40–1.02) 0.72 (0.47–1.09) 1.00	
Austin TR et al., 2018, USA	Jackson Heart Study	2005–2008–2012, 8.5 years follow-up	5240 men and women, mean age 55 years: 242 atrial fibrillation cases	Hypertension (antihypertensive medication)	Yes vs. no	1.52 (1.08–2.15)	Age, sex, height, weight, glycemic status, history of myocardial infarction, FEV_1_, eGFR, ECG PR interval, current smoking, insured, high school graduate
Aronson D et al., 2018, USA	Maccabi Health Services	2005–2015, 9.7 years follow-up	96,778 men and women, age ≥ 50 years: 5660 atrial fibrillation cases	Hypertension	Yes vs. no	1.48 (1.39–1.57)	Age, sex, BMI, systolic blood pressure, history of myocardial infarction, peripheral arterial disease, history of heart failure, chronic obstructive pulmonary disease, inflammatory disease, age at prevalent heart failure, female with inflammatory disease
Khurshid S et al., 2018, United Kingdom	UK Biobank	2006–2010–2015–2016, 7 years follow-up	489,194 men and women, age 40–69 years: 10,619 atrial fibrillation cases	Hypertension	Yes vs. no	1.49 (1.38–1.62)	Age, sex, race, BMI, smoking status, alcohol, hyperlipidemia, diabetes, chronic kidney disease, sleep apnea, asthma, chronic obstructive pulmonary disease, hyperthyroidism, hypothyroidism, depression, venous thromboembolism, peripheral arterial disease, stroke, coronary artery disease, heart failure
Kim YG et al., 2018, Korea	Ulsan University Hospital	2003–2008–2016, 8.7 years follow-up	21,813 men, mean age 45.9 years: 168 atrial fibrillation/ flutter cases	Hypertension	Yes vs. no	1.35 (0.99–1.86)	Age, smoking status, regular exercise, alcohol drinking, chronic kidney disease, central obesity, raised fasting glucose, raised triglycerides, reduced HDL-cholesterol
Kodani E et al., 2019, Japan	TAMA MED Project-AF	2008–2015, 6.9 years follow-up	10,430 men and women, age 40–74 years: 133 atrial fibrillation cases	Hypertension	Yes vs. no	1.44 (0.96–2.15)	Age, sex, cardiac disease, diabetes, LDL-cholesterol, triglycerides, GGTP, smoking status
Hamada R et al., 2019, Japan	Seirei Center for Health Promotion and Preventive Medicine, Hamamatsu City	2008–2014–2015, 7 years follow-up	65,984 men and women, age 40–79 years: 349 atrial fibrillation cases	Hypertension (antihypertensive agent use)	Yes vs. no	1.40 (1.09–1.79)	Age, sex
Bose A et al., 2019, USA	Reasons for Geographic and Racial Differences in Stroke [REGARDS] Study	2003–2007—NA, 9.3 years follow-up	11,806 men and women, mean age 63 years: 1016 atrial fibrillation cases	Hypertension (use of blood pressure medication), men	Yes vs. no	1.46 (1.22–1.76)	Age, race, height, weight, cardiovascular disease
				Hypertension (use of blood pressure medication), women	Yes vs. no	1.25 (1.00–1.56)	
Hulme OL et al., 2019, USA	Partners HealthCare System Research Patient Data Registry	2000–2014, NA	206,042 men and women, age 45–95 years: 7216 atrial fibrillation cases	Hypertension	Yes vs. no	1.11 (1.05–1.18)	Age, age squared, sex, ethnicity/race, smoking, height, height squared, weight, weight squared, diastolic blood pressure, hyperlipidemia, heart failure, coronary heart disease, valvular disease, previous stroke/TIA, peripheral artery disease, chronic kidney disease, hypothyroidism
Feng T et al., 2019, Norway	HUNT	2006–2008–2015, 8.1 years follow-up	47,870 men and women, age ≥ 20 years: 1758 atrial fibrillation cases	Hypertension	No, BMI < 25.0	1.0	Age, sex, height, smoking status, time since last meal, type of work, marital status, physical activity, alcohol, C-reactive protein, sex, blood glucose, triglycerides, HDL-cholesterol, abdominal obesity
					Yes, BMI ≥ 25.0	1.6 (1.2–2.1)	
					No, BMI 25.0–29.9	1.3 (1.0–1.7)	
					Yes, BMI 25.0–29.9	1.7 (1.4–2.2)	
					No, BMI < 30	1.3 (0.9–1.9)	
					Yes, BMI ≥ 30	2.1 (1.6–2.8)	
Kim YG et al., 2019, Korea	Korea National Health Insurance Database	NA-NA, 8.2 years follow-up	9,797,418 men and women, mean age 47 years: 196,136 atrial fibrillation cases	Hypertension, age 20- < 30	Non-hypertension	1.00	Age, sex, smoking status, alcohol, regular physical activity, low income, diabetes mellitus, dyslipidemia
					Pre-hypertension	1.14 (1.07–1.22)	
					Hypertension with med., <5 years	1.37 (1.19–1.58)	
					Hypertension with med., <5 years	3.67 (2.83–4.76)	
					Hypertension with med., ≥ 5 years	6.22 (3.74–10.36)	
				Hypertension, age 30- < 40	Non-hypertension	1.00	
					Pre-hypertension	1.15 (1.09–1.20)	
					Hypertension without medication	1.42 (1.32–1.53)	
					Hypertension with medication, < 5 years	2.72 (2.48–3.00)	
					Hypertension with medication, ≥ 5 years	3.40 (2.88–4.02)	
				Hypertension, age 40- < 50	Non-hypertension	1.00	
					Pre-hypertension	1.10 (1.07–1.13)	
					Hypertension without medication	1.27 (1.21–1.32)	
					Hypertension with medication, < 5 years	1.84 (1.76–1.93)	
					Hypertension with medication, ≥ 5 years	2.13 (2.01–2.25)	
				Hypertension, age 50- < 60	Non-hypertension	1.00	
					Pre-hypertension	1.05 (1.02–1.08)	
					Hypertension without medication	1.15 (1.10–1.19)	
					Hypertension with medication, < 5 years	1.53 (1.49–1.58)	
					Hypertension with medication, ≥ 5 years	1.76 (1.70–1.81)	
				Hypertension, age 60- < 70	Non-hypertension	1.00	
					Pre-hypertension	1.01 (0.99–1.04)	
					Hypertension without medication	1.11 (1.07–1.15)	
					Hypertension with medication, < 5 years	1.38 (1.34–1.43)	
					Hypertension with medication, ≥ 5 years	1.66 (1.61–1.70)	
				Hypertension, age ≥ 70	Non-hypertension	1.00	
					Pre-hypertension	0.99 (0.96–1.02)	
					Hypertension without medication	1.06 (1.02–1.10)	
					Hypertension with medication, < 5 years	1.30 (1.26–1.35)	
					Hypertension with medication, ≥ 5 years	1.54 (1.49–1.58)	
Rattani A et al., 2019, USA	Atherosclerosis Risk in Communities Study	1987–2017, 21.4 years follow-up	14,915 men and women, age 45–64 years: 2891 atrial fibrillation cases	Hypertension categories (JNC 7)	Normal	1.00	Age, sex, race, height, education, field center, BMI, smoking, drinking status, diabetes, heart failure, coronary heart disease, stroke
					Prehypertension	1.24 (1.12–1.36)	
					Hypertension	1.58 (1.44–1.74)	
				Hypertension (2017 ACC/AHA)	Yes vs. no	1.37 (1.26–1.48)	
				Hypertension categories (2017 ACC/AHA)	Normal	1.00	
					Elevated	1.26 (1.11–1.43)	
					Stage 1 hypertension	1.21 (1.07–1.37)	
					Stage 2 hypertension	1.58 (1.44–1.74)	
Abbas SS et al., 2020, Australia	Australian Longitudinal Study on Women's Health	2000–2015, NA	6671 women, mean age 77.79 years: 1827 atrial fibrillation cases	Hypertension	Yes vs. no	1.24 (1.09–1.42)	Exercise, heart attack, angina, arthritis
Koshiyama M et al., 2021, Japan	Iwate Prefecture	2010–2013, 3 years follow-up	130,396 men and women, age 45- ≥ 85 years: 824 atrial fibrillation cases	Hypertension, men	Yes vs. no	1.20 (1.01–1.43)	Age, diabetes mellitus, dyslipidemia, overweight, coronary artery disease, stroke, smoking, drinking, regular exercise
				Hypertension, women	Yes vs. no	1.70 (1.29–2.45)	
Ninomiya Y et al., 2021, Japan	JA Kagoshima Kouseiren Medical Health Care Center	2008–2016, 5 years follow-up	67,379 men and women, mean age 54 years: 280 atrial fibrillation cases	Hypertension, all	Yes vs. no	1.75 (1.35–2.27)	Age, waist circumference, diabetes, dyslipidemia, drinking, chronic kidney disease
				Hypertension, men	Yes vs. no	1.46 (1.09–1.95)	
				Hypertension, women	Yes vs. no	2.69 (1.52–4.77)	
Chao TF et al., 2021, Taiwan	Taiwan Health Insurance Database	2000–2016, 16 years follow-up	7,220,654 men and women, age 40 years: 438,930 atrial fibrillation cases	Hypertension	Yes vs. no	1.41 (1.40–1.42)	Age, sex, diabetes mellitus, heart failure, stroke, coronary artery disease with or without myocardial infarction, peripheral vascular diseases, COPD, autoimmune diseases, liver cirrhosis, hyperthyroidism, chronic kidney disease with or without end-stage renal disease, gout, alcoholism
Matsuoka S et al., 2022, Japan	JMDC Claims Database	2005–2020, 3.3 years follow-up	2,597,441 men and women, age 20–75 years: 12,773 atrial fibrillation cases	Hypertension, age 20–49 years	Yes vs. no	1.68 (1.45–1.82)	Age, sex, obesity, high waist circumference, diabetes, dyslipidemia, cigarette smoking, alcohol, physical inactivity
				Hypertension, age 50–59 years	Yes vs. no	1.60 (1.50–1.69)	
				Hypertension, 60–75 years	Yes vs. no	1.45 (1.35–1.55)	
Shapkina M et al., 2022, Russia	HAPIEE study	2003–2005–2017, 12.85 years follow-up	3871 men and women, age 45–69 years: 122 atrial fibrillation cases	Hypertension	Yes vs. no	1.13 (0.66–1.93)	Age, BMI, total cholesterol, triglycerides, smoking, alcohol, education, marital status
Schnabel RB et al., 2022, Germany	InGef research database	2013–2016,	1,476,391 men and women, age ≥ 45 years: 98,958 atrial fibrillation cases	Hypertension, treated	Yes vs. no	1.76 (1.72–1.79)	Age, sex, treated heart failure, valvular heart disease, chronic kidney disease, stroke—not specified as hemorrhage or infarction, hemiplegia, other pulmonary heart diseases, paroxysmal tachycardia, other cardiac arrhythmias, ulcer of lower limb not elsewhere classified, personal history of medical treatment
Camen S et al., 2022, Europe	6 cohort studies: DAN-MONICA study FINRISK study Moli-sani study Northern Sweden MONICA study Scottish Heart Health Extended Cohort (SHHEC) The Tromsø Study	1982–2010, max 10 years follow-up	108,363 men and women, median age 46.0 years: 2413 atrial fibrillation cases	Antihypertensive treatment	Yes vs. no	1.35 (1.04–1.66)	Age, sex, cohort, BMI, total serum cholesterol, diabetes, daily smoking, systolic blood pressure

*ARB* = angiotensin receptor blocker, *ACE* = angiotensin converting enzyme, *BMI* = body mass index, *ECG* = electrocardiography, *eGFR* = estimated glomerular filtration rate, *FEV1* = forced expiratory volume per 1 s, *GGTP* = gamma glutamyl transpeptidase, *HDL* = high-density lipoprotein, *LVH* = left ventricular hypertrophy, *MET* = metabolic equivalent task, *MR-proANP* = mid-regional pro atrial natriuretic peptide, *NA* = not available, *PR* = pulse rate, *ST/T* = ST and T wave ratio, *TIA* = transient ischemic attack

**Fig. 3:**
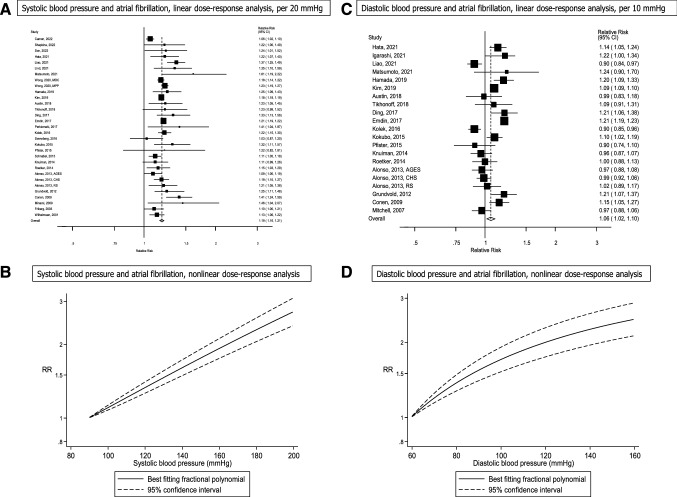
Systolic and diastolic blood pressure and atrial fibrillation, linear and nonlinear dose–response analysis

Thirty seven cohort studies (28 publications, 31 risk estimates) [8, 18, 20, 29, 33, 34, 39, 41, 42, 44, 46, 51, 55, 63, 65–67, 69–75, 77–80] (347,813 cases, 14,565,763 participants) were included in the analysis of systolic blood pressure and atrial fibrillation (Table 2). The summary RR was 1.19 (95% CI: 1.16–1.21, I^2^ = 68.4%, p_heterogeneity_ < 0.0001) per 20 mmHg increment (Fig. 3a). There was no evidence of publication bias with Egger's test (*p* = 0.46), but some indication of asymmetry in the funnel plot (Supplementary Fig. 3a). When using the trim and fill method, eight studies were added, but the results were similar, summary RR = 1.17 (95% CI: 1.14–1.19) (Supplementary Fig. 4). The summary RR ranged from 1.18 (95% CI: 1.16–1.20) when the Atherosclerosis Risk in Communities Study [77] was excluded to 1.20 (95% CI: 1.18–1.22) when a pooled analysis [65] was excluded (Supplementary Fig. 5). Ten cohort studies [18, 34, 39, 55, 67–69, 76, 79, 80] were included in the nonlinear dose–response analysis. Although the test for nonlinearity was significant, p_nonlinearity_ < 0.0001, the association was approximately linear, and there was a dose-dependent increase in risk with increasing systolic blood pressure from a systolic blood pressure level of 90 mmHg and above (Fig. 3b).

**Table 2:** Cohort studies of blood pressure and atrial fibrillation

First author, publication year, country	Study name or description	Study period	Number of participants, number of cases	Exposure, subgroup	Comparison	Relative risk (95% confidence interval)	Adjustment for confounders
Wilhelmsen L et al., 2001, Sweden	The Multifactor Primary Prevention Study	1970–1973–1996, 25.2 years follow-up	7495 men, age 47–55 years: 754 atrial fibrillation cases	SBP	≤ 145 mmHg 146–175 ≥ 176	1.00 1.30 (1.11–1.54) 1.37 (1.09–1.74)	Age
Friberg J et al., 2003, Denmark	Copenhagen City Heart Study	1981–1983 - 1991–1994–1994, 7 years follow-up	18,167 men and women, age 40–79 years: 379 atrial fibrillation cases	SBP	Per 10 mmHg	1.0 (1.0–1.1)	Age, sex, prior myocardial infarction, diabetes mellitus, arterial hypertension, ECG-LVH voltage, ECG-LVH ST/T, height, weight, BMI, FEV1, smoking status, alcohol, period
Mitchell GF et al., 2007, USA	Framingham Heart Study and Framingham Offspring Study	1979–1982/ 1983–2004, 16 years follow-up	5331 men and women, age ≥ 35 years: 698 atrial fibrillation cases	DBP	Per 10 mmHg	0.97 (0.88–1.06)	Age, sex, BMI, smoking, valvular disease, myocardial infarction, heart failure, diabetes, ECG—left ventricular hypertrophy, hypertension treatment
Conen D et al., 2009, USA	Women's Health Study	1993–2006, 12.4 years follow-up	34,221 women, age ≥ 45 years: 644 atrial fibrillation cases	SBP	< 120 mmHg 120–129 130–139 140–159 ≥ 160	1.00 1.00 (0.78–1.28) 1.28 (1.00–1.63) 1.56 (1.22–2.01) 2.74 (1.77–4.22)	Age, BMI, diabetes, smoking, hypercholesterolemia, exercise, alcohol, education, randomized treatment assignment + mutual adjustment between systolic and diastolic blood pressure
				DBP	< 65 mmHg 65–74 75–84 85–89 90–94 ≥ 95	1.00 1.17 (0.81–1.69) 1.18 (0.84–1.65) 1.53 (1.05–2.23) 1.35 (0.82–2.22) 2.15 (1.21–3.84)	
				SBP	< 120 mmHg 120–129 130–139 140–159 ≥ 160	1.00 1.01 (0.77–1.33) 1.30 (0.97–1.74) 1.58 (1.16–2.16) 2.69 (1.63–4.46)	
				DBP	< 65 mmHg 65–74 75–84 85–89 90–94 ≥ 95	1.00 1.14 (0.78–1.66) 1.02 (0.70–1.49) 1.11 (0.72–1.71) 0.91 (0.53–1.58) 1.22 (0.64–2.33)	
Minami M et al., 2009, Japan	Ishikawa Prefecture	1998–2006, NA	69 atrial fibrillation cases (men) 138 controls (nested case–control study)	SBP	Per 1 mmHg	1.019 (1.002–1.037)	Age, time period, BMI, total cholesterol, GGTP, uric acid, fasting plasma glucose, hemoglobin, cardiomegaly, Brinkman index, drinking habits
Chamberlain AM et al., 2011, USA	Atherosclerosis Risk in Communities Study	1987–1989–1998, 10 years follow-up	14,546 men and women, age 45–64 years: 515 atrial fibrillation cases	SBP	< 100 mmHg 100- < 120 120- < 140 140- < 160 ≥ 160	0.82 (0.53–1.28) 1.00 1.42 (1.15–1.76) 2.16 (1.67–2.79) 2.63 (1.83–3.78)	Age, sex, race
				DBP	< 70 mmHg 70- < 80 80- < 90 90 < 100 ≥ 100	0.93 (0.75–1.15) 1.00 1.24 (0.98–1.57) 1.53 (1.06–2.22) 2.02 (1.20–3.41)	
Grundvold I et al., 2012, Norway	Oslo 1972–1975	1972–1975–2007, 30 years follow-up	2014 men, age 40–59 years: 270 atrial fibrillation cases	SBP	88–116 mmHg 118–126 128–138 140–220 Per 18 mmHg	1.00 1.26 (0.74–2.14) 1.98 (1.22–3.27) 1.84 (1.07–3.19) 1.22 (1.10–1.42)	Age, BMI, left ventricular hypertrophy, smoking, total cholesterol, physical fitness, resting heart rate
				DBP	54–78 mmHg 80–86 88–92 94–130 Per 10 mmHg	1.00 1.67 (1.00–2.85) 1.76 (1.01–3.11) 2.36 (1.38–4.15) 1.25 (1.11–1.43)	
Alonso A et al., 2013, USA	Cardiovascular Health Study	1989–1990, 1992–1993–2000, NA years of follow-up	5043 men and women, age 65 years: 624 atrial fibrillation cases	SBP, whites	Per 20 mmHg	1.19 (1.10–1.28)	Age, sex
				SBP, African Americans	Per 20 mmHg	1.15 (0.92–1.42)	
				DBP, whites	Per 10 mmHg	1.00 (0.93–1.08)	
				DBP, African Americans	Per 10 mmHg	0.90 (0.72–1.13)	
Alonso A et al., 2013, USA	Atherosclerosis Risk in Communities Cohort	1987–1989–1996–1998, -2005, NA years of follow-up	10,675 men and women, age 45–64 years: 419 atrial fibrillation cases	SBP, whites	Per 20 mmHg	1.14 (1.02–1.28)	Age, sex
				SBP, African Americans	Per 20 mmHg	0.98 (0.78–1.24)	
				DBP, whites	Per 10 mmHg	0.89 (0.80–0.99)	
				DBP, African Americans	Per 10 mmHg	1.04 (0.84–1.29)	
Alonso A et al., 2013, Iceland	Age, Gene/ Environment Susceptibility Reykjavik Study	2002–2011, NA years of follow-up	4469 men and women, mean age 76 years: 408 atrial fibrillation cases	SBP	Per 20 mmHg	1.09 (1.00–1.19)	Age, sex
				DBP	Per 10 mmHg	0.97 (0.88–1.08)	
Alonso A et al., 2013, Netherlands	Rotterdam Study	1997–2005, NA years of follow-up	3203 men and women, mean age 72 years: 177 atrial fibrillation cases	SBP	Per 20 mmHg	1.21 (1.05–1.38)	Age, sex
				DBP	Per 10 mmHg	1.02 (0.89–1.17)	
Roetker NS et al., 2014, USA	Multi-Ethnic Study of Atherosclerosis	2000–2002–2009, 7.8 years follow-up	6630 men and women, age 45–84 years: 307 atrial fibrillation cases	SBP	Per 21.5 mmHg	1.16 (1.03–1.31)	Age, sex, race/ethnicity, site, education, height, BMI, smoking status, antihypertensive medication use, diabetes, ECG-based LVH, P-R interval, resting heart rate
				DBP	Per 10.3 mmHg	1.00 (0.88–1.13)	
Knuiman M et al., 2014, Australia	Busselton Health Study	1994–1995–2010, 15 years follow-up	4267 men and women, age 25–84 years: 343 atrial fibrillation cases	SBP	Per 17.8 mmHg	1.10 (0.99–1.23)	Age, sex, height + hypertension treatment, BMI
				DBP	Per 10 mmHg	1.04 (0.93–1.15)	
				SBP	Per 17.8 mmHg	1.02 (0.91–1.14)	
				DBP	Per 10 mmHg	0.96 (0.87–1.07)	
Schnabel RB et al., 2015, USA	Framingham Cohort Study	1958–2007, 50 years follow-up	9511 men and women, age 50–89 years: 1544 atrial fibrillation cases	SBP, 1958–1967	< 120 mmHg 120–129 130–139 140–159 ≥ 160	1.00 1.76 (0.59–5.25) 1.29 (0.42–3.95) 1.95 (0.73–5.21) 2.63 (1.00–6.93)	Age, sex
				SBP, 1968–1977	< 120 mmHg 120–129 130–139 140–159 ≥ 160	1.00 0.53 (0.26–1.07) 0.79 (0.43–1.45) 1.39 (0.83–2.34) 1.36 (0.79–2.32)	
				SBP, 1978–1987	< 120 mmHg 120–129 130–139 140–159 ≥ 160	1.00 0.75 (0.46–1.21) 0.97 (0.63–1.49) 1.16 (0.79–1.72) 1.21 (0.79–1.85)	
				SBP, 1988–1997	< 120 mmHg 120–129 130–139 140–159 ≥ 160	1.00 1.11 (0.76–1.61) 1.27 (0.89–1.81) 1.47 (1.06–2.03) 1.28 (0.89–1.83)	
				SBP, 1998–2007	< 120 mmHg 120–129 130–139 140–159 ≥ 160	1.00 0.94 (0.69–1.29) 1.19 (0.89–1.60) 0.89 (0.67–1.19) 1.15 (0.84–1.58)	
Pfister R et al., 2015, United Kingdom	EPIC-Norfolk	1993–1997–2009, 5 years follow-up (analysis restricted to first 5 years)	24,020 men and women, age 39–79 years: 236 atrial fibrillation cases	SBP	Per 1 mmHg	1.01 (0.99–1.03)	Age, height, weight, current smoking, hypertension treatment, diabetes mellitus, heart failure, myocardial infarction
				DBP	Per 1 mmHg	0.99 (0.97–1.01)	
Kokubo Y et al., 2015, Japan	Suita Study	1989–2006–2015, 12.8 years follow-up	6906 men and women, age 30–79 years: 253 atrial fibrillation cases	SBP	< 120 mmHg 120–139 ≥ 140	1.00 1.29 (0.91–1.85) 1.74 (1.22–2.49)	Age, sex, BMI, hypercholesterolemia, diabetes, smoking status, drinking status, cohort groups, chronic kidney disease, stroke, coronary heart disease, chronic heart failure, premature contractions + mutual adjustment between systolic and diastolic blood pressure
				DBP	< 80 mmHg 80–89 ≥ 90	1.00 1.16 (0.84–1.61) 1.47 (1.08–1.99)	
				SBP	< 120 mmHg 120–139 ≥ 140	1.00 1.29 (0.88–1.90) 1.74 (1.12–2.69)	
				DBP	< 80 mmHg 80–89 ≥ 90	1.00 1.03 (0.73–1.46) 1.14 (0.77–1.69)	
Svennberg E et al., 2016, Sweden	Prospective Investigation of the Vasculature in Uppsala Seniors (PIVUS)	2001—NA, 10 years follow-up	978 men and women, age 70 years: 148 atrial fibrillation cases	SBP	Per 22.5 mmHg	1.03 (0.86–1.23)	Age, sex, smoking, BMI, antihypertensive treatment, total cholesterol, HDL cholesterol, lipid-lowering treatment, type 2 diabetes
Kolek MJ et al., 2016, USA	Vanderbilt University Medical Center Electronic Medical Records	2005–2010, 5 years follow-up	33,494 men and women, age ≥ 40 years: 2455 atrial fibrillation cases	SBP	Per 20 mmHg	1.22 (1.15–1.30)	Age, race, height, weight, smoking status, systolic blood pressure, diastolic blood pressure, diabetes, heart failure, myocardial infarction
				DBP	Per 10 mmHg	0.90 (0.85–0.96)	
Emdin CA et al., 2017, United Kingdom	UK General Practice Research Database	1990–2013—NA, 6.9 years follow-up	4,301,349 men and women, age 30–90 years: 128,468 atrial fibrillation cases	SBP, all	Per 20 mmHg	1.21 (1.19–1.22)	Age, sex, smoking status, diabetes
				SBP, men	Per 20 mmHg	1.16 (1.14–1.18)	
				SBP, women	Per 20 mmHg	1.26 (1.24–1.28)	
				DBP, all	Per 10 mmHg	1.21 (1.19–1.23)	
				DBP, men	Per 10 mmHg	1.24 (1.21–1.26)	
				DBP, women	Per 10 mmHg	1.19 (1.16–1.21)	
Ding L et al., 2017, China	Shandong Multi-Center Health Check-up Longitudinal Study	2004–2014, 2.6 years follow-up	33,186 men and women, age 45–85 years: 134 atrial fibrillation cases	SBP Diastolic blood pressure	Per 20.75 mmHg Per 12.82 mmHg	1.34 (1.14–1.59) 1.28 (1.08–1.51)	Age, sex
Perkiomaki JS et al., 2017, Finland	Oulu Project Elucidating Risk of Atherosclerosis study	1990–1993–2009, 16.4 years follow-up	903 men and women, age 40–59 years: 91 atrial fibrillation cases	Mean ambulatory SBP	Per 5 mmHg	1.09 (1.01–1.17)	Age, sex, height, BMI, ALAT, uric acid, smoking—pack-years
Austin TR et al., 2018, USA	Jackson Heart Study	2005–2008–2012, 8.5 years follow-up	5240 men and women, mean age 55 years: 242 atrial fibrillation cases	SBP	Per 20 mmHg	1.23 (1.05–1.45)	Age, sex, height, weight, glycemic status, history of myocardial infarction, FEV_1_, eGFR, ECG PR interval, current smoking, insured, high school graduate
				DBP	Per 10 mmHg	0.99 (0.83–1.18)	
Tikhonoff V et al., 2018, Belgium, Italy, Poland, Russia, Czech Republic	FLEMENGHO and EPOGH	1985—NA, 14 years follow-up	3956 men and women, mean age 43.1 years: 143 atrial fibrillation cases	SBP	Per 17.1 mmHg	1.19 (0.99–1.43)	Age, sex, BMI, serum cholesterol, tobacco, alcohol, history of cardiovascular disease, diabetes mellitus, antihypertensive treatment
				DBP	Per 10.9 mmHg	1.10 (0.90–1.34)	
Hamada R et al., 2019, Japan	Seirei Center for Health Promotion and Preventive Medicine, Hamamatsu City	2008–2014–2015, NA	65,984 men and women, age 40–79 years: 349 atrial fibrillation cases	SBP	Per 15.4 mmHg	1.19 (1.07–1.32)	Age, sex
				DBP	Per 10.8 mmHg	1.22 (1.10–1.36)	
Kim YG et al., 2019, Korea	Korea National Health Insurance Database	NA-NA, 8.2 years follow-up	9,797,418 men and women, mean age 47 years: 196,136 atrial fibrillation cases	SBP	< 120 mmHg 120–139 140–159 ≥ 160 Per 5 mmHg	1.00 1.17 (1.16–1.19) 1.42 (1.40–1.44) 1.64 (1.61–1.68) 1.043 (1.042–1.045)	Age, sex, smoking status, alcohol, regular physical activity, low income, diabetes mellitus, dyslipidemia
				DBP	< 80 mmHg 80–89 90–99 ≥ 100 Per 5 mmHg	1.00 1.14 (1.13–1.15) 1.26 (1.24–1.28) 1.36 (1.33–1.39) 1.046 (1.044–1.048)	
Wong JA et al., 2020, Sweden	Malmo Preventive Project	1974–1992–2013, 27.6 years follow-up	32,625 men and women, mean age 45.7 years: 3277 atrial fibrillation cases (no heart failure) 1153 atrial fibrillation cases (with heart failure)	SBP, atrial fibrillation (no heart failure)	Per 10 mmHg	1.08 (1.06–1.10)	Age, sex, height, BMI, current smoking, prevalent coronary event, prevalent diabetes
				SBP, atrial fibrillation (with heart failure)	Per 10 mmHg	1.20 (1.16–1.24)	
Wong JA et al., 2020, Sweden	Malmo Diet and Cancer Study	1991–1996–2014, 17.7 years follow-up	27,695 men and women, mean age 58.2 years: 3167 atrial fibrillation cases (no heart failure) 890 atrial fibrillation cases (with heart failure)	SBP, atrial fibrillation (no heart failure)	Per 10 mmHg	1.07 (1.05–1.09)	Age, sex, height, BMI, current smoking, prevalent coronary event, prevalent diabetes
				SBP, atrial fibrillation (with heart failure)	Per 10 mmHg	1.13 (1.09–1.16)	
Igarashi Y et al., 2021, Japan	Morinomiyako Occupational Health Center, Miyagi Prefecture	2013–2016—NA, 3 years follow-up	37,562 men and women, age ≥ 40 years: 135 atrial fibrillation cases	DBP	Per 1 mmHg	1.02 (1.00–1.03)	Age, sex, waist circumference, diabetes, logγ-GTP, Minnesota codes
Espnes H et al., 2021, Norway	The Tromsø Study	1994–1995–2016, 17.6 years follow-up	24,804 men and women, age 25–97 years: 914 atrial fibrillation cases	SBP, paroxysmal/persistent atrial fibrillation, men	100 mmHg 110 120 130 140 150 160 170 180	0.81 (0.73–0.88) 0.90 (0.86–0.94) 1.00 1.11 (1.07–1.16) 1.24 (1.13–1.35) 1.38 (1.21–1.57) 1.53 (1.29–1.82) 1.70 (1.37–2.12) 1.90 (1.46–2.46)	Age, BMI, total cholesterol, current smoking, leisure-time physical activity, history of myocardial infarction, angina pectoris, stroke, diabetes mellitus
				SBP, paroxysmal/persistent atrial fibrillation, women	100 mmHg 110 120 130 140 150 160 170 180	0.56 (0.45–0.69) 0.78 (0.71–0.85) 1.00 1.22 (1.13–1.31) 1.43 (1.25–1.62) 1.62 (1.36–1.93) 1.79 (1.45–2.22) 1.95 (1.53–2.50) 2.10 (1.60–2.76)	
				SBP, permanent atrial fibrillation, men	100 mmHg 110 120 130 140 150 160 170 180	0.92 (0.83–1.01) 0.96 (0.91–1.00) 1.00 1.04 (1.00–1.10) 1.09 (0.99–1.20) 1.14 (0.99–1.31) 1.19 (0.99–1.44) 1.25 0.98–1.58) 1.30 (0.98–1.73)	
				SBP, permanent atrial fibrillation, women	100 mmHg 110 120 130 140 150 160 170 180	0.63 (0.49–0.80) 0.82 (0.74–0.91) 1.00 1.17 (1.08–1.27) 1.32 (1.15–1.53) 1.46 (1.20–1.78) 1.59 (1.25–2.02) 1.70 (1.29–2.24) 1.80 (1.33–2.44)	
Matsumoto K et al., 2021, USA	Cardiovascular Abnormalities and Brain Lesions (CABL) Study (derived from Northern Manhattan Study)	1993–2001—NA, 9.5 years follow-up	769 men and women, mean age 70.5 years: 83 atrial fibrillation cases	Office SBP	Per 10 mmHg	0.96 (0.82–1.11)	Age, sex, race, hypertension status at baseline, number of antihypertensive drugs
				Office DBP	Per 10 mmHg	1.02 (0.80–1.29)	
				Central SBP	Per 10 mmHg	1.11 (1.00–1.25)	
				Central DBP	Per 10 mmHg	1.10 (0.85–1.43)	
				24-h SBP	Per 10 mmHg	1.27 (1.09–1.49)	
				24-h diastolic blood pressure	Per 10 mmHg	1.24 (0.90–1.70)	
				Daytime SBP	Per 10 mmHg	1.24 (1.06–1.45)	
				Daytime DBP	Per 10 mmHg	1.18 (0.87–1.59)	
				Night-time SBP	Per 10 mmHg	1.24 (1.08–1.43)	
				Night-time DBP	Per 10 mmHg	1.24 (0.94–1.65)	
Lind L et al., 2021, Sweden	Uppsala Longitudinal Study of Adult Men (ULSAM)	1970–1974–2014, NA	2322 men, age 50 years: 556 atrial fibrillation cases	SBP	Per 10 mmHg	1.16 (1.05–1.26)	Triglycerides, HDL cholesterol, LDL cholesterol, BMI, diabetes, smoking
Liao LZ et al., 2021, USA	Atherosclerosis Risk in Communities Study	1987–1989–2011–2013, 24.1 years follow-up	9474 men and women, age: 1414 atrial fibrillation cases	SBP	Per 10 mmHg	1.17 (1.12–1.22)	Age, sex, race, BMI, smoking, drinking, education, sport, heart failure, coronary heart disease, diabetes, creatine, HDL-cholesterol, LDL-cholesterol, triglycerides, glucose, statin use, aspirin, anticoagulants
				DBP	Per 10 mmHg	0.90 (0.84–0.97)	
Hata J et al., 2021, Japan	The Hisayama Study	1988–2012, 24 years follow-up	2442 men and women, age 40 years: 230 atrial fibrillation cases	SBP	< 120 mmHg 120–129 130–139 140–159 160–179 ≥ 180	1.00 1.21 (0.77–1.90 1.43 (0.91–2.24) 1.49 (0.96–2.30) 1.89 (1.10–3.23) 2.12 (1.07–4.17)	Age, sex, fasting plasma glucose, serum HDL cholesterol, serum non-HDL cholesterol, serum triglycerides, BMI, waist circumference, eGFR, coronary artery disease, abnormal cardiac murmur, high R-wave amplitude on ECG, arrhythmia other than atrial fibrillation, smoking status and cigarettes per day, alcohol intake, regular exercise Age, sex
				DBP	< 80 mmHg 80–84 85–89 90–99 100–109 ≥ 110	1.00 1.07 (0.75–1.54) 2.00 (1.35–2.96) 1.14 (0.74–1.75) 2.67 (1.38–5.18) 1.98 (0.49–8.05)	
Shapkina M et al., 2022, Russia	HAPIEE study	2003–2005–2017, 12.85 years follow-up	3871 men and women, age 45–69 years: 122 atrial fibrillation cases	SBP	Per 1 mmHg	1.01 (1.00–1.02)	Age, BMI, total cholesterol, triglycerides, smoking, alcohol
Son MK et al., 2022, Korea	Korean Genome and Epidemiology Study	2001–2002–2018, 13.1 years follow-up	9049 men and women, age 40–69 years: 182 atrial fibrillation cases	SBP	< 120 mmHg 120–139 ≥ 140	1.00 1.22 (0.87–1.69) 1.54 (1.01–2.35)	Age, sex, area, obesity and central obesity, leisure-time physical activity, chronic kidney disease, cardiovascular disease, HbA1c, total cholesterol
Camen S et al., 2022, Europe	6 cohort studies: DAN-MONICA study FINRISK study Moli-sani study Northern Sweden MONICA study Scottish Heart Health Extended Cohort (SHHEC) The Tromsø Study	1982–2010, max 10 years follow-up	108,363 men and women, median age 46.0 years: 2413 atrial fibrillation cases	SBP	Per 10 mmHg	1.03 (1.01–1.05)	Age, sex, cohort, BMI, total serum cholesterol, diabetes, daily smoking, antihypertensive treatment, stroke

*ALAT* = alanin aminotransferase, *BMI* = body mass index, *DBP* = diastolic blood pressure, *ECG* = electrocardiography, *eGFR* = estimated glomerular filtration rate, *FEV1* = forced expiratory volume per 1 s, *GGTP* = gamma glutamyl transpeptidase, *HDL* = high-density lipoprotein, *LVH* = left ventricular hypertrophy, *NA* = not available, *PR* = pulse rate, *SBP* = systolic blood pressure, *ST/T* = ST and T wave ratio

Twenty three cohort studies (19 publications, 21 risk estimates) [8, 29, 33, 39, 41, 44, 46, 51, 55, 67, 69, 70, 72–74, 77, 79, 81, 82] (333,901 cases, 14,387,470 participants) were included in the analysis of diastolic blood pressure and atrial fibrillation. The summary RR was 1.06 (95% CI: 1.02–1.10, I^2^ = 92.1%, p_heterogeneity_ < 0.0001) per 10 mmHg increment (Fig. 3c). There was no evidence of publication bias with Egger's test (*p* = 0.55) or by inspection of the funnel plot (Supplementary Fig. 6). The summary RR ranged from 1.05 (95% CI: 1.00–1.10) when excluding the UK GPRD study [72] to 1.07 (95% CI: 1.03–1.11) when excluding a study at Vanderbilt University [41] (Supplementary Fig. 7). Six cohort studies [39, 55, 67–69, 79] were included in the nonlinear dose–response analysis of diastolic blood pressure and atrial fibrillation. There was evidence of nonlinearity (p_nonlinearity_ < 0.0001) with a slightly steeper increase in risk at lower levels of diastolic blood pressure than at higher levels, however, there was an increased risk from a diastolic blood pressure level of around 60 mmHg (Fig. 3d).

### Subgroup and sensitivity analyses

There were positive associations between hypertension and risk of atrial fibrillation across all subgroup analyses defined by sex, duration of follow-up, geographic location, number of cases, whether prevalent cases were excluded or not, study quality and adjustment for confounding (and in some cases potentially mediating) factors (including age, education, alcohol, smoking, BMI, physical activity, diabetes, hyperlipidemia, coronary heart disease, heart failure, valvular heart disease, left ventricular hypertrophy, and kidney disease), although the number of studies was small in some subgroups (Table 3). With meta-regression analyses there was some indication of heterogeneity between some subgroups for hypertension, with a stronger association among European studies than studies from the other geographic locations (*p* = 0.02), and a weaker association among studies with adjustment for smoking (*p* = 0.03) when compared to those without such adjustment. Further subgroup analyses by ethnicity showed summary RRs of 1.53 (95% CI: 1.29–1.80, I^2^ = 70.4%, n = 5) for Caucasians [26, 29, 45, 59] and 1.35 (95% CI: 1.16–1.59, I^2^ = 7.5%, n = 6) for African Americans [26, 29, 45, 46, 59] with no significant heterogeneity between subgroups (*p* = 0.77) (Supplementary Fig. 8).

**Table 3:**
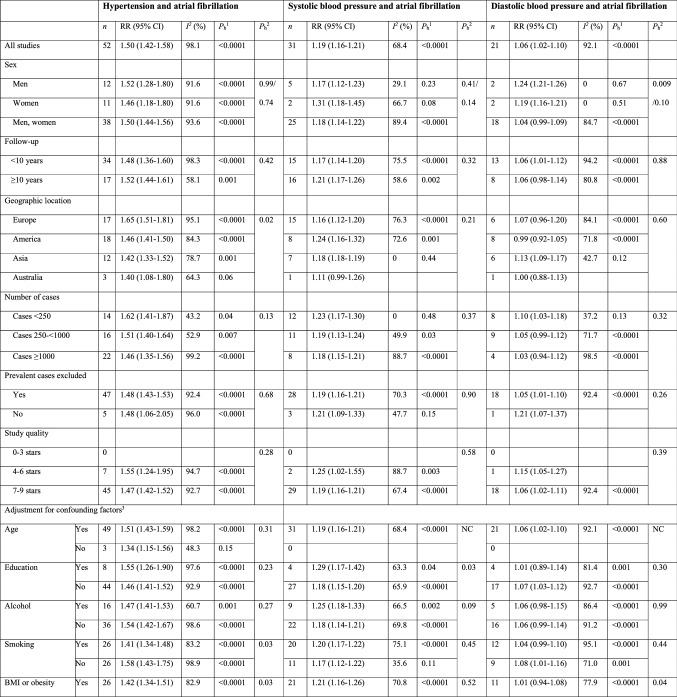

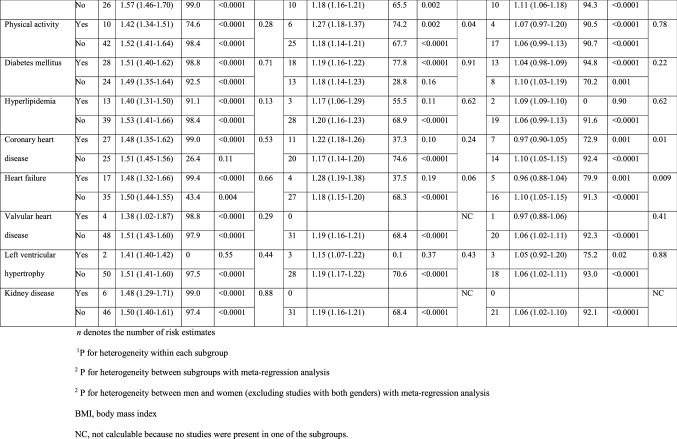
Subgroup analyses of hypertension, systolic and diastolic blood pressure and atrial fibrillation

There was evidence of heterogeneity in the subgroup analysis of systolic blood pressure and atrial fibrillation when stratified by adjustment for education (*p* = 0.03) and physical activity (*p* = 0.04) with stronger associations among studies with compared to without such adjustments, however, relatively few studies made such adjustments. For diastolic blood pressure there was heterogeneity in analyses stratified by sex (*p* = 0.009) and by adjustment for BMI or obesity (*p* = 0.04), coronary heart disease (*p* = 0.01), and heart failure (*p* = 0.009). However, the association was weaker in studies of both sexes combined than in studies among either men or women, and there was no heterogeneity when comparing men with women (and excluding studies in both sexes combined) (Table 3). There was no association in studies that adjusted for BMI or obesity, coronary heart disease, or heart failure, but a positive association in studies that did not make such adjustments (Table 3).

Mean (median) study quality scores were 7.7 (8.0) for the analysis of hypertension, 7.8 (8.0) for systolic blood pressure, and 7.8 (8.0) for diastolic blood pressure.

## Discussion

This meta-analysis of cohort studies suggests that persons with hypertension have a 50% increase in the relative risk of developing atrial fibrillation compared to persons without hypertension. There was a 19% increase in the relative risk of atrial fibrillation per 20 mmHg increase in systolic blood pressure and 6% increase in relative risk per 10 mmHg of diastolic blood pressure. Although the test for nonlinearity was significant both for systolic and diastolic blood pressure in relation to atrial fibrillation, the association with systolic blood pressure appeared to be approximately linear, while the association for diastolic blood pressure was nonlinear with a slightly steeper increase in risk at lower levels than at higher levels of diastolic blood pressure. However, there was an increased risk even within what is considered the normal blood pressure range and the lowest risk was observed at a systolic and diastolic blood pressure of 90/60 mmHg, respectively, while there was a 1.8–2.3 fold increase in risk at the high end of systolic and diastolic blood pressure around 180/110 mmHg. Positive associations were observed both in men and women, and among European, American, Asian and Australian studies, however, data from other regions are lacking. In the few studies that reported results stratified by ethnicity, there was a positive association between hypertension and atrial fibrillation among both Caucasians and African Americans. Our findings of an increased risk of atrial fibrillation with higher systolic and diastolic blood pressure are partly consistent with several recent Mendelian Randomization (MR) studies [95–97], as well as a randomized open-label trial which found a 54% reduction in risk of new-onset atrial fibrillation among participants allocated to tight vs usual blood systolic blood pressure control (target of < 130 mmHg and < 140 mmHg, respectively) [98], suggesting a possible causal relation between elevated blood pressure and atrial fibrillation. The MR studies reported stronger associations between blood pressure and atrial fibrillation when compared to the current analysis with 17–19% vs. 9% increases in risk of atrial fibrillation per 10 mmHg increase in systolic blood pressure and 25–29% vs. 6% increases in risk of atrial fibrillation per 10 mmHg increase in diastolic blood pressure, respectively. The stronger associations observed in the MR studies could be due to a stronger impact of lifelong elevated blood pressure that may be better captured in the MR studies, and potential overadjustment for intermediate risk factors in some of the observational studies. We did not observe significant differences in the association between hypertension or blood pressure and atrial fibrillation by sex, in contrast to what has been previously observed for cardiovascular disease incidence [99], but consistent with that observed for stroke [100] and cardiovascular disease mortality [99]. This suggests that for the prevention of atrial fibrillation, blood pressure lowering may be equally important among men and women.

Several biological pathways could explain an increased risk of atrial fibrillation in patients with hypertension. Elevated blood pressure increases the risk of coronary heart disease and heart failure [101], conditions that predisposes to atrial fibrillation [29, 34, 47, 48]. Hypertension induces structural remodelling of the left atrium with excessive fibroblast proliferation, and fibroblasts can switch and proliferate to myofibroblasts which have a higher profibrotic potential and also contribute to collagen accumulation [102]. Epidemiological studies have shown that elevated blood pressure predisposes to left ventricular hypertrophy [103–106], which again increases the risk of atrial fibrillation [17, 20, 29, 34, 41, 51]. It also stimulates apoptosis and inflammation of the cardiomyocytes, leading to fibrosis and left ventricular hypertrophy. Activation of the renin–angiotensin–aldosterone system and autonomic dysregulation are major factors behind these changes. Long-term hypertension can through ventricular thickening, left ventricular hypertrophy and impaired left ventricular systolic-diastolic function increase atrial pressure, ultimately leading to atrial stretch, enlargement and deterioration of atrial contraction [102]. Dysregulation of the autonomic nervous system may also contribute to the development of atrial fibrillation and it has been shown that both sympathetic and parasympathetic overactivation may trigger atrial fibrillation [102].

The present systematic review and meta-analysis has some limitations that need to be discussed. Persons with hypertension often have less healthy lifestyles than persons without hypertension, including higher BMI, less physical activity and they may be more likely to smoke. Several of the included studies adjusted for the most important confounding factors and the results persisted across most subgroup analyses, and we found little evidence of heterogeneity between these subgroups. However, we cannot exclude the possibility that residual confounding could partly explain the results. There was very high heterogeneity in the analyses of hypertension and diastolic blood pressure and moderately high heterogeneity in the analysis of systolic blood pressure and this persisted in many of the subgroup analyses, but there was lower heterogeneity in studies with a longer duration of follow-up. The heterogeneity observed appeared to be more driven by differences in the effect sizes rather than differences in the direction of the association, as all except one study found positive associations between hypertension or systolic blood pressure and atrial fibrillation. Since the studies only had one baseline assessment of hypertension status or blood pressure, regression dilution bias could have attenuated the association between hypertension or blood pressure and risk of atrial fibrillation. Some participants with high blood pressure at baseline may have undergone subsequent treatment for high blood pressure with pharmaceutical medications or lifestyle changes, which would have lowered their blood pressure, but any such effects would most likely have led to conservative estimates of the associations between hypertension and blood pressure and risk of atrial fibrillation. Some of the included studies may also have over-adjusted by including hypertension status and blood pressure in the same models, adjusting rather than stratifying for blood pressure treatment, and/or by adjusting for potentially intermediary conditions such as coronary heart disease, heart failure, valvular heart disease, and left ventricular hypertrophy in the multivariable models. Any further studies might want to adjust for potential confounders and mediators separately to evaluate the impact of both on the observed associations.

Strengths of the present meta-analysis include (1) the cohort design of the included studies (which avoids recall bias and reduces the potential for selection bias), (2) the detailed subgroup and sensitivity analyses, (3) the very large sample size with 14.3–30.5 million participants and 333,000 to 1,080,000 cases providing a more robust estimate of the association between blood pressure and hypertension and risk of atrial fibrillation than most individual studies, and (4) the detailed dose–response analyses. Our findings have important clinical and public health implications as the number of people with hypertension worldwide increased from 594 million in 1975 to 1.13 billion in 2015, mainly due to population growth and ageing, but also due to lifestyle factors [107]. This increase in the number of people with hypertension may at least have partly contributed to increased rates of atrial fibrillation. Routine screening for hypertension and lifestyle interventions to reduce blood pressure that emphasize healthy diets, physical activity, weight control and proper pharmaceutical treatment of hypertension may therefore also reduce the risk of atrial fibrillation as well as other cardiovascular complications.

## Conclusion

In conclusion, this meta-analysis suggests that people with hypertension have a 50% increase in the relative risk of developing atrial fibrillation compared to those without hypertension. Increasing systolic and diastolic blood pressure even within the normal range was associated with increased risk and at the high end was associated with a twofold increase in risk. These results strongly support a role of elevated blood pressure in the development of atrial fibrillation.

## Supplementary Information

Below is the link to the electronic supplementary material.

Supplementary file1 (PDF 559 kb)
