# Supplementary material for: Blood pressure, hypertension and the risk of atrial fibrillation: a systematic review and meta-analysis of cohort studies
Source: Eur J Epidemiol. 2023 Jan 10;38(2):145–78. doi: 10.1007/s10654-022-00914-0 (PMC9905193; doi:10.1007/s10654-022-00914-0)
Supplement: Supplementary file 1 — Supplementary file1 (PDF 559 kb) [file 10654_2022_914_MOESM1_ESM.pdf]

Supplement for

Aune D, Mahamat-Saleh Y, Kobeissi E, Feng T, Heath AK, Janszky I. Blood pressure, hypertension and the risk of atrial fibrillation: a systematic review and meta-analysis of cohort studies.

Supplementary Text. Search strategy in PubMed

("blood pressure" OR blood pressure[MeSH] OR hypertension OR hypertension[MeSH])

AND

("atrial fibrillation" OR atrial fibrillation[MeSH] or "atrial flutter" OR atrial flutter[MeSH])

AND

(“case-control” OR cohort OR cohorts OR prospective OR longitudinal OR “followed up” OR “follow-up” OR “cross-sectional” OR “hazard ratio” OR “relative risk“ OR “odds ratio” OR “odds ratios” OR prevalence OR incidence)

Search strategy in Embase

(blood pressure OR hypertension OR blood pressure/ OR hypertension/)

AND

(atrial fibrillation or atrial flutter OR atrial fibrillation/ or atrial flutter/)

AND

(case-control OR cohort OR cohorts OR prospective OR longitudinal OR followed up OR follow-up OR cross-sectional OR hazard ratio OR relative risk OR odds ratio OR prevalence OR incidence)

Supplementary Table 1. List of excluded studies and exclusion reasons

Exclusion reason	Reference number
<3 categories of exposure	(1;2)

Abstract	(3-114)
Autopsy study	(115)
Case-control study	(116-127)
Case only study	(128-152)
Cross-sectional study	(153-231)
Duplicate	(232-271)
Ecological study	(272)
Editorial	(273-281)
Elevated blood pressure, not hypertension	(282;283)
GWAS	(284)
Hospital-based study	(285)
Letter	(286-288)
Lifetime risk	(289;290)
Meta-analysis	(291;292)
Mendelian Randomization study	(293-299)
Modeling study	(300)
No risk estimates	(301-307)
Not original data	(308;309)
Not relevant data	(310)
Not relevant exposure	(311-430)
Not relevant outcome	(431-457)
Patient populations	(458-669)
Review	(670-803)
Summary for patients	(804)
Unadjusted risk estimates	(805)
Unreliable confidence intervals (asymmetrical)	(806)

Unreliable risk estimates	(807;808)
Unspecific outcome	(809)
Workshop report	(810)

Reference List

(1) Shulman E, Chudow JJ, Essien UR, Shanbhag A, Kargoli F, Romero J, Di BL, Fisher J, Krumerman A, Ferrick KJ. Relative contribution of modifiable risk factors for incident atrial fibrillation in Hispanics, African Americans and non-Hispanic Whites. Int J Cardiol 2019 Jan 15;275:89-94.

(2) Stewart S, Hart CL, Hole DJ, McMurray JJ. Population prevalence, incidence, and predictors of atrial fibrillation in the Renfrew/Paisley study. Heart 2001 Nov;86(5):516-21.

(3) Nyrnes A, Arnesen E, Njolstad I, Mathiesen EB, Wilsgaard T, Lochen ML. Diabetes and palpitations are risk factors of atrial fibrillation. The Tromso study. European Heart Journal Conference: European Society of Cardiology, ESC Congress 2009;September.

(4) Zachariah D, Kalra PR, Roberts PR, Foley RN. Risk factors for atrial fibrillation in elderly subjects without cardiovascular disease. European Heart Journal Conference: European Society of Cardiology, ESC Congress 2009;September.

(5) Tomizawa T, Otaka M, Kanashiki M, Watanabe H, Yamaguchi I. Increase in the prevalence of atrial fibrillation compared to aging in the general population: Its kinetics and risk factors. European Heart Journal Conference: European Society of Cardiology, ESC Congress 2009;September.

(6) Ariansen I, Larstorp AC, Wachtell K, Hecht OM, Ibsen H, Devereux RB, Okin PM, Kjeldsen SE, Dahlof B. Association of pulse pressure with new-onset atrial fibrillation in hypertensive patients with ECG left ventricular hypertrophy: the LIFE study. European Heart Journal Conference: European Society of Cardiology, ESC Congress 2009;September.

(7) Ishida K, Kato M, Kato Y, Yanagihara K, Kinugasa Y, Sugihara S, Hirai M, Hamada T, Igawa O, Hisatome I, Shigemasa C. Contributing factors for the incidence of paroxysmal atrial fibrillation in patients with obstructive sleep apnea. Sleep and Biological Rhythms Conference: 6th Congress of Asian Sleep Research Society, ASRS Osaka Japan Conference Publication: 2009;7(4):A19.

(8) Kolbin A, Tatarsky B, Biserova I. Burden impact of atrial fibrillation (AF BI) in Russian federation. Value in Health Conference: ISPOR 13th Annual European Congress Prague Czechia Conference Publication 2010;13(7):A347.

(9) Kokubo Y, Okamura T, Shimizu W, Watanabe M, Higashiyama A, Ono Y, Kada A, Kawanishi K, Kamakura S. Blood pressure categories and risk of incidence of atrial fibrillation in a Japanese urban cohort: the Suita study. European Heart Journal Conference: European Society of Cardiology, ESC Congress 2010;September.

(10) Alves RJ, Andrade BC, Campos RN, Nakiri K. Influence of atherosclerotic disease risk factors in the development of atrial fibrillation in the postoperative period of cardiac surgery. Atherosclerosis Supplements Conference: 78th EAS Congress Hamburg Germany Conference Publication 2010;11(2):164.

(11) Kokubo Y, Shimizu W, Watanabe M, Kada A, Kawanishi K, Kamakura S, Miyamoto Y. Impact of blood pressure and pulse rate on the risk of incident atrial fibrillation in the Suita study: An urban cohort study. Hypertension Conference: High Blood Pressure Research 2011;November.

(12) Feng P, Jinxiu L, Huilan L. Metabolic syndrome and risk of atrial fibrillation in chinese essential hypertensive patients. Heart Conference: 22nd Great Wall International Congress of Cardiology and Asia Pacific Heart Congress 2011;October.

(13) Conen D, Glynn RJ, Sandhu RK, Tedrow UB, Albert CM. Differing risk factors for incident atrial fibrillation with and without left atrial enlargement. Circulation Conference: American Heart Association's Scientific Sessions 2011.
(14) Peng F, Lin J, Liu H. Metabolic syndrome and risk of atrial fibrillation in Chinese essential hypertensive patients. International Journal of Cardiology Conference: World Hypertension League Regional Congress 2011;October.
(15) Anegawa T, Kai H, Adachi H, Fukuda K, Kajimoto H, Aoki Y, Iwamoto Y, Yasuoka S, Hirai Y, Imaizumi T. Cardiac troponin t detectable with a highly sensitive assay is associated with atrial fibrillation in the general population. Journal of Cardiac Failure Conference: 15th Annual Scientific Meeting of the Japanese Heart Failure Society, JHFS 2011;September.
(16) Grundvold I, Skretteberg PT, Liestoel K, Erikssen G, Kjeldsen SE, Arnesen H, Erikssen J, Bodegard J. High normal blood pressures predict incident atrial fibrillation in healthy middle-aged men, a 35 year follow-up study. European Heart Journal Conference: European Society of Cardiology, ESC Congress 2011;August.
(17) Kasiakogias A, Tsioufis C, Tsiachris D, Bafakis I, Almyroudi M, Giakoumis M, Kintis K, Andrikou I, Anastasopoulos I, Stefanadis C. Cardiac, vascular and renal damage as predictors for the incidence of new-onset atrial fibrillation in essential hypertensives. A Greek 6-year prospective study. European Heart Journal Conference: European Society of Cardiology, ESC Congress 2011;August.
(18) Segura J, Monivas V, Garcia-Donaire JA, Cerezo C, Ruilope LM. Relevance of atrial fibrillation in a large cohort of hypertensive patients. Journal of Clinical Hypertension Conference: 26th Annual Scientific Meeting and Exposition of the American Society of Hypertension, Inc , ASH 2011;April.
(19) Fleury G, Haddour N, Tzourio C, Ariel C. The 3 city study-COVADIS: Determinants of atrial fibrillation incidence in an elderly contemporary French cohort. Archives of Cardiovascular Diseases Supplements Conference: 22nd European Days of the French Society of Cardiology Paris France Conference Publication 2012;4(1):102.
(20) Kokubo Y, Shimizu W, Watanabe M, Kada A, Kawanishi K, Kamakura S, Kamide K, Miyamoto Y. Systolic hypertension is an independent risk of incident atrial fibrillation in a japanese urban cohort: The suita study. Journal of Hypertension Conference: 24th Meeting of the International Society of Hypertension Sydney, NSW Australia Conference Publication 2012;30 (SUPPL. 1):e9-e10.
(21) Tsiachris D, Tsioufis C, Kasiakogias A, Hatzigiannis P, Aragiannis D, Anastasopoulos I, Tzamou V. Target organ damage as predictors for the incidence of new-onset atrial fibrillation in essential hypertensives. Journal of Clinical Hypertension Conference: American Society of Hypertension, Inc 27th Annual Scientific Meeting and Exposition New York, NY United States Conference Publication 2012;14 (SUPPL.1):April.
(22) Tsiachris D, Tsioufis C, Kasiakogias A, Flesas D, Antonakis V, Kintis K, Giakoumis M, Hatzigiannis P, Katsimichas T, Stefanadis C. Predictors of new-onset atrial fibrillation in the setting of essential hypertension. Circulation Conference: American Heart Association 2012;20.
(23) Horio T, Iwashima Y, Tokudome T, Yoshihara F, Nakamura S, Akiyama M, Nishimura S, Sakaguchi M, Kawano Y. Combination therapy with renin-angiotensin system inhibitors and statins is associated with reduced incidence of new-onset atrial fibrillation in hypertensive patients. Circulation Conference: American Heart Association 2012;20.
(24) Venkatesh BA, Armstrong A, Liu C-Y, Donekal S, Yoneyama K, Wu C, Gomes AS, Hundley GW, Bluemke DA, Lima JA. Strain relaxation index predicts heart failure and atrial fibrillation over a 10 year follow-up period: The multi-ethnic study of atherosclerosis. Circulation Conference: American Heart Association 2012;20.
(25) Okin PM, Hille DA, Wachtell K, Kjeldsen SE, Dahlof B, Devereux RB. Low in-treatment hdl cholesterol strongly predicts incident atrial fibrillation: The life study. Circulation Conference: American Heart Association 2012;20.

(26) Anderson JL, Knight S, May HT, Bunch TJ, Lappe DL, Muhlestein JB. Prevalence of atrial fibrillation (AF) risk factors and lone AF in a general healthcare population. Circulation Conference: American Heart Association 2012;20.
(27) Chuang S-Y, Wu C-C, Hsu P-F, Chen RCY, Liu W-L, Pan W-H. Hyperuricemia associated with the future atrial fibrillation in normotensive elderly. Circulation Conference: American Heart Association 2012;20.
(28) Tomizawa T, Otaka M, Kanashiki M, Watanabe H, Yamaguchi I. A drinker, rather than a smoker, is at a high risk for atrial fibrillation in the general male population. European Heart Journal Conference: ESC Congress 2012;August.
(29) Kokubo Y, Shimizu W, Watanabe M, Kamakura S, Kada A, Kawanishi K, Kamide K, Miyamoto Y. Impact of blood pressure and obesity on the risk of incident atrial fibrillation in the Suita Study: An urban cohort study. European Heart Journal Conference: ESC Congress 2012;August.
(30) Huang C-C, Chan W-L, Chen Y-C, Chen T-J, Chung C-M, Huang P-H, Lin S-J, Chen J-W, Leu H-B. Risk of new-onset atrial fibrillation in patients undergoing hemodialysis. European Heart Journal Conference: ESC Congress 2012;August.
(31) Simpson J, Findlay IN, Denvir M, Murdoch DL. Anti-thrombotic therapy and atrial fibrillation in Scotland: Results of a national audit. European Heart Journal Conference: ESC Congress 2012;August.
(32) Tsiachris D, Tsioufis C, Kasiakogias A, Flessas D, Antonakis V, Kintis K, Giakoumis M, Hatzigiannis P, Katsimichas T, Stefanadis C. Prolonged ventricular depolarization as an independent predictor of new-onset atrial fibrillation in the setting of essential hypertension. European Heart Journal Conference: ESC Congress 2012;August.
(33) Margariti A, Makrygiannis SS, Rizikou D, Labakis M, Mermigkis D, Katsifa K, Laspiti I, Prekates A. Incidence and risk factors of new onset atrial fibrillation in non-cardiac intensive care unit patients. Intensive Care Medicine Conference: 26th Annual Congress of the European Society of Intensive Care Medicine, ESICM 2013;October.
(34) Milkas A, Tsioufis C, Kasiakogias A, Tsiachris D, Flessas D, Koutra E, Anastasopoulos I, Giakoumis M, Tousoulis D, Stefanadis C. Chronic kidney disease as a predictor of atrial fibrillation in treated hypertensive patients: A 4-year prospective study. Circulation Conference: American Heart Association 2013;26.
(35) Kokubo Y, Watanabe M, Kobayashi T, Kamakura S, Kawanishi K, Miyamoto Y. Maternal history of myocardial infarction and incident atrial fibrillation according to blood pressure category in a general urban population: The Suita study. Circulation Conference: American Heart Association 2013;26.
(36) Agarwal S, Shah T, Lopez F, Wong T, Gottesman R, Soliman E, Chen L, Chacko B, Klein B, Klein R, Solomon S, Heiss G, et al. Retinal microvascular abnormalities, albuminaria, and atrial fibrillation incidence: The aric study. Circulation Conference: American Heart Association 2013;26.
(37) Kokubo Y, Watanabe M, Kobayashi T, Kamakura S, Kawanishi K, Miyamoto Y. Heart murmur is a strong predictor for the incidence of atrial fibrillation in a general urban cohort: The suita study. Circulation Conference: American Heart Association 2013;26.
(38) Kokubo Y, Watanbe M, Kobayashi T, Kamakura S, Kawanishi K, Miyamoto Y. Maternal history of hypertension and incident atrial fibrillation according to blood pressure category in a general urban population: The suita study. Hypertension Conference: American Heart Association's High Blood Pressure Research 2013;September.
(39) Zhang R, Lin J-X. Association between serum uric acid and atrial fibrillation in essential hypertension patients. Heart Conference: 24th Great Wall International Congress of Cardiology, Asia Pacific Heart Congress 2013;August.
(40) Perkiomaki JS, Ukkola O, Kiviniemi A, Tulppo M, Ylitalo A, Kesaniemi YA, Huikuri HV. Heart rate variability as predictor of atrial fibrillation in middle-aged population. Journal of Electrocardiology Conference: 40th International Congress on Electrocardiology, ICE 2013;July-August.

(41) Agarwal SK, Shah T, Lopez F, Soliman EZ, Wong T, Klein R, Heiss G, Coresh J, Alonso A, Sharrett R. Retinal microvascular changes and atrial fibrillation incidence: The ARIC Study. Europace Conference: European Heart Rhythm Association, EHRA EUROPACE 2013;June.
(42) Kokubo Y, Shimizu W, Watanabe M, Kamakura S, Kada A, Kawanishi K, Miyamoto Y. Left ventricular hypertrophy as a predictor of incident atrial fibrillation and the effect of hypertension in the Suita Study: An urban cohort study. European Journal of Heart Failure Conference: Heart Failure Congress 2013;May.
(43) Reynolds MR, Hunter TD, Mollenkopf SA, Turakhia MP. Identification of patients at high risk for subsequent development of atrial fibrillation or stroke using a claims database. Value in Health Conference: ISPOR 18th Annual International Meeting New Orleans, LA United States Conference Publication 2013;16(3):A25-A26.
(44) Onuchina E, Solovyev O, Mochalova O, Onuchin S, Onuchina J. Risk factors of atrial fibrillation in patients with metabolic syndrome. Journal of Diabetes Conference: 5th International Congress on Prediabetes and the Metabolic Syndrome Early Interventions for Diabetes and Dysglycaemia Surgery in the Treatment of Obesity and Diabetes Vienna Austria Conference Publication 2013;April.
(45) White M, Tremblay-Gravel M, Roy D, Leduc H, Wyse DG, Cadrin-Tourigny J, Macle L, Dubuc M, Andrade J, Rivard L, Guerra PG, Thibault B, et al. Blood pressure and at rial fibrillation: A combined at rial fibrillation-congestive heart failure and at rial fibrillation follow-up investigation of rhythm management analysis. Journal of the American College of Cardiology Conference: 62nd Annual Scientific Session of the American College of Cardiology and i2 Summit: Innovation in Intervention, ACC 13 San Francisco, CA United States Conference Publication 2013;61.
(46) Grundvold I, Skretteberg PT, Liestoel K, Erikssen G, Engeseth K, Gjesdal K, Kjeldsen SE, Arnesen H, Erkssen J, Bodegard J. Predictors of atrial fibrillation differ in men with high vs low physical fitness. European Heart Journal Conference: European Society of Cardiology, ESC Congress 2013;August.
(47) Kintis K, Tsioufis C, Tsiachris D, Kasiakogias A, Thomopoulos C, Flessas D, Andrikou I, Bafakis I, Tousoulis D, Stefanadis C. Prognostic values of different patterns of resistant hypertension for the incidence of new-onset atrial fibrillation. European Heart Journal Conference: European Society of Cardiology, ESC Congress 2013;August.
(48) Kokubo Y, Kobayashi T, Watanbe M, Kamakura S, Kusano K, Kawanishi K, Miyamoto Y. A combination of metabolic syndrome components increased the risk of incident atrial fibrillation in a general urban cohort: The Suita study. Hypertension Conference: American Heart Association's High Blood Pressure Research 2014;November.
(49) Vermond RA, Geelhoed B, Verweij N, Tieleman RG, van der HP, Hillege HL, van Gilst WH, Van G, I, Rienstra M. Incidence of atrial fibrillation and relation with cardiovascular events, heart failure and mortality in a european community-based study-data of prevend. Circulation Conference: American Heart Association's 2014;25.
(50) Kolek MJ, Graves AJ, Bian A, Teixeira PL, Shoemaker MB, Parvez B, Xu H, Heckbert SR, Ellinor PT, Benjamin EJ, Alonso A, Denny JC, et al. External validation of a prediction model for the development of atrial fibrillation in a repository of electronic medical records. Circulation Conference: American Heart Association's 2014;25.
(51) Tsiachris D, Tsioufis C, Thomopoulos C, Dimitriadis K, Kasiakogias A, Kordalis A, Manakos K, Nikolopoulou L, Mazaraki A, Stefanadis C. Gradual predictive value of different patterns of resistant hypertension for the incidence of new-onset atrial fibrillation. European Heart Journal Conference: European Society of Cardiology, ESC Congress 2014.
(52) Marcolino MS, Marino BCA, Alkmim MBM, Ribeiro AL. Hypertension and the electrocardiogram: A study in a large primary care telecardiology dabatase in Brazil. European Heart Journal Conference: European Society of Cardiology, ESC Congress 2014.
(53) Losi M-A, Izzo R, de SG, Trimarco V, Canciello G, Manzi M-V, Contaldi C, Santoro M, De LN, Trimarco B. Incident atrial fibrillation in treated arterial hypertension: The

Campania-Salute Network (CSN). European Heart Journal Conference: European Society of Cardiology, ESC Congress 2014.
(54) Iliuta L. Predictors for new-onset post-operative atrial fibrillation in patients undergoing aortic valve replacement. European Heart Journal Conference: European Society of Cardiology, ESC Congress 2014.
(55) Lopez FL, Agarwal SK, Soliman EZ, Chen LY, Smith LG, Loehr LR, Alonso A. Trajectories of cardiovascular risk factors / outcomes and atrial fibrillation in a 25 year follow-up: The atherosclerosis risk in communities study. Circulation Conference: American Heart Association's Epidemiology and Prevention/Nutrition, Physical Activity, and Metabolism 2014.
(56) Naderi S, Wang Y, Foody J. Gender differences in atrial fibrillation hospitalizations. Circulation Conference: American Heart Association's Epidemiology and Prevention/Nutrition, Physical Activity, and Metabolism 2014.
(57) Kuwabara M, Niwa K, Hisatome I. Hyperuricemia is an independent risk factor of atrial fibrillation. Journal of the American College of Cardiology Conference: 63rd Annual Scientific Session of the American College of Cardiology and i2 Summit: Innovation in Intervention, ACC 14 Washington, DC United States Conference Publication 2014;63(12).
(58) Scantlebury D, Pearce C, Hayes S, Bailey A. Multiparity is a risk factor for atrial fibrillation. American Journal of Obstetrics and Gynecology Conference: 34th Annual Meeting of the Society for Maternal-Fetal Medicine: The Pregnancy Meeting New Orleans, LA United States Conference Publication 2014;210 (1 SUPPL.1):S267-S268.
(59) Barro BA, Giacomantonio N, Thompson K. Comparison of cardiovascular risk factors in secondary preventionaccording to daily step count. Journal of Cardiopulmonary Rehabilitation and Prevention Conference: 2015;(5):September-October.
(60) Bulbulia R, Sherliker P, Clack R, Halliday A, Peto R, Lewington S. Adiposity, systolic blood pressure, smoking and atrial fibrillation: Analyses of 2.3 million us adults attending cardiovascular screening. Circulation Conference: American Heart Association's 2015;10.
(61) Kokubo Y, Watanabe M, Higashiyama A, Nakao YM, Watanabe T, Takegami M, Kusano K, Kamakura S, Miyamoto Y. A risk score for the prediction of atrial fibrillation in the Japanese community: The Suita study. Circulation Conference: American Heart Association's 2015;10.
(62) Emdin CA, Anderson SG, Salimi-Khorshidi G, Woodward M, Rahimi K. Usual blood pressure, atrial fibrillation and vascular risk: Evidence from 4.3 million adults. European Heart Journal Conference: European Society of Cardiology, ESC Congress 2015.
(63) Polovina M, Goshev E, Kusljugic Z, Music LJ, Gjergo H, Dan GA, Paparisto V, Trendafilova E, Potpara T, Lip GYH. Sex-related differences in epidemiologic and clinical presentation of atrial fibrillation in the Balkan countries-insights from the BALKAN AF snap shot survey. European Heart Journal Conference: European Society of Cardiology, ESC Congress 2015.
(64) Yagihara N, Watanabe H, Watanabe T, Aizawa Y, Minamino T. Gender differences in risk factors for atrial fibrillation. European Journal of Preventive Cardiology Conference: EuroPrevent 2015;May.
(65) Savic L, Mrdovic I, Asanin M, Krljanac G, Lasica R. Incidence, predictors and 5-year prognostic impact of new-onset atrial fibrillation in patients with left ventricular systolic dysfunction following ST-elevation myocardial infarction. European Journal of Heart Failure Conference: Heart Failure 2015;May.
(66) Biering-Sorensen T, Mogelvang R, Schnohr P, Jensen J. The minimum left atrium volume is a superior predictor of future atrial fibrillation in a low risk general population: The Copenhagen city heart study. Journal of the American College of Cardiology Conference: 64th Annual Scientific Session of the American College of Cardiology and i2 Summit: Innovation in Intervention, ACC 15 San Diego, CA United States Conference Publication 2015;65 (10 Suppl.).

(67) Shih TL, Romer B, Al-Yafi M, Rao B, Chalazan B, McCauley M, Huang H, Galanter W, Darbar D. Race-specific impact of atrial fibrillation risk factors in blacks, hispanics, and whites in an electronic medical record. Circulation Conference: American Heart Association's 2016;(Supplement 1):November.
(68) Ogunmoroti O, Salami JA, Michos ED, Spatz ES, Allen N, Aronis K, Blankstein R, Rana JS, Virani SS, Veledar E, Blumenthal RS, Szklo M, et al. Life's simple 7 and incident atrial fibrillation: The multi-ethnic study of atherosclerosis. Circulation Conference: American Heart Association's 2016;(Supplement 1):November.
(69) Perkiomaki J, Nortamo S, Ylitalo A, Kesaniemi A, Ukkola O, Huikuri H. Ambulatory blood pressure characteristics and long-term risk for atrial fibrillation. Circulation Conference: American Heart Association's 2016;(Supplement 1):November.
(70) Kokubo Y, Imano H, Hata J, Murakoshi N, Aizawa Y, Iso H, Ninomiya T, Nakamura F, Miyamoto Y, Okamura T, Tomita H, Okumura K. The development of a risk score for incident atrial fibrillation in Japanese general population cohorts: A collaborative meta-analysis. Circulation Conference: American Heart Association's 2016;(Supplement 1):November.
(71) Lee HJ, Choi EK, Lee SR, Lim WH, Kang SH, Han KD, Cha MJ, Cho YJ, Oh IY, Oh S. Metabolic healthiness and obesity in assessing the risk of atrial fibrillation: A nationwide population-based study. European Heart Journal Conference: European Society of Cardiology, ESC Congress 2016;(Supplement 1):August.
(72) Schnabel RB, Johannsen SS, Vartiainen E, Niiranen T, Blankenberg S, Ojeda FM, Jousilahti P, Zeller T, Kuulasmaa K, Salomaa V. Epidemiology of gender differences in atrial fibrillation risk factors-the FINRISK Study. European Heart Journal Conference: European Society of Cardiology, ESC Congress 2016;(Supplement 1):August.
(73) Kokubo Y, Watanabe M, Higashiyama A, Nakao YM, Watanabe T, Takegami M, Kusano K, Kamakura S, Miyamoto Y. Combination of smoking and metabolic syndrome components increased the risk of incident atrial fibrillation: the suita study. European Heart Journal Conference: European Society of Cardiology, ESC Congress 2016;(Supplement 1):August.
(74) Kuznetsova T, Tikhonoff V, Thijs L, Stolarz-Skrzypek K, Narkiewicz K, Malyutina S, Seidlerova J, Casiglia E, Staessen JA. Long-term risk for atrial fibrillation and daytime systolic blood pressure load. European Heart Journal Conference: European Society of Cardiology, ESC Congress 2017;(Supplement 1):August.
(75) Austin T, Wiggins K, Blackshear C, Benjamin E, Sotoodehnia N, Szklo M, Correa A, Heckbert S. Incident atrial fibrillation in an African-American cohort: The jackson heart study. Circulation Conference: American Heart Association's Epidemiology and Prevention/Lifestyle and Cardiometabolic Health 2017;(Supplement 1):March.
(76) Garg P, O'neal W, Ogunsua A, Howard G, Soliman E, Cushman M. The American heart association's life's simple 7 and risk of incident atrial fibrillation: The reasons for geographic and racial differences in stroke study. Circulation Conference: American Heart Association's Epidemiology and Prevention/Lifestyle and Cardiometabolic Health 2017;(Supplement 1):March.
(77) Sharashova E.E., Wilsgaard T, Njolstad I, Mathiesen EM, Hopstock LA, Ball J, Morseth B, Lochen M-L. Long-term changes in systolic blood pressure predict risk of atrial fibrillation in a general population cohort study. European Journal of Preventive Cardiology Conference: EuroPrevent 2017;(1 Supplement 1):April.
(78) Lochen M-L, Morseth B, Hopstock LA, Ball J, Wilsgaard T, Njolstad I, Mathisen EB, Sharashova E. Temporal changes in systolic blood pressure and risk of atrial fibrillation in men and women; the Tromso Study. Norsk Epidemiologi Conference: 24th Conference of the Norwegian Epidemiological Association, NOFE 2017;(Supplement 1):October.
(79) Berge T, Ariansen I, Ihle-Hansen H, Brynildsen J, Lyngbakken MN, Christophersen IE, Myrstad M, Omland T, Steine K, Rosjo H, Smith P, Tveit A. Risk factors for atrial fibrillation at the age of 40 years: 24-year follow-up data from the Norwegian Age 40

program and the Akershus cardiac examination (ACE) 1950 Study. European Heart Journal Conference: European Society of Cardiology Congress, ESC 2018;(Supplement 1):August.
(80) Sharashova E, Wilsgaard T, Njolstad I, Mathiesen EB, Hopstock LA, Ball J, Gerdts E, Morseth B, Lochen ML. Long-term systolic blood pressure trajectories predict risk of incident atrial fibrillation in a general population cohort study. European Heart Journal Conference: European Society of Cardiology Congress, ESC 2018;(Supplement 1):August.
(81) Li YG, Pastori D, Farcomeni A, Wang YT, Guo YT, Lip GYH. Development of a risk score for incident atrial fibrillation on 471,446 chinese subjects. The C2HEST score. European Heart Journal Conference: European Society of Cardiology Congress, ESC 2018;(Supplement 1):August.
(82) Kodani E, Kaneko T, Fujii H, Nakamura H, Sasabe H, Tamura Y, Shimizu W. Predictors for New-onset Atrial Fibrillation in General Population on the Special Health Checkups of National Health Insurance (TAMA MED Project-AF). Journal of Electrocardiology Conference: ICE 2018;(e22):March-April.
(83) Shulman EH, Chudow J, Essien UR, Shanbhag A, Kargoli F, Romero J, DiBiase L, Fisher J, Pina I, Krumerman A, Ferrick KJ. Relative contribution of modifiable risk factors for atrial fibrillation in hispanics, African Americans and non-hispanic whites. Circulation Conference: 2018;(Supplement 1):November.
(84) Yang P-S, Jang E, Yu HT, Kim T-H, Uhm J-S, Pak H-N, Lee M-H, Joung B, Kim J-Y. Comparison of risk profile for new-onset atrial fibrillation between young and elderly. Circulation Conference: 2018;(Supplement 1):November.
(85) Bose A, O'Neal WT, Wu C, McClure LA, Judd SE, Howard VJ, Howard G, Soliman EZ. Sex differences in risk factors for incident atrial fibrillation: The reasons for geographic and racial differences in stroke (regards) study. Circulation Conference: 2018;(Supplement 1):November.
(86) Kim YG, Hwang KW, Kwon CH, Choi HO, Choi K-J. Metabolic syndrome and the risk of new-onset atrial fibrillation in middle-aged east asian men. Heart Rhythm Conference: 39th Annual Scientific Sessions of the Heart Rhythm Society, Heart Rhythm 2018;(5 Supplement 1):May.
(87) Konstantinidis D, Tsioufis K, Dimitriadis K, Kalos T, Liatakis I, Koutra E, Nikolopoulou L, Iliakis P, Andrikou E, Tousoulis D. Isolated systolic hypertension versus combined systolic-diastolic hypertension as predictors of atrial fibrillation: Data from a Greek 8-year-follow-up study. Journal of Hypertension Conference: 28th Scientific Meeting of the European Society of Hypertension, ESH 2018;(Supplement 1):June.
(88) Konstantinidis D, Tsioufis C, Dimitriadis K, Kasiakogias A, Liatakis I, Koutra E, Leontsinis I, Kouremeti M, Iliakis P, Vogiatzakis N, Karaminas N, Thomopoulos K, et al. Isolated systolic hypertension versus combined systolic-diastolic hypertension as predictors of atrial fibrillation: Data from a 8-year-follow-up study. European Heart Journal Conference: European Society of Cardiology Congress, ESC 2019;(Supplement 1):October.
(89) Lee S, Choi EK, Lee SR, Han K, Cha MJ, Oh S. Impact of dynamic change in the status of metabolic syndrome associated with the risk of atrial fibrillation: A nationwide population-based study. European Heart Journal Conference: European Society of Cardiology Congress, ESC 2019;(Supplement 1):October.
(90) Choi E-K, Lee SR, Han KD, Oh S. Blood pressure variability and incidence of new-onset atrial fibrillation. European Heart Journal Conference: European Society of Cardiology Congress, ESC 2019;(Supplement 1):October.
(91) Kwon CH. Impact of metabolic syndrome on the incidence of atrial fibrillation; A nationwide longitudinal cohort study in South Korea. European Heart Journal Conference: European Society of Cardiology Congress, ESC 2019;(Supplement 1):October.
(92) Ozawa A, Matsuo S, Inada K, Nagasawa H. The risk factors relating with newly development of atrial fibrillation in the non-elderly adults: Results from 25 years periodical health examination. Journal of Interventional Cardiac Electrophysiology Conference: 15th

Annual Congress of the European Cardiac Arrhythmia Society, ECAS 2019;(Supplement 1):June.
(93) Marina SM, Ryabikov A, Mazdorova E, Avdeeva E, Scherbakova L, Bobak M, Pikhart H, Malyutina S. Predictors of 13-year risk of incident atrial fibrillation in Russian population sample of middle and elderly age. European Journal of Preventive Cardiology Conference: EuroPrevent 2019;(Supplement 1):June.
(94) Sanchez JM, Dewland TA, Tseng ZH, Vittinghoff E, Nah G, Marcus GM. Epidemiology of atrial fibrillation in the american indian population. Heart Rhythm Conference: Heart Rhythm 2019;(5 Supplement):May.
(95) Li CY, Lin CP, Chien L, Lin YJ, Chen LLC, Chu PH, Chang CJ. Prediction of newly diagnosed atrial fibrillation with co-morbidity to the major adverse cardiov ascular event: A nationwide database outcome research in Taiwan. Value in Health Conference: ISPOR 2019;May.
(96) Abed H, Wong C, Brooks A, Molaee P, Nelson A, Dundon B, Leong D, Wittert G, Worthley S, Abhayaratna W, Sanders P. Periatrial fat volume is predictive of atrial fibrillation severity. Heart Lung and Circulation Conference: New Zealand Annual Scientific Meeting of the Cardiac Society of Australia and New Zealand Adelaide, SA Australia Conference Publication 2019;(SUPPL. 2):2010.
(97) Choi YY, Choi J-I, Choi HY, Kim YG, Boo KY, Kim DY, Lee K-N, Shim J, Kim JS, Kim Y-H. Impact of the duration and degree of hypertension and body weight on new-onset atrial fibrillation: A nationwide population-based study. Journal of Arrhythmia Conference: 12th Asia Pacific Heart Rhythm Society Session, APHRS 2019;(Supplement 1):December.
(98) Kornej J, Henger S, Burkhardt R, Thiele H, Thiery J, Scholz M. Association between the presence and extent of coronary artery disease and atrial fibrillation: Insights from the life-heart study. Circulation Conference: American Heart Association Scientific Sessions, AHA 2019;(Supplement 1):2019.
(99) Igarashi Y, Nochioka K, Tomomasa M, Tamai T, Okochi S, Irokawa T, Ogawa H, Hayashi H, Fujihashi T, Yamanaka S, Aoyanagi H, Kasahara S, et al. Risk prediction models for new-onset atrial fibrillation among male workers: Usefulness of the minnesota code electrocardiography classification system. Circulation Conference: American Heart Association Scientific Sessions, AHA 2019;(Supplement 1):2019.
(100) Grundvold I, Skretteberg T, Liesto K, Kjeldsen SE, Arnesen H, Erikssen J, Bodegard J. Systolic blood pressure above 128 mmhg predicts atrial fibrillation in apparently healthy men; A 35-year follow-up study. Journal of Hypertension Conference: 2020;June.
(101) Belu E, Musetescu R, Ionescu DD, Musetescu AE, Belu V, Cosulschi M, Gusti S, Toader D. Could early diagnosis of metabolic syndrome lower the incidence of atrial fibrillation in hypertensive patients? Journal of Hypertension Conference: 2020;June.
(102) Pellegrini C, Kizer J, Buzkova P, Matthan N, Lichtenstein A, Siscovick D, Ix J, Heckbert SR, Mukamal K, Djousse L. Associations of individual plasma non-esterified fatty acids with incident atrial fibrillation in older adults: The cardiovascular health study. Journal of the American College of Cardiology Conference: ACC 20 - World Congress of Cardiology United States 2020;75(11):404.
(103) Labbaoui M, Doyen D, Squara F, Ferrari E. Incidence, clinical features, and long-term outcomes of new-onset atrial fibrillation in the intensive care unit: A prospective observational study. Archives of Cardiovascular Diseases Supplements Conference: 30es JOURNEES EUROPEENNES de la SOCIETE FRANCAISE de CARDIOLOGIE France 2020;12(1):117.
(104) Espnes H, Lochen ML, Wilsgaard T, Ball J, Njolstad I, Mathiesen EB, Sharashova E. Risk factors associated with specific types of atrial fibrillation in men and women in a general population cohort study. European Journal of Preventive Cardiology Conference: 11th European Association for Palliative Care Congress, EAPC 2020;(1 SUPPL4):July.
(105) Lee SR, Park CS, Choi EK, Ahn HJ, Han KD, Oh S, Lip GYH. Hypertension burden and the risk of new-onset atrial fibrillation: A nationwide population- based study. Europace

Conference: Annual Congress of the European Heart Rhythm Association, EHRA 2021;(SUPPL 3):May.
(106) Lee SR, Han KD, Choi EK, Ahn HJ, Oh S, Lip GYH. Risk of atrial fibrillation in young adults aged 20 to 39 years with isolated diastolic, isolated systolic, and systolic-diastolic hypertension: A nationwide population-based study. European Heart Journal Conference: European Society of Cardiology Congress, ESC 2021;(SUPPL 1):October.
(107) Camen S, Csengeri D, Geelhoed B, Gianfagna F, Soderberg S, Kee F, Blankenberg S, Lochen ML, Iacoviello L, Tunstall-Pedoe H, Joergensen T, Salomaa V, et al. Risk factors, subsequent disease onset and prognostic impact of myocardial infarction and atrial fibrillation. European Heart Journal Conference: European Society of Cardiology Congress, ESC 2021;(SUPPL 1):October.
(108) Tomizawa T, Otaka M, Yamashita R, Kanashiki M, Watanabe H, Yamaguchi I. Sex- and age-stratified 20-year changes in the prevalence of atrial fibrillation in general population. European Heart Journal Conference: European Society of Cardiology, ESC Congress 2014.
(109) Healey JS, Connolly SJ, Manja V, Liu Y, Simek KD, Quinn R, Sandhu RK, Ivers N, Gladstone DJ. Sub-clinical atrial fibrillation in elderly primary care patients without clinical atrial fibrillation. Circulation Conference: American Heart Association's 2015;10.
(110) Lee J, Shrout T, Benjamin EJ, Ramachandran VS, Xanthakis V. Association of blood pressure responses to submaximal exercise with incident atrial fibrillation: The framingham offspring study. Circulation Conference: American Heart Association's Epidemiology and Prevention/Lifestyle and Cardiometabolic Health 2021;(SUPPL 1):May.
(111) Allan V, Honarbakhsh S, Casas JP, Wallace J, Hunter RJ, Schilling RJ, Perel P, Morely KI, Banerjee A, Hemingway H. Are cardiovascular risk factors associated with the incidence of atrial fibrillation? A systematic review and field synopsis of 23 factors in 32 initially healthy cohorts of 20 million participants. European Heart Journal Conference: European Society of Cardiology, ESC Congress 2016;(Supplement 1):August.
(112) Petrella RJ, Sauriol L. Atriall fibrillation epidemiology and management in primary care. Value in Health Conference: 15th Annual International Meeting of the International Society for Pharmacoeconomics and Outcomes Research, ISPOR 2010;May.
(113) Fauchier L, Pelade C, Collignon C, Nicolle E. How to identify a risk for subsequent atrial fibrillation and higher risk of stroke in patients with hospitalization related to a cardiac cause? Archives of Cardiovascular Diseases Supplements Conference: 25es Journees Europeennes de la Societe Francaise de Cardiologie Paris France Conference Publication 2015;7(1):59.
(114) Nakamura S, Adachi H, Enomoto M, Fukami A, Nohara Y, Nakao E, Fukumoto Y. The trend of atrial fibrillation in the elderly men for 30 years in a japanese general population. Journal of Hypertension Conference: 25th European Meeting on Hypertension and Cardiovascular Protection, ESH 2015;June.
(115) Sugiura M, Ohkawa S, Ueyama C. A clinicopathological study on the atrial fibrillation and atrial volume in the aged. [Japanese]. Japanese Journal of Geriatrics 1981;18(5):349-53.
(116) Barriales A, V, Moris De La TC, Sanchez P, I, Barriales VR, Rubin LJ, de la Hera Galarza JM, Vara MJ, Hevia NS, Cortina LA. The etiology and associated risk factors in a sample of 300 patients with atrial fibrillation. [Spanish]. Revista espanola de cardiologia 1999;52(6):403-14.
(117) Thomas MC, Dublin S, Kaplan RC, Glazer NL, Lumley T, Longstreth WT, Jr., Smith NL, Psaty BM, Siscovick DS, Heckbert SR. Blood pressure control and risk of incident atrial fibrillation. Am J Hypertens 2008 Oct;21(10):1111-6.
(118) Bohm M, Thoenes M, Neuberger H-R, Graber S, Reil J-C, Bramlage P, Volpe M. Atrial fibrillation and heart rate independently correlate to microalbuminuria in hypertensive patients. European Heart Journal 2009;30(11):1364-71.
(119) Calvo N, Ramos P, Montserrat S, Guasch E, Coll-Vinent B, Domenech M, Bisbal F, Hevia S, Vidorreta S, Borras R, Falces C, Embid C, et al. Emerging risk factors and the dose-

response relationship between physical activity and lone atrial fibrillation: A prospective case-control study. Europace 2015;18(1):57-63.
(120) Chamberlain AM, Byrne MC, Alonso A, Gersh BJ, Manemann SM, Killian JM, Weston SA, Roger VL. Comorbidity burden in atrial fibrillation: A population-based case-control study. Circulation Conference: American Heart Association's Epidemiology and Prevention/Lifestyle and Cardiometabolic Health 2015;10.
(121) Arsava EM, Yetim E, Canpolat U, Ozer N, Aytemir K, Topcuoglu MA. Short lasting runs (<30 sec) of atrial fibrillation are more commonly observed in ischemic stroke patients in comparison to controls. Stroke Conference: American Heart Association/American Stroke Association 2016;February.
(122) Stojanovska J, Kazerooni EA, Sinno M, Gross BH, Watcharotone K, Patel S, Jacobson JA, Oral H. Increased epicardial fat is independently associated with the presence and chronicity of atrial fibrillation and radiofrequency ablation outcome. European Radiology 2015;25(8):2298-309.
(123) Novello P, Ajmar G, Bianchini D, Bo GP, Cammarata S, Firpo MP, Parodi CI, Patrone A, Pizio N, Poeta MG. Ischemic stroke and atrial fibrillation. A clinical study. Italian journal of neurological sciences 1993;14(7):571-6.
(124) Braga B, Poyares D, Cintra F, Guilleminault C, Cirenza C, Horbach S, Macedo D, Silva R, Tufik S, De Paola AAV. Sleep-disordered breathing and chronic atrial fibrillation. Sleep Medicine 2009;10(2):212-6.
(125) Yu-Feng H, Yeh H-I, Shih C-M, Chen S-A. Is CD36 a key connection between atrial fibrillation and metabolic syndrome? Heart Rhythm Conference: 30th Annual Scientific Sessions of the Heart Rhythm Society, Heart Rhythm 2009;May.
(126) Dublin S, Glazer NL, Smith NL, Psaty BM, Lumley T, Wiggins KL, Page RL, Heckbert SR. Diabetes mellitus, glycemic control, and risk of atrial fibrillation. Journal of General Internal Medicine 2010;25(8):853-8.
(127) Hodgkinson JA, Taylor CJ, Hobbs FDR. Predictors of incident atrial fibrillation and influence of medications: A retrospective case-control study. British Journal of General Practice 2011;61(587):e353-e361.
(128) Qi W-H. Retrospective investigation of hospitalized patients with atrial fibrillation in Mainland China. Chinese Medical Journal 2004;117(12):1763-7.
(129) Wen-Hang QI. Retrospective investigation of hospitalised patients with atrial fibrillation in mainland China. International Journal of Cardiology 2005;105(3):283-7.
(130) Mashat AA, Subki AH, Bakhaider MA, Baabdullah WM, Walid JB, Alobudi AH, Fakeeh MM, Algethmi AJ, Alhejily WA. Atrial fibrillation: risk factors and comorbidities in a tertiary center in Jeddah, Saudi Arabia. Int J Gen Med 2019;12:71-7.
(131) Piccini JP, Hammill BG, Sinner MF, Jensen PN, Hernandez AF, Heckbert SR, Benjamin EJ, Curtis LH. Incidence and prevalence of atrial fibrillation and associated mortality among Medicare beneficiaries, 1993-2007. Circ Cardiovasc Qual Outcomes 2012 Jan;5(1):85-93.
(132) Tremblay-Gravel M, White M, Roy D, Leduc H, Wyse DG, Cadrin-Tourigny J, Shohoudi A, Macle L, Dubuc M, Andrade J, Rivard L, Guerra PG, et al. Blood Pressure and Atrial Fibrillation: A Combined AF-CHF and AFFIRM Analysis. J Cardiovasc Electrophysiol 2015 May;26(5):509-14.
(133) Lok N-S. Presentation and management of patients admitted with atrial fibrillation: A review of 291 cases in a regional hospital. International Journal of Cardiology 1995;48(3):271-8.
(134) Boriani G, Capucci A, Bruni V, Spedicato L, Biffi M, Sabbatani P, Frabetti L, Branzi A, Magnani B. Idiopathic atrial fibrillation: clinical-instrumental characterization and thromboembolic risk. [Italian]. Cardiologia (Rome, Italy) 1995;40(1):23-30.
(135) Shatoor AS, Ahmed ME, Said MA, Shabbir K, Cheema A, Kardash MO. Patterns of atrial fibrillation at a regional hospital in Saudi Arabia. Ethnicity & disease 1998;8(3):360-6.

(136) Boriani G, Gallina M, Biffi M, Bronzetti G, Manetti R, De SN, Branzi A, Magnani B. Clinical pattern and complications of nonvalvular atrial fibrillation in elderly and nonelderly patients. Journal of Cardiovascular Diagnosis and Procedures 1998;15(1):31-6.
(137) Mareedu RK, Abdalrahman IB, Dharmashankar KC, Granada JF, Chyou P-H, Sharma PP, Smith PN, Hayes JJ, Greenlee RT, Vidaillet H. Atrial flutter versus atrial fibrillation in a general population: Differences in comorbidities associated with their respective onset. Clinical Medicine and Research 2010;8(1):1-6.
(138) Naderi S, Wang Y, Miller AL, Rodriguez F, Chung MK, Radford MJ, Foody JM. The impact of age on the epidemiology of atrial fibrillation hospitalizations. Am J Med 2014 Feb;127(2):158-7.
(139) Lane DA, Skjoth F, Lip GYH, Larsen TB, Kotecha D. Temporal Trends in Incidence, Prevalence, and Mortality of Atrial Fibrillation in Primary Care. J Am Heart Assoc 2017 Apr 28;6(5).
(140) Letsas KP, Weber R, Burkle G, Mihas CC, Minners J, Kalusche D, Arentz T. Pre-ablative predictors of atrial fibrillation recurrence following pulmonary vein isolation: The potential role of inflammation. Europace 2009;11(2):158-63.
(141) Babar T, Mekuria S, Malik P, Chung MK. Obesity and recurrence of atrial fibrillation after cardioversion. Journal of the American College of Cardiology Conference: American College of Cardiology 58th Annual Scientific Session and i2 Summit: Innovation in Intervention Orlando, FL United States Conference Publication 2009;53(10):A113.
(142) Hussein AA, Hawi R, Banna M, Batal O, Williams-Andrews M, Sherman M, Tchou P, Martin D, Kanj M, Saliba W, Wazni O. Very late recurrence of atrial fibrillation after pulmonary vein antrum isolation. Heart Rhythm Conference: 30th Annual Scientific Sessions of the Heart Rhythm Society, Heart Rhythm 2009;May.
(143) Blum S, Aeschbacher S, Meyre P, Zwimpfer L, Reichlin T, Beer JH, Ammann P, Auricchio A, Kobza R, Erne P, Moschovitis G, Di VM, et al. Incidence and Predictors of Atrial Fibrillation Progression. J Am Heart Assoc 2019 Oct 15;8(20):e012554.
(144) Kerr C, Boone J, Connolly S, Greene M, Klein G, Sheldon R, Talajic M. Follow-up of atrial fibrillation: The initial experience of the Canadian registry of atrial fibrillation. European Heart Journal 1996;7 (7 SUPPL.C):48-51.
(145) Gurevitz OT, Varadachari CJ, Ammash NM, Malouf JF, Rosales AG, Herges RM, Bruce CJ, Somers VK, Hammill SC, Gersh BJ, Friedman PA. The effect of patient sex on recurrence of atrial fibrillation following successful direct current cardioversion. American Heart Journal 2006;152(1):155.e9-155.e13.
(146) Ma X, Zhang X, Guo W. Factors to predict recurrence of atrial fibrillation in patients with hypertension. Clinical Cardiology 2009;32(5):264-8.
(147) Rigopoulos AG, Kalogeropoulos AS, Rizos I, Triantafyllis A, Sakadakis EA, Voltyrakis E, Papathanasiou S, Dragomanovits S, Kremastinos DT. Dynamic evolution of high sensitivity C reactive protein and interleukin-6 serum levels relates with cardioversion and recurrence of recent onset atrial fibrillation in patients with hypertension. Journal of Clinical Hypertension Conference: 24th Annual Scientific Meeting and Exposition of the American Society of Hypertension, ASH San Francisco, CA United States Conference Publication 2009;11 (4 SUPPL.1):A168.
(148) Kowalik I, Dabrowski R, Borowiec A, Smolis-Bak E, Szwed H. Effective combination treatment of hypertension as a part up-stream therapy in prevention of atrial fibrillation episodes recurrence: Prospective, 1-year evaluation. European Heart Journal Conference: European Society of Cardiology, ESC Congress 2010;September.
(149) Santoro F, Di BL, Trivedi C, Burkhardt JD, Paoletti PA, Sanchez J, Horton R, Mohanty P, Mohanty S, Bai R, Santangeli P, Lakkireddy D, et al. Impact of uncontrolled hypertension on atrial fibrillation ablation outcome. JACC: Clinical Electrophysiology 2015;1(3):164-73.
(150) Weng W, Bernick J, Wells G, Tardif J, Tang A, Sapp J, Gray C, Gardner M, Healey J, Parkash R. Predictors of atrial fibrillation after catheter ablation in a hypertensive population: A sub-study of SMAC-AF. Canadian Journal of Cardiology Conference: 70th

Annual Meeting of the Canadian Cardiovascular Society Canada 2017;33 (10 Supplement 1):-S150.
(151) Badila E, Frunza SA, Tirziu C, Dumitrache L, Bartos D, Dorobantu M. The benefit of therapy using reninangiotensin system blockers in preventing recurrent atrial fibrillation in high risk hypertensive patients. Journal of Hypertension Conference: 2020;June.
(152) Aberg H. Atrial fibrillation. A review of 463 cases from Philadelphia General Hospital from 1955 to 1965. Acta Medica Scandinavica 1968;184(5):425-31.
(153) Lip GYH. A survey of atrial fibrillation in general practice: The west Birmingham atrial fibrillation project. British Journal of General Practice 1997;47(418):285-9.
(154) Hurley V, Ireson R, Fletcher K, Lip GY, Hobbs FR, Mant J. A cross-sectional study of hypertension in an elderly population (75 years and over) with atrial fibrillation: Secondary analysis of data from the Birmingham Atrial Fibrillation in the Aged (BAFTA) randomised controlled trial. International Journal of Cardiology 2007;117(2):152-6.
(155) Haq U, Lip GYH. A prospective survey of acute hospital admissions with atrial fibrillation in Karachi, Pakistan. Journal of the Royal College of Physicians of Edinburgh 2009;39(3).
(156) Liu Y, Yang YM, Zhu J. Prevalence, management and stroke prevention for non-vavular atrial fibrillation patients with hypertension. Cardiology Conference: International Heart Forum 2009;November.
(157) Long MJ, Jiang CQ, Lam TH, Xu L, Zhang WS, Lin JM, Ou JP, Cheng KK. Atrial fibrillation and obesity among older Chinese: The Guangzhou Biobank Cohort Study. International Journal of Cardiology 2011;148(1):48-52.
(158) Tocci G, Cicero AF, Salvetti M, Francia P, Ferrucci A, Borghi C, Volpe M. Attitudes and preferences for the clinical management of hypertension and hypertension-related cardiac disease in general practice: Results of the Italian Hypertension and Heart Survey. Journal of Human Hypertension 2015;29(7):409-16.
(159) Joon HJ. Prevalence of and risk factors for atrial fibrillation in Korean adults older than 40 years. Journal of Korean Medical Science 2020;(1):February.
(160) Ali AS, Fenn NM, Zarowitz BJ, Niemyski P, Vitarelli A, Gheorghiade M. Epidemiology of atrial fibrillation in patients hospitalized in a large hospital. Panminerva Med 1993 Dec;35(4):209-13.
(161) Fiandra U, Bo M, Fonte G, Casoli G, Ferrario E. Associations between arterial hypertension and other cardiovascular risk factors in subjects over 65 years of age. [Italian]. Recenti progressi in medicina 1995;86(4):147-54.
(162) Wandell PE. A survey of subjects with present or previous atrial fibrillation in a Swedish community. Scand J Prim Health Care 2001 Mar;19(1):20-4.
(163) Ahmed ME, Shatoor AS, Board A. Resting ECG abnormalities among asymptomatic Arab men and comparison with other ethnic populations. Ethnicity & disease 2001;11(3):446-53.
(164) Friberg J, Scharling H, Gadsboll N, Jensen GB. Sex-specific increase in the prevalence of atrial fibrillation (The Copenhagen City Heart Study). Am J Cardiol 2003 Dec 15;92(12):1419-23.
(165) Freestone B, Rajaratnam R, Hussain N, Lip GY. Admissions with atrial fibrillation in a multiracial population in Kuala Lumpur, Malaysia. Int J Cardiol 2003 Oct;91(2-3):233-8.
(166) Wattigney WA, Mensah GA, Croft JB. Increasing trends in hospitalization for atrial fibrillation in the United States, 1985 through 1999 implications for primary prevention. Circulation 2003;108(6):711-6.
(167) Ostgren CJ, Merlo J, Rastam L, Lindblad U. Atrial fibrillation and its association with type 2 diabetes and hypertension in a Swedish community. Diabetes Obes Metab 2004 Sep;6(5):367-74.
(168) Rastas S, Mattila K, Verkkoniemi A, Niinisto L, Juva K, Sulkava R, Lansimies E. Association of apolipoprotein E genotypes, blood pressure, blood lipids and ECG abnormalities in a general population aged 85+. BMC Geriatr 2004 Mar 29;4:1.
(169) Jeong JH. Prevalence of and risk factors for atrial fibrillation in Korean adults older than 40 years. J Korean Med Sci 2005 Feb;20(1):26-30.

(170) Guize L, Thomas F, Bean K, Benetos A, Pannier B. Atrial fibrillation: prevalence, risk factors and mortality in a large French population with 15 years of follow-up. [French]. Bulletin de l'Academie nationale de medecine 2007;191(4-5):791-803.
(171) Imanaka S. [Epidemiological study on atrial fibrillation among aged members of a Japanese community]. Nihon Ronen Igakkai Zasshi 2008 Nov;45(6):634-9.
(172) Iguchi Y, Kimura K, Aoki J, Kobayashi K, Terasawa Y, Sakai K, Shibazaki K. Prevalence of atrial fibrillation in community-dwelling Japanese aged 40 years or older in Japan: analysis of 41,436 non-employee residents in Kurashiki-city. Circ J 2008 Jun;72(6):909-13.
(173) Yap KB, Ng TP, Ong HY. Low prevalence of atrial fibrillation in community-dwelling Chinese aged 55 years or older in Singapore: a population-based study. J Electrocardiol 2008 Mar;41(2):94-8.
(174) Borzecki AM, Bridgers DK, Liebschutz JM, Kader B, Kazis LE, Berlowitz DR. Racial differences in the prevalence of atrial fibrillation among males. Journal of the National Medical Association 2008;100(2):237-45.
(175) Alegret JM, Vinolas X, Sagrista J, Hernandez-Madrid A, Berruezo A, Moya A, Martinez Sande JL, Pastor A. Clinical characteristics of patients with persistent atrial fibrillation referred for cardioversion: Spanish cardioversion registry (REVERSE). [Spanish]. Revista espanola de cardiologia 2008;61(6):630-4.
(176) Naccarelli GV, Varker H, Lin J, Schulman KL. Increasing Prevalence of Atrial Fibrillation and Flutter in the United States. American Journal of Cardiology 2009;104(11):1534-9.
(177) Naccarelli GV, Varker H, Lin J, Schulman KL. Increasing prevalence of atrial fibrillation and flutter in the United States: Is it just due to the population aging? Journal of the American College of Cardiology Conference: American College of Cardiology 58th Annual Scientific Session and i2 Summit: Innovation in Intervention Orlando, FL United States Conference Publication 2009;53(10):A104-A105.
(178) Bonhorst D, Mendes M, Adragao P, De SJ, Primo J, Leiria E, Rocha P. Prevalence of atrial fibrillation in the Portuguese population aged 40 and over: the FAMA study. Rev Port Cardiol 2010 Mar;29(3):331-50.
(179) Liu Y, Liu H, Dong L, Chen J, Guo J. Prevalence of atrial fibrillation in hospitalized patients over 40 years old: ten-year data from the People's Hospital of Peking University. Acta Cardiol 2010 Apr;65(2):221-4.
(180) Ninios I, Bogossian H, Zarse M, Lazaridou F, Dimitriadis K, Ninios V, Lemke B, Louridas G. Prevalence, clinical correlates and treatment of permanent atrial fibrillation among the elderly: Insights from the first prospective population-based study in rural Greece. Journal of Thrombosis and Thrombolysis 30 (1) (pp 90-96), 2010;30(1):90-6.
(181) Cohen A, Dallongeville J, Durand-Zaleski I, Bouee S, Le HJY. Characteristics and management of outpatients with history of or current atrial fibrillation: The observational French EPHA study. Archives of Cardiovascular Diseases 2010;103(6-7):376-87.
(182) Lahiri MK, Fang K, Lamerato L, Khan A, Schuger CD. Racial differences in risk for atrial fibrillation only occurs in older patients. Heart Rhythm Conference: 31st Annual Scientific Sessions of the Heart Rhythm Society, Heart Rhythm 2010;May.
(183) Wong CX, Cheng Y, Lau DH, Leong DP, Brooks AG, Shipp NJ, Lim HS, Abed HS, Alasady M, Sanders P. Prevalence of atrial fibrillation in indigenous Australians. European Heart Journal Conference: European Society of Cardiology, ESC Congress 2010;September.
(184) Garadah T, Gabani S, Alawi MA, bu-Taleb A. Prevalence and Predisposing Factors of Atrial Fibrillation in a Multi-Ethnic Society: The Impact of Racial Differences in Bahrain. Open J Cardiovasc Surg 2011;4:9-16.
(185) Schnabel RB, Wilde S, Wild PS, Munzel T, Blankenberg S. Atrial fibrillation: its prevalence and risk factor profile in the German general population. Dtsch Arztebl Int 2012 Apr;109(16):293-9.

(186) Ntaios G, Manios E, Synetou M, Savvari P, Vemmou A, Koromboki E, Saliaris M, Blanas K, Vemmos K. Prevalence of atrial fibrillation in Greece: the Arcadia Rural Study on Atrial Fibrillation. Acta Cardiol 2012 Feb;67(1):65-9.
(187) Wong JS, B R, A F, I R, Fong AY. Prevalence of asymptomatic atrial fibrillation in malaysian patients with hypertension. Med J Malaysia 2013 Apr;68(2):141-3.
(188) Frewen J, Finucane C, Cronin H, Rice C, Kearney PM, Harbison J, Kenny RA. Factors that influence awareness and treatment of atrial fibrillation in older adults. QJM 2013;106(5):415-24.
(189) Baena-Diez JM, Grau M, Fores R, Fernandez-Berges D, Elosua R, Sorribes M, Felix-Redondo FJ, Segura A, Rigo F, Cabrera de LA, Sanz H, Marrugat J, et al. Prevalence of atrial fibrillation and its associated factors in Spain: An analysis of 6 population-based studies. DARIOS Study. Rev Clin Esp 2014 Dec;214(9):505-12.
(190) Wong CX, Brooks AG, Cheng Y-H, Lau DH, Rangnekar G, Roberts-Thomson KC, Kalman JM, Brown A, Sanders P. Atrial fibrillation in Indigenous and non-Indigenous Australians: A cross-sectional study. BMJ Open 2014;4(10):e006242.
(191) Chei CL, Raman P, Ching CK, Yin ZX, Shi XM, Zeng Y, Matchar DB. Prevalence and Risk Factors of Atrial Fibrillation in Chinese Elderly: Results from the Chinese Longitudinal Healthy Longevity Survey. Chin Med J (Engl) 2015 Sep 20;128(18):2426-32.
(192) Marcolino MS, Palhares DM, Benjamin EJ, Ribeiro AL. Atrial fibrillation: prevalence in a large database of primary care patients in Brazil. Europace 2015 Dec;17(12):1787-90.
(193) Sun GZ, Guo L, Wang XZ, Song HJ, Li Z, Wang J, Sun YX. Prevalence of atrial fibrillation and its risk factors in rural China: a cross-sectional study. Int J Cardiol 2015 Mar 1;182:13-7.
(194) Gomes E, Campos R, Morais R, Fernandes M. FATA study: Prevalence of atrial fibrillation and antithrombotic therapy in primary health care in a northern city of Portugal. [Portuguese]. Acta Medica Portuguesa 2015;28(1):35-43.
(195) Berti D, Moors E, Moons P, Heidbuchel H. Prevalence and antithrombotic management of atrial fibrillation in hospitalised patients. Heart 2015;101(11):884-93.
(196) Ghazi L, Oparil S, Calhoun D, Dudenbostel T. Characteristics of young and old patients with resistant hypertension in a large, diverse cohort. Journal of the American Society of Hypertension Conference: 30th Annual Scientific Meeting of the American Society of Hypertension New York, NY United States Conference Publication 2015;9 (4 SUPPL.1):e10-e11.
(197) Bohlender J, Nussberger J, Bohlender B. Are hospitalised patients aged 90 years and over treated well for hypertension? Lessons from a prospective survey. [French, English]. Annales de Cardiologie et d'Angeiologie Conference: 9th International Meeting of the French Society of Hypertension Paris France Conference Publication 2015;64 (SUPPL.1):S7.
(198) Wong JA, Sandhu RK, Conen D, Albert CM. Impact of new guidelines on atrial fibrillation pattern classification: The women's health study. Circulation Conference: American Heart Association's 2015;10.
(199) Mansencal N, Bagate F, Arslan M, Siam-Tsieu V, Deblaise J, Mahmoud RE, Dubourg O. Prevalence of atrial fibrillation during dobutamine stress echocardiography. Archives of Cardiovascular Diseases Supplements Conference: 25es Journees Europeennes de la Societe Francaise de Cardiologie Paris France Conference Publication 2015;7(1):39.
(200) Sud R, Goel R, Gupta R, Kachru R, Kaul U. Experience with atrial fibrillation at a tertiary care centre. Indian Heart Journal Conference: 67th Annual Conference of Cardiological Society of India, CSI 2015;December.
(201) Sun G, Guo L, Wang X, Song H, Li Z, Wang J, Sun Y. Prevalence of atrial fibrillation and its risk factors in rural China: A cross-sectional study. Journal of the American College of Cardiology Conference: 26th Great Wall International Congress of Cardiology, Asia Pacific Heart Congress 2015;20.

(202) Marcolino MS, Chazard E, Palhares D, Dumesnil C, Caron A, Ficheur G, Beuscart R, Alkmim MB, Macfarlane P, Ribeiro AL. One million electrocardiograms of primary care patients. European Heart Journal Conference: European Society of Cardiology, ESC Congress 2015;01.
(203) Torbova S, Anev E, Yotov Y, Tisheva S, Ramshev K, Ivanova V, Kolev E. Arterial hypertension and asymptomatic atrial fibrillation in young, middle-aged and elderly groups of bulgarian urban population. A cross-sectional study. Journal of Hypertension Conference: 25th European Meeting on Hypertension and Cardiovascular Protection, ESH 2015;June.
(204) Dawood F, Rodriguez C, Smoller SW, Alonso A, Benjamin EJ, Heckbert S, Soliman E, Daviglus ML, Swett K, Schneiderman N. Atrial fibrillation prevalence and clinical correlates among diverse hispanic/latinos: The hispanic community health study / study of latinos (HCHS-SOL). Circulation Conference: American Heart Association's Epidemiology and Prevention/Lifestyle and Cardiometabolic Health 2015;10.
(205) Bhatt H, Gamboa CM, Safford MM, Soliman EZ, Glasser SP. Is there an association between the prevalence of atrial fibrillation and severity and control of hypertension? The REasons for Geographic And Racial Differences in Stroke study. J Am Soc Hypertens 2016 Jul;10(7):578-86.
(206) Modesti PA, Colella A, Zhao D. Atrial fibrillation in first generation Chinese migrants living in Europe: A proof of concept study. Int J Cardiol 2016 Jul 15;215:269-72.
(207) Bhatt H, Gamboa C, Soliman E, Safford M, Glasser S. Is there an association between the prevalence of atrial fibrillation and severity and control of blood pressure? the REGARDS study. Journal of the American Society of Hypertension Conference: 31st Annual Scientific Meeting of the American Society of Hypertension United States 2016;10 (4 Supplement):e14-e15.
(208) Kuwabara M, Niwa K, Nishihara S, Nishi Y, Takahashi O, Kario K, Yamamoto K, Yamashita T, Hisatome I. Hyperuricemia is an independent competing risk factor for atrial fibrillation. Int J Cardiol 2017 Mar 15;231:137-42.
(209) Yang Y, Han X, Chen Y, Gao L, Yin X, Li H, Qiu J, Wang Y, Zhou Y, Xia Y. Association between modifiable lifestyle and the prevalence of atrial fibrillation in a Chinese population: Based on the cardiovascular health score. Clin Cardiol 2017 Nov;40(11):1061-7.
(210) Deng H, Guo P, Zheng M, Huang J, Xue Y, Zhan X, Wang F, Liu Y, Fang X, Liao H, Wei W, Liang Y, et al. Epidemiological Characteristics of Atrial Fibrillation in Southern China: Results from the Guangzhou Heart Study. Sci Rep 2018 Dec 13;8(1):17829.
(211) Berge T, Lyngbakken MN, Ihle-Hansen H, Brynildsen J, Pervez MO, Aagaard EN, Vigen T, Kvisvik B, Christophersen IE, Steine K, Omland T, Smith P, et al. Prevalence of atrial fibrillation and cardiovascular risk factors in a 63-65 years old general population cohort: the Akershus Cardiac Examination (ACE) 1950 Study. BMJ Open 2018 Aug 1;8(7):e021704.
(212) Chen Y, Huang QF, Sheng CS, Lei L, Xu SK, Zhang W, Shao S, Wang D, Cheng YB, Wang Y, Guo QH, Zhang DY, et al. Cross-sectional Association Between Blood Pressure Status and Atrial Fibrillation in an Elderly Chinese Population. Am J Hypertens 2019 Jul 17;32(8):777-85.
(213) Tegene E, Tadesse I, Markos Y, Gobena T. Prevalence and risk factors for atrial fibrillation and its anticoagulant requirement in adults aged >/=40 in Jimma Town, Southwest Ethiopia: A community based cross-sectional study. Int J Cardiol Heart Vasc 2019 Mar;22:199-204.
(214) Guo Y, Gao J, Ye P, Xing A, Wu Y, Wu S, Luo Y. Comparison of atrial fibrillation in CKD and non-CKD populations: A cross-sectional analysis from the Kailuan study. Int J Cardiol 2019 Feb 15;277:125-9.
(215) Zubaid R, Ridha M, Akbar M. Characteristics and outcomes of patients with atrial fibrillation in kuwait. European Heart Journal, Supplement Conference: 12th Annual

Meeting of the Gulf Heart Association: Proceedings of a growing Middle Eastern Cardiac Society Kuwait 2019;17 (Supplement E):September.
(216) Zubaid R, Ridha M, Akbar M. Influence of gender on presentation and outcomes of atrial fibrillation in kuwait. European Heart Journal, Supplement Conference: 12th Annual Meeting of the Gulf Heart Association: Proceedings of a growing Middle Eastern Cardiac Society Kuwait 2019;17 (Supplement E):September.
(217) Ntaios G, Synetou M, Saliaris M, Xynos K, Pappa D, Savvari P, Spengos K, Vemmos K. Prevalence of atrial fibrillation in a greek rural population. Cerebrovascular Diseases Conference: 2019;May.
(218) Xing L, Lin M, Du Z, Jing L, Tian Y, Yan H, Ren G, Dong Y, Sun Q, Dai D, Shi L, Chen H, et al. Epidemiology of atrial fibrillation in northeast China: a cross-sectional study, 2017-2019. Heart 2020 Oct 13;106(8):590-5.
(219) Kowlgi GN, Gunda S, Padala SK, Koneru JN, Deshmukh AJ, Ellenbogen KA. Comparison of Frequency of Atrial Fibrillation in Blacks Versus Whites and the Utilization of Race in a Novel Risk Score. Am J Cardiol 2020 Aug 28;135:68-76.
(220) Joseph PG, Healey JS, Raina P, Connolly SJ, Ibrahim Q, Gupta R, Avezum A, Dans AL, Lopez-Jaramillo P, Yeates K, Teo K, Douma R, et al. Global variations in the prevalence, treatment, and impact of atrial fibrillation in a multi-national cohort of 153,152 middle-aged individuals. Cardiovasc Res 2020 Aug 10;doi: 10.1093/cvr/cvaa241.
(221) Guo Y, Wang H, Zhang H, Chen Y, Lip GYH. Population-based screening or targeted screening based on initial clinical risk assessment for atrial fibrillation: A report from the Huawei heart study. Journal of Clinical Medicine 2020;8(5):1493.
(222) Rivington J, Twohig P. Quantifying risk factors for atrial fibrillation: Retrospective review of a large electronic patient database. Journal of Atrial Fibrillation 13(3) (no pagination), 2020;October.
(223) Harding BN, Nor by FL, Heckbert SR, McKnight B, Psaty BM, Soliman EZ, Floyd J, Chen LY. Longitudinal measures of blood pressure and subclinical atrial arrhythmias: The multi-ethnic study of atherosclerosis and the atherosclerosis risk in communities study. Circulation Conference: American Heart Association's Epidemiology and Prevention/Lifestyle and Cardiometabolic Health 2021;(SUPPL 1):May.
(224) Zarifis J, Beevers G, Lip GYH. Acute admissions with atrial fibrillation in a British multiracial hospital population. British Journal of Clinical Practice 1997;51(2):91-4, 96.
(225) Lip GYH, Bawden L, Hodson R, Rutland E, Snatchfold J, Beevers DG. Atrial fibrillation amongst the Indo-Asian general practice population. The West Birmingham Atrial Fibrillation Project. International Journal of Cardiology 1998;65(2):187-92.
(226) Ridha M, Amin AAS, Saleh SA, Cherian B, Al-Kandari F, Redha F, Jamal K, Bitar Z. The prevalence of atrial fibrillation among acute medical admissions in Kuwait. Medical Principles and Practice 2005;14(3):136-9.
(227) Tveit A, Abdelnoor M, Enger S, Smith P. Atrial fibrillation and antithrombotic therapy in a 75-year-old population. Cardiology 2008;109(4):258-62.
(228) Ntep-Gweth M, Zimmermann M, Meiltz A, Kingue S, Ndobo P, Urban P, Bloch A. Atrial fibrillation in Africa: Clinical characteristics, prognosis, and adherence to guidelines in Cameroon. Europace 2010;12(4):482-7.
(229) Soliman EZ, Prineas RJ, Go AS, Xie D, Lash JP, Rahman M, Ojo A, Teal VL, Jensvold NG, Robinson NL, Dries DL, Bazzano L, et al. Chronic kidney disease and prevalent atrial fibrillation: The Chronic Renal Insufficiency Cohort (CRIC). American Heart Journal 2010;159(6):1102-7.
(230) Huang J-Y, Liu L, Yu Y-L, Chen C-L, Lo K, Zhang B, Tang S-T, Huang Y-Q, Feng Y-Q. A nonlinear relationship between low-density-lipoprotein cholesterol levels and atrial fibrillation among patients with hypertension in China. Annals of palliative medicine 2020;9(5):2953-61.

(231) Garcia-Acuna JM, Gonzalez-Juanatey JR, Ezquerra EA, Gonzalez M, I, Listerri JL. Permanent atrial fibrillation in heart disease in Spain. The CARDIOTENS study 1999. [Spanish]. Revista espanola de cardiologia 2002;55(9):943-52.
(232) Kannel WB, Abbott RD, Savage DD, McNamara PM. Epidemiologic features of chronic atrial fibrillation: the Framingham study. N Engl J Med 1982 Apr 29;306(17):1018-22.
(233) Onundarson PT, Thorgeirsson G, Jonmundsson E. Chronic atrial fibrillation - Epidemiologic features and 14 year follow-up: A case control study. European Heart Journal 1987;8(5):521-7.
(234) Furberg CD, Psaty BM, Manolio TA, Gardin JM, Smith VE, Rautaharju PM. Prevalence of atrial fibrillation in elderly subjects (the Cardiovascular Health Study). Am J Cardiol 1994 Aug 1;74(3):236-41.
(235) Benjamin EJ, Levy D, Vaziri SM, D'Agostino RB, Belanger AJ, Wolf PA. Independent risk factors for atrial fibrillation in a population-based cohort. The Framingham Heart Study. JAMA 1994 Mar 16;271(11):840-4.
(236) Psaty BM, Manolio TA, Kuller LH, Kronmal RA, Cushman M, Fried LP, White R, Furberg CD, Rautaharju PM. Incidence of and risk factors for atrial fibrillation in older adults. Circulation 1997 Oct 7;96(7):2455-61.
(237) Kannel WB, Wolf PA, Benjamin EJ, Levy D. Prevalence, incidence, prognosis, and predisposing conditions for atrial fibrillation: population-based estimates. Am J Cardiol 1998 Oct 16;82(8A):2N-9N.
(238) Schnabel RB, Sullivan LM, Levy D, Pencina MJ, Massaro JM, D'Agostino RB, Sr., Newton-Cheh C, Yamamoto JF, Magnani JW, Tadros TM, Kannel WB, Wang TJ, et al. Development of a risk score for atrial fibrillation (Framingham Heart Study): a community-based cohort study. Lancet 2009 Feb 28;373(9665):739-45.
(239) Schnabel RB, Aspelund T, Li G, Sullivan LM, Suchy-Dicey A, Harris TB, Pencina MJ, D'Agostino RB, Sr., Levy D, Kannel WB, Wang TJ, Kronmal RA, et al. Validation of an atrial fibrillation risk algorithm in whites and African Americans. Arch Intern Med 2010 Nov 22;170(21):1909-17.
(240) Chamberlain AM, Agarwal SK, Ambrose M, Folsom AR, Soliman EZ, Alonso A. Metabolic syndrome and incidence of atrial fibrillation among blacks and whites in the Atherosclerosis Risk in Communities (ARIC) Study. Am Heart J 2010 May;159(5):850-6.
(241) Tanner RM, Baber U, Carson AP, Voeks J, Brown TM, Soliman EZ, Howard VJ, Muntner P. Association of the metabolic syndrome with atrial fibrillation among United States adults (from the REasons for Geographic and Racial Differences in Stroke [REGARDS] Study). Am J Cardiol 2011 Jul 15;108(2):227-32.
(242) Huxley RR, Lopez FL, Folsom AR, Agarwal SK, Loehr LR, Soliman EZ, Maclehose R, Konety S, Alonso A. Absolute and attributable risks of atrial fibrillation in relation to optimal and borderline risk factors: the Atherosclerosis Risk in Communities (ARIC) study. Circulation 2011 Apr 12;123(14):1501-8.
(243) Everett BM, Cook NR, Conen D, Chasman DI, Ridker PM, Albert CM. Novel genetic markers improve measures of atrial fibrillation risk prediction. European Heart Journal 2013;34(29):2243-51.
(244) Cheng YF, Leu H-B, Su CC, Huang CC, Chiang CH, Huang PH, Chung CM, Lin SJ, Chen JW, Chan WL. Association between panic disorder and risk of atrial fibrillation: A nationwide study. Psychosomatic Medicine 2013;75(1):30-5.
(245) Grundvold I, Skretteberg PT, Liestol K, Erikssen G, Engeseth K, Gjesdal K, Kjeldsen SE, Arnesen H, Erikssen J, Bodegard J. Low heart rates predict incident atrial fibrillation in healthy middle-aged men. Circulation: Arrhythmia and Electrophysiology 2013;6(4):726-31.
(246) O'Neal WT, Soliman EZ, Qureshi W, Alonso A, Heckbert SR, Herrington D. Sustained pre-hypertensive blood pressure and incident atrial fibrillation: the Multi-Ethnic Study of Atherosclerosis. J Am Soc Hypertens 2015 Mar;9(3):191-6.

(247) Rodriguez F, Stefanick ML, Greenland P, Soliman EZ, Manson JE, Parikh N, Martin LW, Larson JC, Hlatky M, Nassir R, Cene CW, Rodriguez BL, et al. Racial and ethnic differences in atrial fibrillation risk factors and predictors in women: Findings from the Women's Health Initiative. Am Heart J 2016 Jun;176:70-7.
(248) Son MK, Lim NK, Cho MC, Park HY. Incidence and Risk Factors for Atrial Fibrillation in Korea: the National Health Insurance Service Database (2002-2010). Korean Circ J 2016 Jul;46(4):515-21.
(249) Kokubo Y, Watanabe M, Higashiyama A, Nakao YM, Kusano K, Miyamoto Y. Development of a Basic Risk Score for Incident Atrial Fibrillation in a Japanese General Population- The Suita Study. Circ J 2017 Oct 25;81(11):1580-8.
(250) O'Neal WT, Judd SE, Limdi NA, McIntyre WF, Kleindorfer DO, Cushman M, Howard VJ, Howard G, Soliman EZ. Differential Impact of Risk Factors in Blacks and Whites in the Development of Atrial Fibrillation: the Reasons for Geographic And Racial Differences in Stroke (REGARDS) Study. J Racial Ethn Health Disparities 2017 Aug;4(4):718-24.
(251) Lee SS, Ae KK, Kim D, Lim YM, Yang PS, Yi JE, Kim M, Kwon K, Bum PW, Joung B, Park J. Clinical implication of an impaired fasting glucose and prehypertension related to new onset atrial fibrillation in a healthy Asian population without underlying disease: a nationwide cohort study in Korea. Eur Heart J 2017 Sep 7;38(34):2599-607.
(252) Lee H, Choi EK, Lee SH, Han KD, Rhee TM, Park CS, Lee SR, Choe WS, Lim WH, Kang SH, Cha MJ, Oh S. Atrial fibrillation risk in metabolically healthy obesity: A nationwide population-based study. Int J Cardiol 2017 Aug 1;240:221-7.
(253) Garg PK, O'Neal WT, Ogunsua A, Thacker EL, Howard G, Soliman EZ, Cushman M. Usefulness of the American Heart Association's Life Simple 7 to Predict the Risk of Atrial Fibrillation (from the REasons for Geographic And Racial Differences in Stroke [REGARDS] Study). Am J Cardiol 2018 Jan 15;121(2):199-204.
(254) Li YG, Pastori D, Farcomeni A, Yang PS, Jang E, Joung B, Wang YT, Guo YT, Lip GYH. A Simple Clinical Risk Score (C2HEST) for Predicting Incident Atrial Fibrillation in Asian Subjects: Derivation in 471,446 Chinese Subjects, With Internal Validation and External Application in 451,199 Korean Subjects. Chest 2019 Mar;155(3):510-8.
(255) Kwon CH, Kim H, Kim SH, Kim BS, Kim HJ, Kim DK, Han SW, Ryu KH, Dong SJ. The Impact of Metabolic Syndrome on the Incidence of Atrial Fibrillation: A Nationwide Longitudinal Cohort Study in South Korea. J Clin Med 2019 Jul 24;8(8).
(256) Bundy JD, Heckbert SR, Chen LY, Lloyd-Jones DM, Greenland P. Evaluation of Risk Prediction Models of Atrial Fibrillation (from the Multi-Ethnic Study of Atherosclerosis [MESA]). Am J Cardiol 2020 Jan 1;125(1):55-62.
(257) Kim YG, Han KD, Choi JI, Choi YY, Choi HY, Boo KY, Kim DY, Lee KN, Shim J, Kim JS, Park YG, Kim YH. Non-genetic risk factors for atrial fibrillation are equally important in both young and old age: A nationwide population-based study. Eur J Prev Cardiol 2020 Apr 10;2047487320915664.
(258) Park YJ, Yang P-S, Yu HT, Kim T-H, Jang E, Uhm J-S, Pak H-N, Lee M-H, Lip GYH, Joung B. What is the ideal blood pressure threshold for the prevention of atrial fibrillation in elderly general population? Journal of Clinical Medicine 2020;9(9):2988.
(259) Ahn H-J, Han K-D, Choi E-K, Jung J-H, Kwon S, Lee S-R, Oh S, Lip GYH. Cumulative burden of metabolic syndrome and its components on the risk of atrial fibrillation: a nationwide population-based study. Cardiovascular Diabetology 2020;(1):20.
(260) Khurshid S, Kartoun U, Ashburner JM, Trinquart L, Philippakis A, Khera AV, Ellinor PT, Ng K, Lubitz SA. Performance of Atrial Fibrillation Risk Prediction Models in Over 4 Million Individuals. Circ Arrhythm Electrophysiol 2021 Jan;14(1):e008997.
(261) Lee SR, Park CS, Choi EK, Ahn HJ, Han KD, Oh S, Lip GYH. Hypertension Burden and the Risk of New-Onset Atrial Fibrillation: A Nationwide Population-Based Study. Hypertension 2021 Mar 3;77(3):919-28.
(262) Kaneko H, Yano Y, Itoh H, Morita K, Kiriyama H, Kamon T, Fujiu K, Michihata N, Jo T, Takeda N, Morita H, Node K, et al. Association of Blood Pressure Classification Using the

2017 American College of Cardiology/American Heart Association Blood Pressure Guideline With Risk of Heart Failure and Atrial Fibrillation. Circulation 2021 Jun 8;143(23):2244-53.
(263) Itoh H, Kaneko H, Fujiu K, Kiriyama H, Morita K, Kamon T, Michihata N, Jo T, Takeda N, Morita H, Yasunaga H, Komuro I. Risk Factors and Lifestyles in the Development of Atrial Fibrillation Among Individuals Aged 20-39 Years. Am J Cardiol 2021 Sep 15;155:40-4.
(264) Lee SR, Han KD, Choi EK, Ahn HJ, Oh S, Lip GYH. Risk of Atrial Fibrillation in Young Adults With Isolated Diastolic, Isolated Systolic, and Systolic-Diastolic Hypertension. Hypertension 2021 Nov;78(5):1248-58.
(265) Kim IS, Choi YJ, Choi EY, Min PK, Yoon YW, Lee BK, Hong BK, Rim SJ, Kwon HM, Kim JY. Comparison of risk profiles for new-onset atrial fibrillation between patients aged <60 and â‰¥60 years. PLoS One 2021;16(11):e0258770.
(266) Liao JN, Lim SS, Chen TJ, Tuan TC, Chen SA, Chao TF. Modified Taiwan Atrial Fibrillation Score for the Prediction of Incident Atrial Fibrillation. Front Cardiovasc Med 2021;8:805399.
(267) Huang K, Trinder M, Roston TM, Laksman ZW, Brunham LR. The Interplay Between Titin, Polygenic Risk, and Modifiable Cardiovascular Risk Factors in Atrial Fibrillation. Canadian Journal of Cardiology 37(6) (pp 848-856), 2021;June.
(268) Lee S-R, Han K-D, Choi E-K, Ahn H-J, Oh S, Lip GYH. Risk of Atrial Fibrillation in Young Adults with Isolated Diastolic, Isolated Systolic, and Systolic-Diastolic Hypertension. Hypertension (pp 1248-1258), 2021;2021.
(269) Kim YG, Han K-D, Kim DY, Choi YY, Choi HY, Roh S-Y, Shim J, Kim JS, Choi J-I, Kim Y-H. Different Influence of Blood Pressure on New-Onset Atrial Fibrillation in Pre-and Postmenopausal Women: A Nationwide Population-Based Study. Hypertension 77(5) (pp 1500-1509), 2021;01.
(270) Park YJ, Yang PS, Yu HT, Kim TH, Jang E, Uhm JS, Pak HN, Lee MH, Lip GYH, Joung B. Association of proteinuria and hypertension with incident atrial fibrillation in an elderly population: nationwide data from a community-based elderly cohort. J Hypertens 2022 Jan 1;40(1):128-35.
(271) Morseth B, Geelhoed B, Linneberg A, Johansson L, Kuulasmaa K, Salomaa V, Iacoviello L, Costanzo S, Soderberg S, Niiranen TJ, Vishram-Nielsen JKK, Njolstad I, et al. Age-specific atrial fibrillation incidence, attributable risk factors and risk of stroke and mortality: Results from the MORGAM Consortium. Open Heart 8(2) (no pagination), 2021;e001624.
(272) Tsai W-C, Chen C-Y, Kuo H-F, Wu M-T, Tang W-H, Chu C-S, Lin T-H, Su H-M, Hsu P-C, Jhuo S-J, Lin M-Y, Lee K-T, et al. Areca nut chewing and risk of atrial fibrillation in Taiwanese men: A nationwide Ecological Study. International Journal of Medical Sciences 2013;10(7):804-11.
(273) Verdecchia P, Mazzotta G, Angeli F, Reboldi G. Above which blood pressure level does the risk of atrial fibrillation increase? Hypertension 2012 Feb;59(2):184-5.
(274) Luscher TF. Risk factors and consequences of atrial fibrillation: Genetics, blood pressure, working hours, and cognitive decline. European Heart Journal 2017;38(34):2573-5.
(275) McCabe PJ, Darbar D. Is achieving the american heart association's life simple 7 goals sufficient to reduce the burden of atrial fibrillation? No simple answers. Journal of the American Heart Association 2018;7(8):e009127.
(276) Shah DC. Longitudinal, long-term evaluation of new-onset atrial fibrillation: the interplay of blood pressure burden over time and gender. Eur Heart J 2020 Jun 3;41(16):1563-4.
(277) Manolis A, Doumas M. Atrial fibrillation, arterial hypertension, and primary aldosteronism: a dangerous and unexpected trio. J Hypertens 2020 Feb;38(2):208-10.
(278) Sala LL, Pontiroli AE. Role of obesity and hypertension in the incidence of atrial fibrillation, ischaemic heart disease and heart failure in patients with diabetes. Cardiovascular Diabetology 2020;(1):162.

(279) Pierdomenico SD, Ianni U, De RM, Coccina F. Different measures of blood pressure and incident atrial fibrillation. J Hypertens 2021 Jan;39(1):38-41.
(280) Liatakis I, Manta E, Tsioufis C. Hypertension and Atrial Fibrillation: Epidemiological Data, Pathogenesis, and Therapeutic Implications. Am J Hypertens 2019 Jul 17;32(8):725-6.
(281) Aronow WS. Hypertension associated with atrial fibrillation. Annals of Translational Medicine 2017;5(23):457.
(282) Watanabe H, Tanabe N, Makiyama Y, Chopra SS, Okura Y, Suzuki H, Matsui K, Watanabe T, Kurashina Y, Aizawa Y. ST-segment abnormalities and premature complexes are predictors of new-onset atrial fibrillation: the Niigata preventive medicine study. Am Heart J 2006 Oct;152(4):731-5.
(283) Watanabe H, Tanabe N, Watanabe T, Darbar D, Roden DM, Sasaki S, Aizawa Y. Metabolic syndrome and risk of development of atrial fibrillation: the Niigata preventive medicine study. Circulation 2008 Mar 11;117(10):1255-60.
(284) Weng LC, Lunetta KL, Muller-Nurasyid M, Smith AV, Theriault S, Weeke PE, Barnard J, Bis JC, Lyytikainen LP, Kleber ME, Martinsson A, Lin HJ, et al. Genetic Interactions with Age, Sex, Body Mass Index, and Hypertension in Relation to Atrial Fibrillation: The AFGen Consortium. Sci Rep 2017 Sep 12;7(1):11303.
(285) Li Y-G, Bai J, Zhou G, Li J, Wei Y, Sun L, Zu L, Liu S. Refining age stratum of the C2HEST score for predicting incident atrial fibrillation in a hospital-based Chinese population. European Journal of Internal Medicine 90 (pp 37-42), 2021;August.
(286) Korantzopoulos P, Kolettis T, Goudevenos J. Atrial fibrillation in hypertension: an established association with several unresolved issues. Cardiology 2003;100(2):105-6.
(287) Conen D, Tedrow UB, Koplan BA, Glynn RJ, Buring JE, Albert CM. Response to letter regarding article, "influence of systolic and diastolic blood pressure on the risk of incident atrial fibrillation in women". Circulation 2010;121(5):e30.
(288) Barrios V, Escobar C, Echarri R. Letter by barrios et al regarding article, "influence of systolic and diastolic blood pressure on the risk of incident atrial fibrillation in women". Circulation 2010;121(5):e29.
(289) Lloyd-Jones DM, Wang TJ, Leip EP, Larson MG, Levy D, Vasan RS, D'Agostino RB, Massaro JM, Beiser A, Wolf PA, Benjamin EJ. Lifetime risk for development of atrial fibrillation: the Framingham Heart Study. Circulation 2004 Aug 31;110(9):1042-6.
(290) Staerk L, Wang B, Preis SR, Larson MG, Lubitz SA, Ellinor PT, McManus DD, Ko D, Weng LC, Lunetta KL, Frost L, Benjamin EJ, et al. Lifetime risk of atrial fibrillation according to optimal, borderline, or elevated levels of risk factors: cohort study based on longitudinal data from the Framingham Heart Study. BMJ 2018 Apr 26;361:k1453.
(291) Coccina F, Pierdomenico AM, De RM, Cuccurullo C, Pierdomenico SD. Association of clinic and ambulatory blood pressure with new-onset atrial fibrillation: A meta-analysis of observational studies. J Clin Hypertens (Greenwich) 2021 Jun;23(6):1104-11.
(292) Zheng Y, Xie Z, Li J, Chen C, Cai W, Dong Y, Xue R, Liu C. Meta-analysis of metabolic syndrome and its individual components with risk of atrial fibrillation in different populations. BMC Cardiovasc Disord 2021 Feb 15;21(1):90.
(293) He L, Culminskaya I, Loika Y, Arbeev KG, Bagley O, Duan M, Yashin AI, Kulminski AM. Causal effects of cardiovascular risk factors on onset of major age-related diseases: A time-to-event Mendelian randomization study. Exp Gerontol 2018 Jul 1;107:74-86.
(294) Hyman MC, Levin MG, Gill D, Walker VM, Georgakis MK, Davies NM, Marchlinski FE, Damrauer SM. Genetically Predicted Blood Pressure and Risk of Atrial Fibrillation. Hypertension 2021 Feb;77(2):376-82.
(295) Georgiopoulos G, Ntritsos G, Stamatelopoulos K, Tsioufis C, Aimo A, Masi S, Evangelou E. The relationship between blood pressure and risk of atrial fibrillation: a Mendelian randomization study. Eur J Prev Cardiol 2021 Feb 9.
(296) Nazarzadeh M, Pinho-Gomes AC, Bidel Z, Canoy D, Dehghan A, Smith BK, Bennett DA, Smith GD, Rahimi K. Genetic susceptibility, elevated blood pressure, and risk of atrial fibrillation: a Mendelian randomization study. Genome Med 2021 Mar 4;13(1):38.

(297) Nazarzadeh M, Pinho-Gomes A-C, Bidel Z, Canoy D, Dehghan A, Byrne KS, Bennett DA, Smith GD, Rahimi K. Genetic susceptibility, elevated blood pressure and risk of atrial fibrillation. Journal of Hypertension Conference: European Meeting on Hypertension and Cardiovascular Protection and International Society of Hypertension, ESH-ISH 2021;(SUPPL 1):April.
(298) Wang Q, Richardson TG, Sanderson E, Tudball MJ, la-Korpela M, Davey SG, Holmes MV. A phenome-wide bidirectional Mendelian randomization analysis of atrial fibrillation. Int J Epidemiol 2022 Mar 15.
(299) Li HQ, Feng YW, Yang YX, Leng XY, Zhang PC, Chen SD, Kuo K, Huang SY, Zhang XQ, Dong Y, Han X, Cheng X, et al. Causal Relations between Exposome and Stroke: A Mendelian Randomization Study. J Stroke 2022 May;24(2):236-44.
(300) Schulman KL, Johnston SS, Lin J, Huse DM. Prevalence of atrial fibrillation (AF) in the US. Pharmacoepidemiology and Drug Safety Conference: 26th International Conference on Pharmacoepidemiology and Therapeutic Risk Management Brighton United Kingdom Conference Publication 2019;(SUPPL. 1):August.
(301) Fatema K, Barnes ME, Bailey KR, Abhayaratna WP, Cha S, Seward JB, Tsang TSM. Minimum vs. maximum left atrial volume for prediction of first atrial fibrillation or flutter in an elderly cohort: A prospective study. European Journal of Echocardiography 2009;10(2):282-6.
(302) Macfarlane PW, Murray H, Sattar N, Stott DJ, Ford I, Buckley B, Jukema JW, Westendorp RG, Shepherd J. The incidence and risk factors for new onset atrial fibrillation in the PROSPER study. Europace 2011 May;13(5):634-9.
(303) Hill NR, Ayoubkhani D, McEwan P, Sugrue DM, Farooqui U, Lister S, Lumley M, Bakhai A, Cohen AT, O'Neill M, Clifton D, Gordon J. Predicting atrial fibrillation in primary care using machine learning. PLoS One 2019;14(11):e0224582.
(304) Williams BA, Chamberlain AM, Blankenship JC, Hylek EM, Voyce S. Trends in Atrial Fibrillation Incidence Rates Within an Integrated Health Care Delivery System, 2006 to 2018. JAMA Netw Open 2020 Aug 3;3(8):e2014874.
(305) Chen SK, Barbhaiya M, Solomon DH, Guan H, Yoshida K, Feldman CH, Everett BM, Costenbader KH. Atrial Fibrillation/Flutter Hospitalizations among U.S. Medicaid Recipients with and without Systemic Lupus Erythematosus. The Journal of rheumatology 2020;47(9):1359-65.
(306) Bunch TJ, Packer DL, Jahangir A, Locke GR, Talley NJ, Gersh BJ, Roy RR, Hodge DO, Asirvatham SJ. Long-Term Risk of Atrial Fibrillation With Symptomatic Gastroesophageal Reflux Disease and Esophagitis. American Journal of Cardiology 2008;102(9):1207-11.
(307) Iguchi Y, Kimura K, Shibazaki K, Aoki J, Kobayashi K, Sakai K, Sakamoto Y. Annual incidence of atrial fibrillation and related factors in adults. American Journal of Cardiology 2010;106(8):1129-33.
(308) Dai H, Zhang Q, Much AA, Maor E, Segev A, Beinart R, Adawi S, Lu Y, Bragazzi NL, Wu J. Global, regional, and national prevalence, incidence, mortality, and risk factors for atrial fibrillation, 1990-2017: results from the Global Burden of Disease Study 2017. Eur Heart J Qual Care Clin Outcomes 2020 Jul 31;doi: 10.1093/ehjqcco/qcaa061.
(309) Chen MM, Zhang X, Liu YM, Chen Z, Li H, Lei F, Qin JJ, Ji Y, Zhang P, Cai J, She ZG, Zhang XJ, et al. Heavy Disease Burden of High Systolic Blood Pressure During 1990-2019: Highlighting Regional, Sex, and Age Specific Strategies in Blood Pressure Control. Front Cardiovasc Med 2021;8:754778.
(310) Al Chekakie MO, Welles CC, Metoyer R, Ibrahim A, Shapira AR, Cytron J, Santucci P, Wilber DJ, Akar JG. Pericardial fat is independently associated with human atrial fibrillation. Journal of the American College of Cardiology 2010;56(10):784-8.
(311) Brand FN, Abbott RD, Kannel WB, Wolf PA. Characteristics and prognosis of lone atrial fibrillation. 30-year follow-up in the Framingham Study. JAMA 1985 Dec 27;254(24):3449-53.

(312) Verdecchia P, Reboldi G, Gattobigio R, Bentivoglio M, Borgioni C, Angeli F, Carluccio E, Sardone MG, Porcellati C. Atrial fibrillation in hypertension: predictors and outcome. Hypertension 2003 Feb;41(2):218-23.
(313) Wachtell K, Lehto M, Gerdts E, Olsen MH, Hornestam B, Dahlof B, Ibsen H, Julius S, Kjeldsen SE, Lindholm LH, Nieminen MS, Devereux RB. Angiotensin II receptor blockade reduces new-onset atrial fibrillation and subsequent stroke compared to atenolol: The Losartan Intervention for End point reduction in hypertension (LIFE) study. Journal of the American College of Cardiology 2005;45(5):712-9.
(314) Asselbergs FW, Moore JH, van den Berg MP, Rimm EB, De Boer RA, Dullaart RP, Navis G, van Gilst WH. A role for CETP TaqIB polymorphism in determining susceptibility to atrial fibrillation: A nested case control study. BMC Medical Genetics 2006;7:39.
(315) Okin PM, Wachtell K, Kjeldsen SE, Julius S, Lindholm LH, Dahlof B, Hille DA, Nieminen MS, Edelman JM, Devereux RB. Incidence of atrial fibrillation in relation to changing heart rate over time in hypertensive patients: the LIFE study. Circulation Arrhythmia and electrophysiology 2008;1(5):337-43.
(316) Ravn LS, Benn M, Nordestgaard BG, Sethi AA, gerholm-Larsen B, Jensen GB, Tybjaerg-Hansen A. Angiotensinogen and ACE gene polymorphisms and risk of atrial fibrillation in the general population. Pharmacogenet Genomics 2008 Jun;18(6):525-33.
(317) Heckbert SR, Wiggins KL, Glazer NL, Dublin S, Psaty BM, Smith NL, Longstreth WT, Jr., Lumley T. Antihypertensive treatment with ACE inhibitors or beta-blockers and risk of incident atrial fibrillation in a general hypertensive population. Am J Hypertens 2009 May;22(5):538-44.
(318) Conen D, Tedrow UB, Cook NR, Buring JE, Albert CM. Birth weight is a significant risk factor for incident atrial fibrillation. Circulation 2010;122(8):764-70.
(319) Fedorowski A, Hedblad B, EngstrÃ¶m G, Gustav SJ, Melander O. Orthostatic hypotension and long-term incidence of atrial fibrillation: the MalmÃ¶ Preventive Project. J Intern Med 2010 Oct;268(4):383-9.
(320) Mattioli AV, Farinetti A, Miloro C, Pedrazzi P, Mattioli G. Influence of coffee and caffeine consumption on atrial fibrillation in hypertensive patients. Nutrition, Metabolism and Cardiovascular Diseases 2011;21(6):412-7.
(321) Everett BM, Conen D, Buring JE, Moorthy MV, Lee I-M, Albert CM. Physical activity and the risk of incident atrial fibrillation in women. Circulation: Cardiovascular Quality and Outcomes 2011;4(3):321-7.
(322) Non-aspirin NSAIDs increase risk of atrial fibrillation. Australian Journal of Pharmacy 2011;92(1097):85.
(323) Magnani JW, Johnson VM, Sullivan LM, Gorodeski EZ, Schnabel RB, Lubitz SA, Levy D, Ellinor PT, Benjamin EJ. P wave duration and risk of longitudinal atrial fibrillation in persons <60 years old (from the Framingham heart study). American Journal of Cardiology 2011;107(6):917-21.
(324) Rienstra M, Cheng S, Larson MG, McCabe EL, Booth SL, Jacques PF, Lubitz SA, Yin X, Levy D, Magnani JW, Ellinor PT, Benjamin EJ, et al. Vitamin D status is not related to development of atrial fibrillation in the community. American Heart Journal 2011;162(3):538-41.
(325) Tamariz L, Agarwal S, Soliman EZ, Chamberlain AM, Prineas R, Folsom AR, Ambrose M, Alonso A. Association of serum uric acid with incident atrial fibrillation (from the Atherosclerosis Risk in Communities [ARIC] study). American Journal of Cardiology 2011;108(9):1272-6.
(326) Shibata Y, Watanabe T, Osaka D, Abe S, Inoue S, Tokairin Y, Igarashi A, Yamauchi K, Kimura T, Kishi H, Aida Y, Nunomiya K, et al. Impairment of pulmonary function is an independent risk factor for atrial fibrillation: The takahata study. International Journal of Medical Sciences 2011;8(7):514-22.
(327) Sinner MF, Lubitz SA, Pfeufer A, Makino S, Beckmann B-M, Lunetta KL, Steinbeck G, Perz S, Rahman R, Sonni A, Greenberg SM, Furie KL, et al. Lack of replication in

polymorphisms reported to be associated with atrial fibrillation. Heart Rhythm 2011;8(3):403-9.
(328) Smith MB, May HT, Blair TL, Anderson JL, Muhlestein JB, Horne BD, Lappe DL, Day JD, Crandall BG, Weiss P, Osborn JS, Bunch TJ. Vitamin d excess is significantly associated with risk of atrial fibrillation. Circulation Conference: American Heart Association's Scientific Sessions 2011;22.
(329) Sandhu RK, Kurth T, Conen D, Cook NR, Ridker PM, Albert CM. Impaired renal function is associated with an increased risk for incident atrial fibrillation. Circulation Conference: American Heart Association's Scientific Sessions 2011;22.
(330) Misialek JR, Rose KM, Everson-Rose SA, Soliman EZ, Clark CJ, Lopez FL, Alonso A. Socioeconomic status and the incidence of atrial fibrillation in whites and African Americans: The atherosclerosis risk in communities (ARIC) study. Circulation Conference: American Heart Association's Scientific Sessions 2011;22.
(331) Schoen T, Pradhan AD, Albert CM, Conen D. Diabetes and risk of atrial fibrillation in women. European Heart Journal Conference: European Society of Cardiology, ESC Congress 2011;August.
(332) Watanabe H, Tanabe N, Yagihara N, Watanabe T, Aizawa Y, Kodama M. Association between lipid profile and risk of atrial fibrillation. Circ J 2011;75(12):2767-74.
(333) Khawaja O, Bartz TM, Ix JH, Heckbert SR, Kizer JR, Zieman SJ, Mukamal KJ, Tracy RP, Siscovick DS, Djousse L. Plasma free fatty acids and risk of atrial fibrillation (from the Cardiovascular Health Study). American Journal of Cardiology 2012;110(2):212-6.
(334) Nyrnes A, Njolstad I, Mathiesen EB, Wilsgaard T, Hansen J-B, Skjelbakken T, Jorgensen L, Lochen M-L. Inflammatory biomarkers as risk factors for future atrial fibrillation. an eleven-year follow-up of 6315 men and women: The Tromso study. Gender Medicine 2012;9(6):536-47.
(335) Rosenberg MA, Gottdiener JS, Heckbert SR, Mukamal KJ. Echocardiographic diastolic parameters and risk of atrial fibrillation: The cardiovascular health study. European Heart Journal 2012;33(7):904-12.
(336) Schoen T, Pradhan AD, Albert CM, Conen D. Type 2 diabetes mellitus and risk of incident atrial fibrillation in women. Journal of the American College of Cardiology 2012;60(15):1421-8.
(337) Perez MV, Wang PJ, Larson JC, Virnig BA, Cochrane B, Curb JD, Klein L, Manson JE, Martin LW, Robinson J, Wassertheil-Smoller S, Stefanick ML. Effects of postmenopausal hormone therapy on incident atrial fibrillation: The Women's Health Initiative randomized controlled trials. Circulation: Arrhythmia and Electrophysiology 2012;5(6):1108-16.
(338) Fontes JD, Lyass A, Massaro JM, Rienstra M, Dallmeier D, Schnabel RB, Wang TJ, Vasan RS, Lubitz SA, Magnani JW, Levy D, Ellinor PT, et al. Insulin resistance and atrial fibrillation (from the Framingham Heart Study). American Journal of Cardiology 2012;109(1):87-90.
(339) Nielsen JB, Pietersen A, Graff C, Lind B, Struijk J, Olesen MS, Haunsoe S, Koeber L, Svendsen JH, Holst AG. J-shaped association between p-wave duration and risk of atrial fibrillation: Results from the Copenhagen ECG study. Circulation Conference: American Heart Association 2012;20.
(340) Sinner MF, Krijthe B, Aspelund T, Stepas K, Pencina M, Moser C, Sootodehnia N, Fontes J, Janssens C, Kronmal R, Magnani J, Witteman J, et al. B-type-natriuretic peptide and c-reactive protein in the prediction of atrial fibrillation risk: The charge-AF consortium. Circulation Conference: American Heart Association 2012;20.
(341) Lopez FL, Agarwal SK, Soliman EZ, Loehr LR, Lutsey PL, Chen LY, Huxley RR, Alonso A. Serum phosphorus levels and the incidence of atrial fibrillation: The atherosclerosis risk in communities (ARIC) study. Circulation Conference: Epidemiology and Prevention/Physical Activity, Nutrition and Metabolism 2012;13.
(342) Hung C-Y, Lin C-H, Ting C-T, Wu T-J. CHADS2 score predicts the effectiveness of statin on primary prevention of atrial fibrillation: A nationwide cohort study. Heart Rhythm

Conference: 33rd Annual Scientific Sessions of the Heart Rhythm Society, Heart Rhythm 2012;May.
(343) Lau CP, Gbadebo DT, Connolly SJ, Van G, I, Capucci A, Gold MR, Israel CW, Morillo CA, Carlson MD, Siu CW, Tse HF, Hohnloser SH, et al. Influence of race on the development of atrial fibrillation: Results from the assert study. Heart Rhythm Conference: 33rd Annual Scientific Sessions of the Heart Rhythm Society, Heart Rhythm 2012;May.
(344) Agarwal SK, Alonso A, Whelton SP, Soliman EZ, Rose KM, Chamberlain AM, Simpson RJ, Jr., Coresh J, Heiss G. Orthostatic change in blood pressure and incidence of atrial fibrillation: results from a bi-ethnic population based study. PLoS One 2013;8(11):e79030.
(345) Tung Y-C, Wu L-S, Chen W-J, Kuo C-T, Wang C-L, Chang C-J, Tsai H-Y, Yeh Y-H, Hsu L-A. C-reactive protein gene polymorphisms and the risk of atrial fibrillation in a Chinese population in Taiwan. Acta Cardiologica Sinica 2013;29(3):208-16.
(346) Fretts AM, Mozaffarian D, Siscovick DS, Heckbert SR, McKnight B, King IB, Rimm EB, Psaty BM, Sacks FM, Song X, Spiegelman D, Lemaitre RN. Associations of plasma phospholipid and dietary alpha linolenic acid with incident atrial fibrillation in older adults: The cardiovascular health study. Journal of the American Heart Association 2013;2(1):e003814.
(347) Hung C-Y, Wu T-J, Wang K-Y, Huang J-L, Loh E-W, Chen Y-M, Lin C-S, Lin C-H, Chen D-Y, Tang Y-J. Falls and atrial fibrillation in elderly patients. Acta Cardiologica Sinica 2013;29(5):436-43.
(348) Facila L, Pallares V, Morillas P, Cordero A, Llisterri Luis JL, Sanchis C, Gorriz JL, Castillo J, Gil V, Redon J. Gender differences related to the presence of atrial fibrillation in older hypertensive patients. World Journal of Cardiology 2013;5(5):124-31.
(349) Misialek JR, Lopez FL, Lutsey PL, Huxley RR, Peacock JM, Chen LY, Soliman EZ, Agarwal SK, Alonso A. Serum and dietary magnesium and incidence of atrial fibrillation in whites and in african americans - Atherosclerosis risk in communities (ARIC) study. Circulation Journal 2013;77(2):323-9.
(350) Karppi J, Kurl S, Makikallio TH, Ronkainen K, Laukkanen JA. Low levels of plasma carotenoids are associated with an increased risk of atrial fibrillation. European Journal of Epidemiology 28 (1) (pp 45-53), 2013;28(1):45-53.
(351) Chao T-F, Liu C-J, Chen S-J, Wang K-L, Lin Y-J, Chang S-L, Lo L-W, Hu Y-F, Tuan T-C, Wu T-J, Chen T-J, Tsao H-M, et al. The association between the use of non-steroidal anti-inflammatory drugs and atrial fibrillation: A nationwide case-control study. International Journal of Cardiology 2013;168(1):312-6.
(352) Willeit K, Egger G, Oberhollenzer M, Willeit J, Kiechl S. The association of asymptomatic atherosclerosis and incident atrial fibrillation. Cerebrovascular Diseases Conference: 22nd European Stroke Conference London United Kingdom Conference Publication 2013;35 (SUPPL.3):485.
(353) Friedman DJ, Wang N, Meigs JB, Hoffmann U, Massaro JM, Fox CS, Magnani JW. Pericardial fat is associated with atrial electrical function: The framingham heart study. Circulation Conference: American Heart Association 2013;26.
(354) Verheyen N, Grubler M, Pilz S, Kienreich K, Gaksch M, Schmid J, Bersuch LA, Pieske B, Kraigher-Krainer E, Fahrleitner-Pammer A, Boehm BO, Marz W, et al. Serum parathyroid hormone is independently associated with atrial fibrillation: Insights from the LURIC study. Circulation Conference: American Heart Association 2013;26.
(355) Rix TA, Joensen AM, Lundbye-Christensen S, Riahi S, Schmidt EB, Overvad K. Moderate consumption of marine n-3 fatty acids is associated with a lower risk of atrial fibrillation-a cohort study. Europace Conference: European Heart Rhythm Association, EHRA EUROPACE 2013;June.
(356) Xia Y, Tian X, Gao I, Zhang S, Yin X, Chang D, Zhang R, Yang Y. Common variants in TBX5 associated with atrial fibrillation in Chinese Han population. Heart Rhythm Conference: 34th Annual Scientific Sessions of the Heart Rhythm Society, Heart Rhythm 2013;May.

(357) Amir AM, Lehmann N, Marie HB, Kalsch H, Bauer M, Kara K, Moebus S, Jockel K-H, Erbel R, Mohlenkamp S. Association of epicardial adipose tissue with prevalent and incident at rial fibrillation in the general population: The heinz nixdorf recall study. Journal of the American College of Cardiology Conference: 62nd Annual Scientific Session of the American College of Cardiology and i2 Summit: Innovation in Intervention, ACC 13 San Francisco, CA United States Conference Publication 2013;61.
(358) Macheret F, Bartz T, Djousse L, Ix J, Mukamal K, Zieman S, Siscovick D, Tracy R, Heckbert S, Psaty B, Kizer J. Higher circulating total and high-molecular-weight adiponectin are associated with increased risk of at rial fibrillation in older adults. Journal of the American College of Cardiology Conference: 62nd Annual Scientific Session of the American College of Cardiology and i2 Summit: Innovation in Intervention, ACC 13 San Francisco, CA United States Conference Publication 2013;61:12.
(359) Mahabadi AA, Lehmann N, Kalsch H, Bauer M, Dykun I, Moebus S, Jockel K-H, Erbel R, Mohlenkamp S. Epicardial adipose tissue and left atrial size as CT-derived predictors of atrial fibrillation: The heinz nixdorf recall study. European Heart Journal Conference: European Society of Cardiology, ESC Congress 2013;August.
(360) Kokubo Y, Watanabe M, Shimizu W, Kamakura S, Kada A, Kawanishi K, Miyamoto Y. Impact of smoking and excessive drinking on the risk of incident atrial fibrillation in the Suita study: An urban cohort study. European Heart Journal Conference: European Society of Cardiology, ESC Congress 2013;August.
(361) Bang CN, Okin P, Gottlieb AB, Kober L, Wachtell K, Devereux RB. Psoriasis is associated with subsequent atrial fibrillation in hypertensive patients. the life study. European Heart Journal Conference: European Society of Cardiology, ESC Congress 2013;August.
(362) Milkas A, Tsioufis C, Kasiakogias A, Flessas D, Mendrinos D, Koutra E, Anastasopoulos I, Tsiachris D, Tousoulis D, Stefanadis C. Chronic kidney disease and development of atrial fibrillation in treated hypertensive patients: A 4-year prospective study. European Heart Journal Conference: European Society of Cardiology, ESC Congress 2013;August.
(363) Brugger N, Krause R, Carlen F, Rimensberger C, Hille R, Steck H, Wilhelm M, Seiler C. Effect of lifetime endurance training on left atrial mechanical function and on the risk of atrial fibrillation. European Heart Journal Conference: European Society of Cardiology, ESC Congress 2013;August.
(364) Hung C-Y, Lin C-H, Loh E-W, Ting C-T, Wu T-J. CHADS2 score, statin therapy, and risks of atrial fibrillation. American Journal of Medicine 2013;126(2):133-40.
(365) Mandyam MC, Soliman EZ, Alonso A, Dewland TA, Heckbert SR, Vittinghoff E, Cummings SR, Ellinor PT, Chaitman BR, Stocke K, Applegate WB, Arking DE, et al. The QT interval and risk of incident atrial fibrillation. Heart Rhythm 2013;10(10):1562-8.
(366) Brunner KJ, Bunch TJ, Mullin CM, May HT, Bair TL, Elliot DW, Anderson JL, Mahapatra S. Clinical predictors of risk for atrial fibrillation: implications for diagnosis and monitoring. Mayo Clin Proc 2014 Nov;89(11):1498-505.
(367) Holmqvist F, Guan N, Zhu Z, Kowey P, Allen L, Fonarow G, Hylek E, Mahaffey K, Chang P, Holmes D, Peterson E, Piccini J, et al. Obstructive sleep apnea and atrial fibrillation: Findings from orbit-AF. Journal of the American College of Cardiology Conference: 63rd Annual Scientific Session of the American College of Cardiology and i2 Summit: Innovation in Intervention, ACC 14 Washington, DC United States Conference Publication 2014;63(12).
(368) Rodriguez CJ, Soliman EZ, Alonso A, Swett K, Okin PM, Goff DC, Jr., Heckbert SR. Atrial fibrillation incidence and risk factors in relation to race-ethnicity and the population attributable fraction of atrial fibrillation risk factors: the Multi-Ethnic Study of Atherosclerosis. Ann Epidemiol 2015 Feb;25(2):71-6, 76.
(369) Sciacqua A, Perticone M, Tripepi G, Tassone EJ, Cimellaro A, Mazzaferro D, Sesti G, Perticone F. CHADS2 and CHA2DS2-VASc scores are independently associated with incident atrial fibrillation: the Catanzaro Atrial Fibrillation Project. Intern Emerg Med 2015 Oct;10(7):815-21.

(370) Emdin CA, Callender T, Cao J, Rahimi K. Effect of antihypertensive agents on risk of atrial fibrillation: a meta-analysis of large-scale randomized trials. Europace 2015 May;17(5):701-10.
(371) Johnson LSB, Juhlin T, Juul-Moller S, Hedblad B, Nilsson PM, Engstrom G. A prospective study of supraventricular activity and incidence of atrial fibrillation. Heart Rhythm 2015;12(9):1898-904.
(372) Chamberlain AM, Gersh BJ, Alonso A, Chen LY, Berardi C, Manemann SM, Killian JM, Weston SA, Roger VL. Decade-long trends in atrial fibrillation incidence and survival: A community study. American Journal of Medicine 2015;128(3):260-7.
(373) Atzema CL, Khan S, Lu H, Allard YE, Russell SJ, Gravelle MR, Klein-Geltink J, Austin PC. Cardiovascular disease rates, outcomes, and quality of care in Ontario Metis: A population-based cohort study. PLoS ONE 2015;10(3):e0121779.
(374) Chen LY, Bigger T, Hickey KT, Chen H, Lopez-Jimenez C, Banerji MA, Evans G, Fleg J, Papademetriou V, Thomas A, Woo V, Seaquist ER, et al. Effect of intensive blood pressure-lowering on incident atrial fibrillation or its electrocardiographic surrogate in the action to control cardiovascular risk in diabetes blood pressure (ACCORD BP) trial. Heart Rhythm Conference: 36th Annual Scientific Sessions of the Heart Rhythm Society, Heart Rhythm 2015;May.
(375) Johnson LS, Juhlin T, Engstrom G, Nilsson PM. Risk factor changes and incident atrial fibrillation among middle-aged men in the Malmo Preventive Project cohort. Eur Heart J Cardiovasc Pharmacother 2016 Apr;2(2):81-7.
(376) Rahman F, Yin X, Larson MG, Ellinor PT, Lubitz SA, Vasan RS, McManus DD, Magnani JW, Benjamin EJ. Trajectories of Risk Factors and Risk of New-Onset Atrial Fibrillation in the Framingham Heart Study. Hypertension 2016 Sep;68(3):597-605.
(377) Hsiao LC, Muo CH, Chou CY, Tseng CH, Chen MF, Chang KC. Chronic Osteomyelitis Is Associated With Increased Risk of New-Onset Atrial Fibrillation: Evidence From a Nationwide Cohort of 23 Million People. Can J Cardiol 2016 Dec;32(12):1388-95.
(378) Fedeli U, Avossa F, Ferroni E, Saugo M, Pengo V. Contemporary Burden of Atrial Fibrillation and Associated Mortality in Northeastern Italy. Am J Cardiol 2016 Sep 1;118(5):720-4.
(379) Briffa T, Hung J, Knuiman M, McQuillan B, Chew DP, Eikelboom J, Hankey GJ, Teng TH, Nedkoff L, Weerasooriya R, Liu A, Stobie P. Trends in incidence and prevalence of hospitalization for atrial fibrillation and associated mortality in Western Australia, 1995-2010. Int J Cardiol 2016 Apr 1;208:19-25.
(380) Ramkumar S, Yang H, Wang Y, Nolan M, Negishi K, Sanders P, Marwick TH. Relation of Functional Status to Risk of Development of Atrial Fibrillation. Am J Cardiol 2017 Feb 15;119(4):572-8.
(381) Christiansen MN, Koeber L, Torp-Pedersen C, Andersson C. Incidence and prognosis of atrial fibrillation in Denmark from 1995-2012. European Heart Journal Conference: European Society of Cardiology, ESC Congress 2017;(Supplement 1):August.
(382) Di Benedetto L, Michels G, Luben R, Khaw KT, Pfister R. Individual and combined impact of lifestyle factors on atrial fibrillation in apparently healthy men and women: The EPIC-Norfolk prospective population study. Eur J Prev Cardiol 2018 Sep;25(13):1374-83.
(383) Singh JA, Cleveland JD. Gout and the risk of incident atrial fibrillation in older adults: a study of US Medicare data. RMD Open 2018;4(2):e000712.
(384) Said MA, Verweij N, van der HP. Associations of Combined Genetic and Lifestyle Risks With Incident Cardiovascular Disease and Diabetes in the UK Biobank Study. JAMA Cardiol 2018 Aug 1;3(8):693-702.
(385) Ko D, Preis SR, Lubitz SA, McManus DD, Vasan RS, Hamburg NM, Benjamin EJ, Mitchell GF. Relation of Orthostatic Hypotension With New-Onset Atrial Fibrillation (From the Framingham Heart Study). Am J Cardiol 2018 Mar 1;121(5):596-601.
(386) Weng LC, Preis SR, Hulme OL, Larson MG, Choi SH, Wang B, Trinquart L, McManus DD, Staerk L, Lin H, Lunetta KL, Ellinor PT, et al. Genetic Predisposition, Clinical Risk

Factor Burden, and Lifetime Risk of Atrial Fibrillation. Circulation 2018 Mar 6;137(10):1027-38.
(387) Kim IS, Yang PS, Lee J, Yu HT, Kim TH, Uhm JS, Pak HN, Lee MH, Joung B. Long-term exposure of fine particulate matter air pollution and incident atrial fibrillation in the general population: A nationwide cohort study. Int J Cardiol 2019 May 15;283:178-83.
(388) Renda G, Ricci F, Patti G, Aung N, Petersen SE, Gallina S, Hamrefors V, Melander O, Sutton R, Engstrom G, Caterina RD, Fedorowski A. CHA2DS2VASc score and adverse outcomes in middle-aged individuals without atrial fibrillation. European Journal of Preventive Cardiology 2019;26(18):1987-97.
(389) Hu W-S, Hsieh M-H, Lin C-L. A novel atrial fibrillation prediction model for Chinese subjects: A nationwide cohort investigation of 682 237 study participants with random forest model. Europace 2019;21(9):1307-12.
(390) Kim YG, Han K-D, Choi J-I, Boo KY, Kim DY, Oh S-K, Lee K-N, Shim J, Kim JS, Kim Y-H. The impact of body weight and diabetes on new-onset atrial fibrillation: A nationwide population based study. Cardiovascular Diabetology 2019;18(1):128.
(391) Uhm J-S, Jin M-N, Kim I-S, Kim M, Yu HT, Kim T-H, Kim J-Y, Joung B, Pak H-N, Lee M-H. Nonspecific intraventricular conduction delay is associated with future occurrence of atrial fibrillation in patients with structurally normal heart and sinus rhythm. Journal of Arrhythmia Conference: 12th Asia Pacific Heart Rhythm Society Session, APHRS 2019;(Supplement 1):December.
(392) Borschel CS, Rubsamen N, Ojeda FM, Wild PS, Hoffmann BA, Prochaska JH, Gori T, Lackner KJ, Blankenberg S, Zeller T, Munzel T, Schnabel RB. Noninvasive peripheral vascular function and atrial fibrillation in the general population. Journal of Hypertension 2019;37(5):928-34.
(393) Sun D, Li W, Zheng W, Tan J, Zhang G. Direct bilirubin level is an independent risk factor for atrial fibrillation in thyrotoxic patients receiving radioactive iodine therapy. Nuclear medicine communications 2019;40(12):1289-94.
(394) Tanaka Y, Bundy J, Allen N, Iftekhar Uddin SM, Feldman D, Michos E, Heckbert S, Greenland P. Erectile dysfunction is independently associated with incident atrial fibrillation: The Multi-Ethnic study of Atherosclerosis (MESA). Circulation Conference: American Heart Association Scientific Sessions, AHA 2019;(Supplement 1):2019.
(395) Mehra N, DeSimone C, Hodge D, Deshmukh A, Egbe AC, Madhavan M. The relationship between atrial fibrillation and coffee. Circulation Conference: American Heart Association Scientific Sessions, AHA 2019;(Supplement 1):2019.
(396) Prasitlumkum N, Kewcharoen J, Angsubhakorn N, Chongsathidkiet P, Rattanawong P. Orthostatic hypotension is associated with new-onset atrial fibrillation: Systemic review and meta-analysis. Indian Heart J 2019 Jul;71(4):320-7.
(397) Lee SR, Choi YJ, Choi EK, Han KD, Lee E, Cha MJ, Oh S, Lip GYH. Blood Pressure Variability and Incidence of New-Onset Atrial Fibrillation: A Nationwide Population-Based Study. Hypertension 2020 Dec 16;75(2):309-15.
(398) Lee SR, Choi EK, Han KD, Lee SH, Oh S. Effect of the variability of blood pressure, glucose level, total cholesterol level, and body mass index on the risk of atrial fibrillation in a healthy population. Heart Rhythm 2020 Jul 9;17(1):12-9.
(399) Sharashova E, Wilsgaard T, Ball J, Morseth B, Gerdts E, Hopstock LA, Mathiesen EB, Schirmer H, Lochen ML. Long-term blood pressure trajectories and incident atrial fibrillation in women and men: the Tromso Study. Eur Heart J 2020 May 3;41(16):1554-62.
(400) Lip GYH, Skjoth F, Nielsen PB, Larsen TB. Evaluation of the C2HEST Risk Score as a Possible Opportunistic Screening Tool for Incident Atrial Fibrillation in a Healthy Population (From a Nationwide Danish Cohort Study). Am J Cardiol 2020 Jan 1;125(1):48-54.
(401) Park YM, Moon J, Hwang IC, Lim H, Cho B. Height is associated with incident atrial fibrillation in a large Asian cohort. Int J Cardiol 2020 Apr 1;304:82-4.

(402) Janus SE, Hajjari J, Al-Kindi S. High-sensitivity troponin and the risk of atrial fibrillation in chronic kidney disease: Results from the Chronic Renal Insufficiency Cohort Study. Heart Rhythm 2020;17(2):190-4.

(403) Chang Y, Woo HG, Park J, Lee JS, Song T-J. Improved oral hygiene care is associated with decreased risk of occurrence for atrial fibrillation and heart failure: A nationwide population-based cohort study. European Journal of Preventive Cardiology 2020;27(17):1835-45.

(404) Lee S-R, Choi E-K, Han K-D, Lee S-H, Oh S. Effect of the variability of blood pressure, glucose level, total cholesterol level, and body mass index on the risk of atrial fibrillation in a healthy population. Heart Rhythm 2020;17(1):12-9.

(405) Okin PM, Hille DA, Wachtell K, Kjeldsen SE, Julius S, Devereux RB. On-treatment HDL cholesterol predicts incident atrial fibrillation in hypertensive patients with left ventricular hypertrophy. Blood Pressure 29 (5) (pp 319-326), 2020;02.

(406) Cheng M, Wang P, Xiong L, Zeng Y, Tu X, Zhang R, Xia Y, Wu G, Wang Q, Cheng X, Xu C. SNP rs2243828 in MPO associated with myeloperoxidase level and atrial fibrillation risk in Chinese Han population. Journal of Cellular and Molecular Medicine 2020;24(17):10263-6.

(407) Harrison SL, Lane DA, Banach M, Mastej M, Lip GYH, Al-Shaer B, Andrusewicz W, ndrzejczuk-Rosa M, nusz-Gaszewska E, Baginska A, Balawajder P, Banka G, et al. Lipid levels, atrial fibrillation and the impact of age: Results from the LIPIDOGRAM2015 study. Atherosclerosis 2020;312:16-22.

(408) Patel RB, Delaney JA, Hu M, Patel H, Cheng JY, Gottdiener J, Kizer JR, Marcus GM, Turakhia MP, Deo R, Heckbert SR, Psaty BM, et al. Characterization of cardiac mechanics and incident atrial fibrillation in participants of the Cardiovascular Health Study. JCI insight 2020;10.

(409) Khan AA, Junejo RT, Thomas GN, Fisher JP, Lip GYH. Heart rate variability in patients with atrial fibrillation and hypertension. European Journal of Clinical Investigation 2020;2020.

(410) Hu W-S, Lin C-L. Prediction of new-onset atrial fibrillation for general population in Asia: A comparison of C2HEST and HATCH scores. International Journal of Cardiology 2020;313:60-3.

(411) Vavik V, Pedersen EKR, Svingen GF, Solheim E, Aakre KM, Tell GS, Nygard O, Vikenes K. Systemic Cardiac Troponin T Associated With Incident Atrial Fibrillation Among Patients With Suspected Stable Angina Pectoris. American Journal of Cardiology 127 (pp 30-35), 2020;127:30-5.

(412) Xie E, Yu R, mbale-Venkatesh B, Bakhshi H, Heckbert SR, Soliman EZ, Bluemke DA, Kawut SM, Wu CO, Nazarian S, Lima JAC. Association of right atrial structure with incident atrial fibrillation: A longitudinal cohort cardiovascular magnetic resonance study from the Multi-Ethnic Study of Atherosclerosis (MESA). Journal of Cardiovascular Magnetic Resonance 2020;22(1):36.

(413) Alzoughool F, Atoum M, bu-Awad A, Ghanma I, Halalsheh R. The rs2236609 Polymorphism Is Related to Increased Risk Susceptibility of Atrial Fibrillation. Public Health Genomics 2020;23(1-2):54-8.

(414) Imran T, Wong A, Schneeweiss S, Desai RJ. Statin Lipophilicity and the Risk of Incident Heart Failure. Cardiology (Switzerland) 2020;145(6):375-83.

(415) Tanaka M, Imano H, Kubota Y, Yamagishi K, Umesawa M, Muraki I, Cui R, Hayama-Terada M, Shimizu Y, Okada T, Ohira T, Sankai T, et al. Serum High-Sensitivity C-Reactive Protein Levels and the Risk of Atrial Fibrillation in Japanese Population: the Circulatory Risk in Communities Study. Journal of atherosclerosis and thrombosis 2020;doi: 10.5551/jat.54064.

(416) Semaan S, Dewland TA, Tison GH, Nah G, Vittinghoff E, Pletcher MJ, Olgin JE, Marcus GM. Physical activity and atrial fibrillation: Data from wearable fitness trackers. Heart Rhythm Part B 2020;17(5):842-6.

(417) Staerk L, Preis SR, Lin H, Lubitz SA, Ellinor PT, Levy D, Benjamin EJ, Trinquart L. Protein Biomarkers and Risk of Atrial Fibrillation: The FHS. Circulation: Arrhythmia and Electrophysiology 2020;96-105.
(418) Siland JE, Zwartkruis V, Geelhoed B, De Boer RA, Van G, I, van der HP, Rienstra M. Lifestyle components: Self-reported physical activity, nutritional status, sleep quality and incident atrial fibrillation. IJC Heart and Vasculature 2020;27:100492.
(419) Gerber DA, Stefanick M, Hlatky M, Yang J, Hedlin H, Haring B, Perez M. Dietary protein intake and incident atrial fibrillation in postmenopausal women from the women's health initiative. Journal of the American College of Cardiology Conference: ACC 20 Together With World Congress of Cardiology United States 2020;75 (11 Supplement 2):5.
(420) Tanaka Y, Bundy JD, Allen NB, Uddin SMI, Feldman DI, Michos ED, Heckbert SR, Greenland P. Association of Erectile Dysfunction with Incident Atrial Fibrillation: The Multi-Ethnic Study of Atherosclerosis (MESA). American Journal of Medicine 2020;133(5):613-20.
(421) Roh J-H, Lee J-H, Lee H, Yoon Y-H, Kim M, Kim Y-G, Park G-M, Park J-H, Seong I-W. Association between non-alcoholic fatty liver disease and risk of new-onset atrial fibrillation in healthy adults. Liver International 2020;40(2):338-46.
(422) Kjeldsen SE, Narkiewicz K, Burnier M, Oparil S. Intensive systolic blood pressure control and prevention of new onset atrial fibrillation in the SPRINT study: is the association really controversial? Blood Press 2020 Aug;29(4):199-201.
(423) Singleton J, Chen Y, Whalen SP, Bhave D, Beaty H, Yeboah J, Soliman Z. Effect of Intensive Blood Pressure Lowering on Incident Atrial Fibrillation: A Systematic Review and Meta-Analysis. J Atr Fibrillation 2020 Dec;13(4):2401.
(424) Pinho-Gomes AC, Azevedo L, Copland E, Canoy D, Nazarzadeh M, Remakrishnan R, Berge E, Sundstrom J, Kotecha D, Woodward M, Rahimi K. Effect of blood pressure lowering treatment on the risk of atrial fibrillation: An individual-participant data meta-analysis. European Heart Journal Conference: European Society of Cardiology Congress, ESC 2020;(SUPPL 2):November.
(425) Nishikawa T, Tanaka Y, Tada H, Tsuda T, Kato T, Usui S, Sakata K, Hayashi K, Kawashiri MA, Hashiba A, Takamura M. Association between Cardiovascular Health and Incident Atrial Fibrillation in the General Japanese Population Aged â‰¥40 Years. Nutrients 2021 Sep 15;13(9).
(426) Pinho-Gomes ACP, Azevedo L, Copland E, Canoy D, Nazarzadeh M, Ramakrishnan R, Berge E, Sundstrom J, Kotecha D, Woodward M, Rahimi K. Blood pressure lowering treatment for prevention of cardiovascular events in patients with atrial fibrillation: An individual-participant data metaanalysis. Journal of Hypertension Conference: European Meeting on Hypertension and Cardiovascular Protection and International Society of Hypertension, ESH-ISH 2021;(SUPPL 1):April.
(427) Lu Z, Tilly MJ, Aribas E, Roeters Van Lennep JE, Ikram MA, de Groot NMS, van RJ, Kavousi M. Trajectories of metabolic risk factors and risk of new-onset atrial fibrillation among men and women. European Heart Journal Conference: European Society of Cardiology Congress, ESC 2021;(SUPPL 1):October.
(428) Lu Z, Tilly MJ, Geurts S, Aribas E, Roeters van LJ, de Groot NMS, Ikram MA, van RJ, Kavousi M. Sex-specific anthropometric and blood pressure trajectories and risk of incident atrial fibrillation: the Rotterdam Study. Eur J Prev Cardiol 2022 May 5.
(429) Khurshid S, Friedman S, Reeder C, Di AP, Diamant N, Singh P, Harrington LX, Wang X, Al-Alusi MA, Sarma G, Foulkes AS, Ellinor PT, et al. ECG-Based Deep Learning and Clinical Risk Factors to Predict Atrial Fibrillation. Circulation 145(2) (pp 122-133), 2022;11.
(430) Gorecka M, McDarby G, McNicholas T, Smyth B. Lifestyle risk factors for atrial fibrillation in Ireland. European Journal of Preventive Cardiology 27(19) (pp 2092;December.

(431) Rizos I, Rigopoulos AG, Kalogeropoulos AS, Tsiodras S, Dragomanovits S, Sakadakis EA, Faviou E, Kremastinos DT. Hypertension and paroxysmal atrial fibrillation: A novel predictive role of high sensitivity C-reactive protein in cardioversion and long-term recurrence. Journal of Human Hypertension 2010;24(7):447-57.

(432) Polak JF, Pencina MJ, O'Leary DH, D'Agostino RB. Common carotid artery intima-media thickness progression as a predictor of stroke in multi-ethnic study of atherosclerosis. Stroke 2011;42(11):3017-21.

(433) Hwang H-J, Lee M-Y, Youn H-J, Oh Y-S, Rho T-H, Chung W-S, Park C-S, Choi Y-S, Chung W-B, Lee J-B, Park H-K, Lim K, et al. Association between plaque thickness of the thoracic aorta and recurrence of atrial fibrillation after ablation. Korean Circulation Journal 2011;41(4):177-83.

(434) de Vos CB, Breithardt G, Camm AJ, Dorian P, Kowey PR, Le HJY, Naditch-Brule L, Prystowsky EN, Schwartz PJ, Torp-Pedersen C, Weintraub W, Crijns HJ. Progression of atrial fibrillation in the recordaf cohort: Clinical correlates and the effect of rhythm control therapy. Heart Rhythm Conference: 32nd Annual Scientific Sessions of the Heart Rhythm Society, Heart Rhythm San Francisco, CA United States Conference Publication 2011;8 (5 SUPPL.1):S85.

(435) Mirza M, Sofi A, Mori N, Tran C, Khan U, Shen W, Jahangir A. Treatment for severe restless legs syndrome in the elderly reduces the risk of progression of trial fibrillation. Clinical Pharmacology and Therapeutics Conference: American Society for Clinical Pharmacology and Therapeutics Dallas, TX United States Conference Publication 2011;89 (SUPPL.1):S32.

(436) Tsioufis C, Kasiakogias A, Thomopoulos C, Dimitriadis K, Kordalis A, Kefala A, Hatzigiannis P. Severe resistant hypertension and incidence of cardiovascular disease. Journal of Clinical Hypertension Conference: American Society of Hypertension, Inc 27th Annual Scientific Meeting and Exposition New York, NY United States Conference Publication 2012;14 (SUPPL.1):April.

(437) Tremblay-Gravel M, White M, Roy D, Leduc H, Wyse D, Cadrin-Tourigny J, Macle L, Dubuc M, Andrade J, Rivard L, Guerra P, Thibault B, et al. Blood pressure and atrial fibrillation: A combined AF-CHF and AFFIRM analysis. Canadian Journal of Cardiology Conference: 65th Annual Meeting of the Canadian Cardiovascular Society Toronto, ON Canada Conference Publication 2012;28 (5 SUPPL.1):S290.

(438) Barrett TW, Self WH, Jenkins CA, Storrow AB, Heavrin BS, McNaughton CD, Collins SP, Goldberger JJ. Predictors of regional variations in hospitalizations following emergency department visits for atrial fibrillation. American Journal of Cardiology 2013;112(9):1410-6.

(439) Christiansen CB, Olesen JB, Gislason G, Lock-Hansen M, Torp-Pedersen C. Cardiovascular and non-cardiovascular hospital admissions associated with atrial fibrillation: a Danish nationwide, retrospective cohort study. BMJ Open 2013;3(1):e001800.

(440) Karasoy D, Torp-Pedersen C, Gislason G, Hansen J, Johannessen A, Merie C, Ozcan C, Hvidtfeldt M, Hansen ML. Risk of stroke after radiofrequency ablation of atrial fibrillation: 10-year follow-up of Danish nationwide cohort. European Heart Journal Conference: European Society of Cardiology, ESC Congress 2013;August.

(441) Sotomi Y, Inoue K, Ito N, Kimura R, Toyoshima Y, Masuda M, Iwakura K, Fujii K. Incidence and risk factors for very late recurrence of atrial fibrillation after radiofrequency catheter ablation. Europace 2013;15(11):1581-7.

(442) van der Ende MY, Hartman MHT, Hagemeijer Y, Meems LMG, de Vries HS, Stolk RP, De Boer RA, Sijtsma A, Van Der MP, Rienstra M, van der HP. The LifeLines Cohort Study: Prevalence and treatment of cardiovascular disease and risk factors. International Journal of Cardiology 2017;228:495-500.

(443) Frey MK, Richter B, Gwechenberger M, Marx M, Pezawas T, Schrutka L, Gossinger H. High incidence of atrial fibrillation after successful catheter ablation of atrioventricular nodal reentrant tachycardia: a 15.5-year follow-up. Scientific reports 2019;9(1):11784.
(444) Kim D, Yang P-S, Jang E, Tae YH, Kim T-H, Uhm J-S, Sung J-H, Pak H-N, Lee M-H, Lip G, Joung B. Effects of systolic blood pressure and hypertension duration on dementia in patients with atrial fibrillation. Journal of Arrhythmia Conference: 12th Asia Pacific Heart Rhythm Society Session, APHRS 2019;(Supplement 1):December.
(445) Rafie S, Kaveyani H, Choghakabodi PM. Risk factors associated with recurrent stroke: A retrospective hospital-based study. Journal of Acute Disease 2019;8(6):245-9.
(446) Martin-Merino E, Ruigomez A, Johansson S, Garcia-Rodriguez L-A. Hospitalised ischaemic cerebrovascular accident and risk factors in a primary care database. Pharmacoepidemiology and Drug Safety 2020;20(10):1050-6.
(447) Hiremath N, Kate M, Mohimen A, Kesavadas C, Sylaja PN. Risk factors of white matter hyperintensities in South Asian patients with transient ischemic attack and minor stroke. Neuroradiology 2020;62(10):1279-84.
(448) Gawalko M, Budnik M, Uzieblo-Zyczkowska B, Krzesinski P, Scislo P, Kochanowski J, Jurek A, Kiliszek M, Gielerak G, Filipiak KJ, Opolski G, Kaplon-Cieslicka A. Decreased left atrial appendage emptying velocity as a link between atrial fibrillation type, heart failure and older age and the risk of left atrial thrombus in atrial fibrillation. International Journal of Clinical Practice 2020;2020.
(449) Kuybu O, Amireh A, Davis D, Kelley RE, Javalkar V. Prevalence of ischemic stroke and atrial fibrillation in young patients with migraine national inpatient sample analysis. Journal of Stroke and Cerebrovascular Diseases 2020;29(8):104972.
(450) Wang YF, Lu SX, Xia SJ, Jia ZX, Jiang C, He L, Du X, Ma CS. Risk factors for recurrent hospitalizations of patients with atrial fibrillation. [Chinese]. Zhonghua xin xue guan bing za zhi 2020;48(4):308-14.
(451) Li J, Sang C, Du X, He L, Lu S, Xia S, Chang S, Zuo S, Guo X, Li S, Tang R, Liu N, et al. Effectiveness and safety of atrial fibrillation ablation in females. PACE - Pacing and Clinical Electrophysiology 2020;43(6):583-92.
(452) Shih T, Ledezma K, McCauley M, Rehman J, Galanter WL, Darbar D. Impact of traditional risk factors for the outcomes of atrial fibrillation across race and ethnicity and sex groups. IJC Heart and Vasculature 2020;28:100538.
(453) Kim D, Yang P-S, Jang E, Yu HT, Kim T-H, Uhm J-S, Kim J-Y, Sung J-H, Pak H-N, Lee M-H, Lip GYH, Joung B. Blood pressure control and dementia risk in midlife patients with atrial fibrillation. Hypertension 2020;1296-304.
(454) VanWagner LB, Holl JL, Montag S, Gregory D, Connolly S, Kosirog M, Campbell P, Pine S, Daud A, Finn D, Ladner D, Skaro AI, et al. Blood pressure control according to clinical practice guidelines is associated with decreased mortality and cardiovascular events among liver transplant recipients. American Journal of Transplantation 2020;20(3):797-807.
(455) Ergatoudes C, Hansson P-O, Svardsudd K, Rosengren A, Ostgard TE, Caidahl K, Pivodic A, Fu M. Comparison of incidence rates and risk factors of heart failure between two male cohorts born 30 years apart. Heart 2020;106(21):1672-8.
(456) Bowling CB, Sloane R, Pieper C, Luciano A, Davis BR, Simpson LM, Einhorn PT, Oparil S, Muntner P. Association of Sustained Blood Pressure Control with Multimorbidity Progression Among Older Adults. Journal of the American Geriatrics Society 2020;68(9):2059-66.
(457) Harding BN, Norby FL, Heckbert SR, McKnight B, Psaty BM, Soliman EZ, Floyd JS, Chen LY. Longitudinal Measures of Blood Pressure and Subclinical Atrial Arrhythmias: The MESA and the ARIC Study. J Am Heart Assoc 2021 Jun;10(11):e020260.
(458) Tsiachris D, Tsioufis C, Kasiakogias A, Poulakis M, Almyroudi M, Aragiannis D, Kintis K, Andrikou I, Anastasopoulos I, Stefanadis.C. Left atrium size and incidence of new-onset atrial fibrillation in essential hypertensives. A 6 years prospective study. European Heart Journal Conference: European Society of Cardiology, ESC Congress 2011;August.

(459) Ariansen I, Reims HM, Gjesdal K, Olsen MH, Ibsen H, Devereux RB, Okin PM, Kjeldsen SE, Dahlof B, Wachtell K. Impact of alcohol habits and smoking on the risk of new-onset atrial fibrillation in hypertensive patients with ECG left ventricular hypertrophy: The LIFE Study. Blood Pressure 2012;21(1):6-11.
(460) Mrdovic I, Savic L, Krljanac G, Perunicic J, Asanin M, Lasica R, Antonijevic N, Kocev N, Marinkovic J, Vasiljevic Z, Ostojic M. Incidence, predictors, and 30-day outcomes of new-onset atrial fibrillation after primary percutaneous coronary intervention: Insight into the RISK-PCI trial. Coronary Artery Disease 2012;23(1):1-8.
(461) Frontzek KJ, Fluri F, Gass A, Mueller B, Christ-Crain M, Katan M. Relationship of insular stroke on newly detected atrial fibrillation and MRproANP levels. Cerebrovascular Diseases Conference: 21st European Stroke Conference Lisbon Portugal Conference Publication 2012;33 (SUPPL.2):494-5.
(462) Ganga HV, Nair SU, Puppala VK, Miller WL. Risk of new-onset atrial fibrillation in elderly patients with the overlap syndrome: A retrospective cohort study. Journal of Geriatric Cardiology 2013;10(2):129-34.
(463) Dauriz M, Mantovani A, Pichiri I, Rigolon R, Zoppini G, Bonora E, Targher G. Non-alcoholic fatty liver disease is associated with an increased prevalence of atrial fibrillation in patients with type 2 diabetes. Diabetes Conference: 73rd Scientific Sessions of the American Diabetes Association Chicago, IL United States Conference Publication 2013;62 (SUPPL.1):A371.
(464) Bolesta S, Kong F. Effect of statin medications on the incidence of postoperative atrial fibrillation after cardiac valve surgery. Critical Care Medicine Conference: 42nd Critical Care Congress of the Society of Critical Care Medicine, SCCM 2013;December.
(465) Lee H-J, Choi E-K, Han K-D, Kim DH, Lee E, Lee S-R, Oh S, Lip GYH. High variability in bodyweight is associated with an increased risk of atrial fibrillation in patients with type 2 diabetes mellitus: A nationwide cohort study. Cardiovascular Diabetology 2019;(1):78.
(466) Bordi L, Kovacs I, Korodi SZ, Hodas R, Benedek T, Benedek I. Epicardial fat volume is associated with the risk of atrial fibrillation recurrence following pulmonary vein isolation. European Heart Journal Cardiovascular Imaging Conference: Annual Meeting of the European Association of Echocardiography, EUROECHO 2019;(Supplement 1):January.
(467) Providencia R, Adragao P, De AC, Chun J, Chierchia G, Defaye P, Anselme F, Creta A, Lambiase PD, Schmidt B, Chen S, Cavaco D, et al. Impact of Body Mass Index on the Outcomes of Catheter Ablation of Atrial Fibrillation: A European Observational Multicenter Study. Journal of the American Heart Association 2019;8(20):e012253.
(468) Saraiva RM, Pacheco NP, Pereira TOJS, Costa AR, Holanda MT, Sangenis LHC, Mendes FSNS, Sousa AS, Hasslocher-Moreno AM, Xavier SS, Mediano MFF, Veloso HH. Left Atrial Structure and Function Predictors of New-Onset Atrial Fibrillation in Patients with Chagas Disease. Journal of the American Society of Echocardiography 2020;33(11):1363-74.
(469) Tse G, Lee S, Mok NS, Liu T, Chang D. Incidence and predictors of atrial fibrillation in a Chinese cohort of Brugada syndrome. International Journal of Cardiology 2020;314:54-7.
(470) Mahfouz RA, El-Shetry M, Frere A, Safwat M. Blood Pressure Variability and Atrial Fibrillation in Patients with Acute ST Segment Elevation Myocardial Infarction: The Relation with Left Atrial Electromechanical Delay-A 1-Year Follow-Up Study. Pulse 2020;8(1-2):57-65.
(471) Bianco I, da Silva GO, Dal Forno ARJ, Nascimento HG, Lewandowski A, Pereira E, D'Avila A. Risk of atrial fibrillation after ablation of cavotricuspid isthmus-dependent atrial flutter: Is combined ablation of atrial fibrillation worthwhile? Arquivos Brasileiros de Cardiologia 2020;114(5):775-82.
(472) Kawakami H, Ramkumar S, Pathan F, Wright L, Marwick TH. Use of echocardiography to stratify the risk of atrial fibrillation: Comparison of left atrial and ventricular strain. European Heart Journal Cardiovascular Imaging 2020;21(4):399-407.

(473) Kim M, Hong M, Kim J-Y, Kim I-S, Yu HT, Kim T-H, Uhm J-S, Joung B, Lee M-H, Pak H-N. Clinical relationship between anemia and atrial fibrillation recurrence after catheter ablation without genetic background. IJC Heart and Vasculature 2020;27:100507.
(474) Soliman EZ, Howard G, Judd S, Bhave PD, Howard VJ, Herrington DM. Factors Modifying the Risk of Atrial Fibrillation Associated With Atrial Premature Complexes in Patients With Hypertension. American Journal of Cardiology 2020;125(9):1324-31.
(475) Park HE, Lee H, Choi S-Y, Kim HS, Chung GE. The risk of atrial fibrillation in patients with non-alcoholic fatty liver disease and a high hepatic fibrosis index. Scientific reports 2020;10(1):5023.
(476) Hsieh C-Y, Lee C-H, Sung S-F. Development of a novel score to predict newly diagnosed atrial fibrillation after ischemic stroke: The CHASE-LESS score. Atherosclerosis 2020;295:1-7.
(477) Axtell AL, Moonsamy P, Melnitchouk S, Tolis G, Jassar AS, D'Alessandro DA, Villavicencio MA, Cameron DE, Sundt TM. Preoperative predictors of new-onset prolonged atrial fibrillation after surgical aortic valve replacement. Journal of Thoracic and Cardiovascular Surgery 2020;159(4):1407-14.
(478) Platek AE, Szymanska A, Kalaszczynska I, Szymanski FM, Sierdzinski J, Filipiak KJ. Usefulness of Visfatin as a Predictor of Atrial Fibrillation Recurrence After Ablation Procedure. American Journal of Cardiology 2020;125(3):415-9.
(479) Park JJ, Park J-H, Hwang I-C, Park J-B, Cho G-Y, Marwick TH. Left Atrial Strain as a Predictor of New-Onset Atrial Fibrillation in Patients With Heart Failure. JACC: Cardiovascular Imaging 2020;13(10):2071-81.
(480) Tsioufis KP, Konstantinidis D, Dimitriadis K, Kasiakogias A, Liatakis I, Koutra E, Leontsinis I, Kouremeti M, Iliakis P, Karaminas N, Dimitriadi M, Tousoulis D. Isolated systolic hypertension versus combined systolic-diastolic hypertension as predictor of atrial fibrilation: Data from an 8-year-follow-up study. Journal of the American College of Cardiology Conference: ACC 20 - World Congress of Cardiology United States 2052;75(11):24.
(481) Camm AJ, Evans KE, Ward DE, Martin A. The rhythm of the heart in active elderly subjects. American Heart Journal 1980;99(5):598-603.
(482) Stewart FM, Singh Y, Persson S, Gamble GD, Braatvedt GD. Atrial fibrillation: Prevalence and management in an acute general medical unit. Australian and New Zealand Journal of Medicine 1999;29(1):51-8.
(483) Pioger G, Jauvert G, Nitzsche R, Pozzan J, Henry L, Zigelman M, Leny G, Vandrell M-C, Ritter P, Cazeau S. Incidence and predictive factors of atrial fibrillation in paced patients. PACE - Pacing and Clinical Electrophysiology 2005;28 (SUPPL.1):S137-S141.
(484) Martin-Rioboo E, Garcia CE, Perula De Torres LA, Cea-Calvo L, Anguita SM, Lopez GA, Urena FT, Garcia ML, Molina DR. Prevalence of left ventricular hypertrophy, atrial fibrillation and cardiovascular disease in hypertensive patients of Andalusia, Spain. PREHVIA study. [Spanish]. Medicina Clinica 2009;132(7):243-50.
(485) Wagdi P. Incidence and predictors of atrial fibrillation following transcatheter closure of interatrial septal communications using contemporary devices. Clinical Research in Cardiology 2010;99(8):507-10.
(486) Mahapatra S, Mason PK, Darby A, McDaniel GM, Bunch TJ. Incidence and predictors of AF after epicardial VT ablation. Heart Rhythm Conference: 31st Annual Scientific Sessions of the Heart Rhythm Society, Heart Rhythm 2010;May.
(487) Winkelmayer WC, Patrick AR, Liu J, Brookhart MA, Setoguchi S. The increasing prevalence of atrial fibrillation among hemodialysis patients. Journal of the American Society of Nephrology 2011;22(2):349-57.
(488) Valbusa F, Bonapace S, Bertolini L, Zenari L, Arcaro G, Targher G. Increased pulse pressure independently predicts incident atrial fibrillation in patients with type 2 diabetes. Diabetes Care 2012;35(11):2337-9.

(489) Olesen JB, Lip GYH, Lane DA, Kober L, Hansen ML, Karasoy D, Hansen CM, Gislason GH, Torp-Pedersen C. Vascular disease and stroke risk in atrial fibrillation: A nationwide cohort study. American Journal of Medicine 2012;125(8):826.e13-826.e23.
(490) Arias-Rivas S, Rodriguez-Yanez M, Fernandez-Pajarin G, Santamaria-Cadavid M, Castillo J, Blanco M. High serum levels of pro-brain natriuretic peptide (pro-BNP) predict the occurrence of atrial fibrillation in patients with cryptogenic stroke. Cerebrovascular Diseases Conference: 21st European Stroke Conference Lisbon Portugal Conference Publication 2012;33 (SUPPL.2):525-6.
(491) Ganga HV, Nair SU, Puppala VK, Gupta AV. Risk of new-onset atrial fibrillation in elderly patients with OSA and COPD: A community-based retrospective study. Journal of the American College of Cardiology Conference: 61th Annual Scientific Session of the American College of Cardiology and i2 Summit: Innovation in Intervention, ACC 12 Chicago, IL United States Conference Publication 2012;59(13 Suppl.):27.
(492) Shah T, Shulman E, Kargoli F, Romero J, Gross J, Kim SJ, Fisher J, Ferrick K, Krumerman A. Treatment for hypertension and atrial fibrillation in African Americans, hispanics and non-hispanic whites. Circulation Conference: Resuscitation Science Symposium, ReSS 2017;(Supplement 1):November.
(493) Giancaterino S, Nishimura M, Birgersdotter-Green U, Hoffmayer KS, Han FT, Raissi F, Ho G, Krummen D, Feld GK, Hsu JC. Clinical factors associated with baseline history of atrial fibrillation and subsequent clinical outcomes following initial implantable cardioverter-defibrillator placement. PACE - Pacing and Clinical Electrophysiology 2020;43(6):542-50.
(494) Mourtzinis G, Schioler L, Bengtsson BK, Kahan T, Hjerpe P, Hasselstrom J, Wettermark B, Manhem K. The blood pressure lowering effect on new-onset atrial fibrillation. Journal of Hypertension Conference: 26th European Meeting on Hypertension and Cardiovascular Protection, ESH 2016;(Supplement 2):September.
(495) Koracevic G, Cvetkovic E, Tomasevic M, Krstic N, Apostolovic S, Glasnovic J, Ciric S, Nagorni S, Atanaskovic V, Petrovic S. Atrial fibrillation doubled in-hospital mortality in 1397 acute cardiogenic pulmonary edema patients. Circulation Conference: World Congress of Cardiology Scientific Sessions 2010;13.
(496) Ozaydin M, Turker Y, Erdogan D, Karabacak M, Dogan A, Varol E, Gonul E, Altinbas A. The association between previous statin use and development of atrial fibrillation in patients presenting with acute coronary syndrome. International Journal of Cardiology 2010;141(2):147-50.
(497) Ne JYA, Ng ACC, Hyun K, Boroumand F, Chow CK, Freedman BB, Brieger D. Predictors of new-onset atrial fibrillation in the setting of acute coronary syndromes: A nationwide multi-center study. Circulation Conference: American Hearts Association's 2021;(SUPPL 1):November.
(498) Chinitz JS, Gerstenfeld EP, Marchlinski FE, Callans DJ. Atrial fibrillation is common after ablation of isolated atrial flutter during long-term follow-up. Heart Rhythm 2007;4(8):1029-33.
(499) Thacker EL, McKnight B, Psaty BM, Longstreth WT, Jr., Dublin S, Jensen PN, Newton KM, Smith NL, Siscovick DS, Heckbert SR. Association of body mass index, diabetes, hypertension, and blood pressure levels with risk of permanent atrial fibrillation. J Gen Intern Med 2013 Feb;28(2):247-53.
(500) Takagi D, Yamashita Y, Hamatani Y, Takabayashi K, Esato M, Chun YH, Wada H, Hasegawa K, Abe M, Akao M. Clinical characteristics of young atrial fibrillation patients: From one-year follow-up of the Fushimi AF Registry. European Heart Journal Conference: European Society of Cardiology, ESC Congress 2014;01.
(501) Tsiachris D, Tsioufis C, Dimitriadis K, Kintis K, Mazaraki A, Nikolopoulou L, Koutra E, Kefala A, Milkas T, Stefanadis C. Predictors of new-onset atrial fibrillation in essential hypertensives. Left atrial size is the one that matters. European Heart Journal Conference: European Society of Cardiology, ESC Congress 2014;01.

(502) Vanerio G. Atrial fibrillation in the young: Nothing more deceptive than an obvious fact. European Heart Journal Conference: European Society of Cardiology, ESC Congress 2014;01.
(503) Xiong Q, Shantsila A, Zhou Q, Liu Y, Shen Y, Lane D, Cheng X, Hong K, Lip GYH. Gender differences in clinical characteristics and inpatient outcomes amongst 2442 hospitalized Chinese patients with nonvalvular atrial fibrillation: The Nanchang Atrial Fibrillation Registry. Journal of the American College of Cardiology Conference: 26th Great Wall International Congress of Cardiology, Asia Pacific Heart Congress 2015;20.
(504) Szymanski FM, Filipiak KJ, Platek AE, Kotkowski M, Opolski G. Prevalence of arterial hypertension in patients with atrial fibrillation undergoing ablation. A prospective cohort study. Journal of Hypertension Conference: 25th European Meeting on Hypertension and Cardiovascular Protection, ESH 2015;June.
(505) Pokushalov E, Romanov A, Katritsis DG, Strelnikov A, Artemenko S, Losik D, Baranova V, Karaskov A, Steinberg JS, Risteski D. Relationship between reduction of systolic blood pressure and atrial fibrillation burden after renal denervation and pulmonary vein isolation. Heart Rhythm Conference: 36th Annual Scientific Sessions of the Heart Rhythm Society, Heart Rhythm 2015;May.
(506) Otomo K, Meguro T, Nakamura S. Prevalence of obesity, diabetes mellitus and hypertension in different atrial fibrillation types and perspectives on their roles in atrial fibrillation progression. Heart Rhythm Conference: 36th Annual Scientific Sessions of the Heart Rhythm Society, Heart Rhythm 2015;May.
(507) Pokushalov E, Romanov A, Katritsis D, Strelnikov A, Artyomenko S, Losik D, Baranova V, Karaskov A, Steinberg J, Risteski D. Relationship between reduction of systolic blood pressure and atrial fibrillation burden after renal denervation and pulmonary vein isolation. Journal of the American College of Cardiology Conference: 64th Annual Scientific Session of the American College of Cardiology and i2 Summit: Innovation in Intervention, ACC 15 San Diego, CA United States Conference Publication 2015;65 (10 Suppl.).
(508) Vemulapalli S, Inohara T, Kim S, Thomas L, Piccini JP, Patel MR, Chang P, Fonarow GC, Ezekowitz MD, Hylek E, Go AS, Kowey PR, et al. Blood Pressure Control and Cardiovascular Outcomes in Patients With Atrial Fibrillation (From the ORBIT-AF Registry). Am J Cardiol 2019 May 15;123(10):1628-36.
(509) Minhas JS, Coles B, Mistri AK, Eveson DJ, Hussain ST, Potter JF, Khunti K, Robinson TG. What is the optimal blood pressure level for patients with atrial fibrillation treated with direct oral anticoagulants? Journal of Hypertension 2020;38(9):1820-8.
(510) Matsumoto C, Ogawa H, Saito Y, Okada S, Soejima H, Sakuma M, Masuda I, Nakayama M, Doi N, Jinnouchi H, Waki M, Morimoto T. Association of optimal blood pressure and risk of atrial fibrillation in aged type 2 diabetes mellitus patients: From the JPAD cohort study. Circulation Conference: American Hearts Association's 2021;(SUPPL 1):November.
(511) Carrera P, Thongprayoon C, Cheungpasitporn W, Iyer V, Moua T. Epidemiology and outcomes of new-onset atrial fibrillation in the ICU: A 6-year retrospective study. Critical Care Medicine Conference: Critical Care Congress 2015;December.
(512) Ortiz M, Pardo J, Aguayo R, Nazzal C, Corbalan R. Clinical and demographic characteristics of patients with atrial fibrillation in Chile. Journal of Cardiovascular Electrophysiology Conference: Venice Arrhythmias 2009;October.
(513) Xie M, Li S-L. Analysis of postoperative new-onset atrial fibrillation in critically ill patients undergoing non-cardiac surgery. [Chinese]. Medical Journal of Chinese People's Liberation Army 2015;40(5):376-81.
(514) Mirza M, Sofi A, Tran C, Sultan S, Tachijan A, Viqar M, Mori N, Shen W-K, Jahangir A. Periodic leg movement disorder as an unrecognized risk factor for the progression of atrial fibrillation. Heart Rhythm Conference: 31st Annual Scientific Sessions of the Heart Rhythm Society, Heart Rhythm 2010;May.

(515) Chen K, Bai R, Deng W, Gao C, Wang X, Wang S, Fu H, Zhao Y, Zhang J, Dong J, Ma C. HATCH score in the prediction of new-onset atrial fibrillation after catheter ablation of typical atrial flutter. Heart Rhythm 2015;12(7):1483-9.
(516) Savvari P, Triantafyllidi H, Skiadas J, Kalogeropoulos P, Menegas D, Manolis A, Papoulidis N, Andrikopoulos G, Tsioufis K. Increased detection of suspected atrial fibrillation in elderly and female hypertensive patients through home blood pressure monitoring: the HOME-AF study. J Hypertens 2020 Nov 4;38(3):441-7.
(517) Pedersen OD, Bagger H, Kober L, Torp-Pedersen C. The occurrence and prognostic significance of atrial fibrillation/-flutter following acute myocardial infarction. European Heart Journal 1999;20(10):784-54.
(518) Pizzetti F, Turazza FM, Franzosi MG, Barlera S, Ledda A, Maggioni AP, Santoro L, Tognoni G. Incidence and prognostic significance of atrial fibrillation in acute myocardial infarction: The GISSI-3 data. Heart 2001;86(5):527-32.
(519) guiar-Souto P, Garcia Acuna JM, gra Bemejo RM, Raposeiras RS, Jacquet-Hervet M, bu-Assi E, Gonzalez-Juanatey JR. Acute atrial fibrillation after acute myocardial infarction involves similar prognosis relevance than chronic atrial fibrillation regarding mortality and heart failure development. European Heart Journal Conference: European Society of Cardiology, ESC Congress 2010;September.
(520) Banach M, Goch A, Misztal M, Rysz J, Jaszewski R, Goch JH. Predictors of paroxysmal atrial fibrillation in patients undergoing aortic valve replacement. Journal of Thoracic and Cardiovascular Surgery 2007;134(6):1569-76.
(521) Zuo M-L, Liu S, Chan K-H, Lau K-K, Chong B-H, Lam K-F, Chan Y-H, Lau Y-F, Lip GYH, Lau C-P, Tse H-F, Siu C-W. The CHADS2 and CHA2DS2-VASc scores predict new occurrence of atrial fibrillation and ischemic stroke. Journal of Interventional Cardiac Electrophysiology 2013;37(1):47-54.
(522) El-Chami MF, Kilgo P, Delurgio DB, Leon AR, Puskas JD. A risk index for the prediction of new onset atrial fibrillation after cardiac revascularization surgery. Heart Rhythm Conference: 30th Annual Scientific Sessions of the Heart Rhythm Society, Heart Rhythm 2009;May.
(523) Ong BH, Ho WY, Liew R, Chua YL. Retrospective study on atrial fibrillation post-coronary artery bypass graft surgery. Proceedings of Singapore Healthcare Conference: SingHealth DukeNUS Scientific Congress 2010;June.
(524) Kinoshita T, Asai T, Suzuki T, Kambara A, Matsubayashi K. Preoperative C-reactive protein and atrial fibrillation after isolated off-pump coronary artery bypass grafting. European Heart Journal Conference: European Society of Cardiology, ESC Congress 2010;September.
(525) Janstk D, Silva R, Pardini E, Oliveira D, Egito E, Barbosa M, Souza L, Jatene A, Peigas L. Current in-hospital prevalence of atrial fibillation after coronary artery bypass graft. Circulation Conference: World Congress of Cardiology Scientific Sessions 2010;13.
(526) Tadic M, Ivanovic B, Zivkovic N. Predictors of atrial fibrillation following coronary artery bypass surgery. Med Sci Monit 2011 Jan;17(1):CR48-CR55.
(527) Rogers CA, Angelini GD, Culliford LA, Capoun R, Ascione R. Coronary Surgery in Patients With Preexisting Chronic Atrial Fibrillation: Early and Midterm Clinical Outcome. Annals of Thoracic Surgery 2006;81(5):1676-82.
(528) Melina G, Angeloni E, Di NG, Benedetto U, Fiorani B, Sclafani G, Comito C, Tonelli E, Refice S, Sinatra R. Preoperative HMG-CoA reductase inhibitors and atrial fibrillation in patients undergoing CABG: A prospective randomised trial. European Heart Journal Conference: European Society of Cardiology, ESC Congress 2009;September.
(529) Lahiri MK, Fang K, Lamerato L, Khan A, Schuger CD. Incidence of postoperative atrial fibrillation is markedly reduced in african americans compared to whites. Heart Rhythm Conference: 31st Annual Scientific Sessions of the Heart Rhythm Society, Heart Rhythm 2010;May.

(530) Ergunes K, Yurekli I, Yasa H, Bademci M, Yazman S, Yetkin U, Yilik L, Gurbuz A. Factors affecting the incidence of atrial fibrillation after isolated on-pump coronary artery bypass and effect of atrial fibrillation on postoperative morbidity and mortality. Heart Surgery Forum Conference: 2020;October.

(531) El-Chami M, Merchant F, Smith P, Levy M, Merlino J, Leon A. Risk of post CABG atrial fibrillation recurrence using an implantable loop monitor: The monitor-AF study. Journal of the American College of Cardiology Conference: 65th Annual Scientific Session of the American College of Cardiology and i2 Summit: Innovation in Intervention, ACC 16 Chicago, IL United States Conference Publication 2016;67 (13 Suppl.):05.

(532) Aranki SF, Shaw DP, Adams DH, Rizzo RJ, Couper GS, VanderVliet M, Collins J, Cohn LH, Burstin HR. Predictors of atrial fibrillation after coronary artery surgery: Current trends and impact on hospital resources. Circulation 1996;94(3):390-7.

(533) Miceli A, Fino C, Fiorani B, Yeatman M, Narayan P, Angelini GD, Caputo M. Effects of Preoperative Statin Treatment on the Incidence of Postoperative Atrial Fibrillation in Patients Undergoing Coronary Artery Bypass Grafting. Annals of Thoracic Surgery 2009;87(6):1853-8.

(534) Ninni S, Lemesle G, Meurice T, Tricot O, Lamblin N, Bauters C. Real-Life Incident Atrial Fibrillation in Outpatients with Coronary Artery Disease. J Clin Med 2020 Jul 24;9(8).

(535) Funk M, Richards SB, Desjardins J, Bebon C, Wilcox H. Incidence, timing, symptoms, and risk factors for atrial fibrillation after cardiac surgery. American journal of critical care : an official publication, American Association of Critical-Care Nurses 2003;12(5):424-33.

(536) Auer J, Weber T, Berent R, Ng C-K, Lamm G, Eber B. Risk factors of postoperative atrial fibrillation after cardiac surgery. Journal of Cardiac Surgery 2005;20(5):425-31.

(537) Ahmed I, Almquist AK, Henry TD, Flavin TF, Eales F, Kshettry VR, Kroshus TJ. Obesity and pulmonary hypertension are independent risk factors for postoperative paroxysmal atrial fibrillation after heart valve replacement surgery. Heart Rhythm Conference: 31st Annual Scientific Sessions of the Heart Rhythm Society, Heart Rhythm 2010;May.

(538) Yin L, Ling X, Zhang Y, Shen H, Min J, Xi W, Wang J, Wang Z. CHADS2 and CHA2DS2-VASc scoring systems for predicting atrial fibrillation following cardiac valve surgery. PLoS One 2015;10(4):e0123858.

(539) Barbara D, Rehfeldt K, Pulido J, Li Z, White R, Schaff H, Mauermann W. Diastolic function and new-onset atrial fibrillation following cardiac surgery. Annals of Cardiac Anaesthesia 2015;18(1):8-14.

(540) Stefano PL, Bugetti M, Del MG, Popescu G, Pieragnoli P, Ricciardi G, Perrotta L, Checchi L, Rondine R, Bevilacqua S, Fumagalli C, Marchionni N, et al. Overweight and aging increase the risk of atrial fibrillation after cardiac surgery independently of left atrial size and left ventricular ejection fraction. J Cardiothorac Surg 2020 Oct 15;15(1):316.

(541) Mann B, Ray R, Goldberger AL. Atrial fibrillation in congestive cardiomyopathy: Echocardiographic and hemodynamic correlates. Catheterization and Cardiovascular Diagnosis 1981;7(4):387-95.

(542) Hossein AG, Schowalter T, Nicolosi AC, Aggarwal A, Moritz TE, Henderson WG, Tarazi R, Laurie SA, Sethi GK, Grover FL, Hammermeister KE. Atrial fibrillation after cardiac surgery: A major morbid event? Annals of Surgery 1997;226(4):501-13.

(543) Sliwa K, Carrington MJ, Klug E, Opie L, Lee G, Ball J, Stewart S. Predisposing factors and incidence of newly diagnosed atrial fibrillation in an urban African community: insights from the Heart of Soweto Study. Heart 2010 Dec;96(23):1878-82.

(544) Boskovic A, Vukmirovic M, Nikolic G, Ratkovic M, Vujosevic S, Medenica M. Is hypertension a risk factor for new onset atrial fibrillation in patients with acute coronary syndrome? Journal of Hypertension Conference: 25th European Meeting on Hypertension and Cardiovascular Protection, ESH 2015;June.

(545) Brock MA, Coppola JA, Reid J, Moguillansky D. Atrial fibrillation in adults with congenital heart disease following cardiac surgery in a single center: Analysis of incidence and risk factors. Congenit Heart Dis 2019 Oct 21;14(6):924-30.

(546) Shanafelt TD, Chaffee KG, Call TG, Parikh SA, Schwager SM, Ding W, Leis JF, Chanan-Khan A, Slager SL, Kay NE. Atrial fibrillation in patients with chronic lymphocytic leukemia (CLL). Blood Conference: 57th Annual Meeting of the American Society of Hematology, ASH 2015;03.
(547) Chen M, Feng X, Sun J, Wang Q, Zhang P, Wang J, Li Y-G. Risk factors responsible for atrial fibrillation development between symptomatic patients with concealed or manifest atrioventricular accessory pathways. IJC Heart and Vasculature 2015;7:69-75.
(548) Liao KM, Chen CY. Incidence and risk factors of atrial fibrillation in Asian COPD patients. Int J Chron Obstruct Pulmon Dis 2017;12:2523-30.
(549) Siu C-W, Tung H-M, Chu K-W, Jim M-H, Lau C-P, Tse H-F. Prevalence and predictors of new-onset atrial fibrillation after elective surgery for colorectal cancer. PACE - Pacing and Clinical Electrophysiology 2005;28 (SUPPL.1):S120-S123.
(550) Gonzalez Juanatey JR, Alegria EE, Lozano Vidal JV, Llisterri Caro JL, Garcia Acuna JM, Gonzalez M, I. The role of high blood pressure in cardiac diseases in Spain. The CARDIOTENS study 1999. [Spanish]. Revista espanola de cardiologia 2001;54(2):139-49.
(551) Zethelius B, Gudbjornsdottir S, Eliasson B, Eeg-Olofsson K, Svensson A-M, Cederholm J. Risk factors for atrial fibrillation in type 2 diabetes: report from the Swedish National Diabetes Register (NDR). Diabetologia 2015;58(10):2259-68.
(552) Ding X, Zheng X, Xing A, Wang D, Qi S, Wu Y, Li H, Wu S, Hong J. High risk factors of atrial fibrillation in type 2 diabetes: results from the Chinese Kailuan study. QJM 2015 Nov;108(11):885-90.
(553) Hallstrom S, Pivodic A, Rosengren A, Olafsdottir AF, Svensson AM, Lind M. Risk Factors for Atrial Fibrillation in People With Type 1 Diabetes: An Observational Cohort Study of 36,258 Patients From the Swedish National Diabetes Registry. Diabetes Care 2019 Aug;42(8):1530-8.
(554) Otake S, Sato A, Babazono T. Prevalence and predictors of atrial fibrillation in Japanese patients with type 2 diabetes. Diabetol Int 2022 Jan;13(1):101-7.
(555) Abbott KC, Trespalacios FC, Taylor AJ, Agodoa LY. Atrial fibrillation in chronic dialysis patients in the United states: Risk factors for hospitalization and mortality. BMC Nephrology 2003;4:1-10.
(556) Chao T-F, Leu H-B, Huang C-C, Chen J-W, Chan W-L, Lin S-J, Chen S-A. Thiazolidinediones can prevent new onset atrial fibrillation in patients with non-insulin dependent diabetes. International Journal of Cardiology 2012;156(2):199-202.
(557) Yang P, Zhao Y, Wong ND. Development of a Risk Score for Atrial Fibrillation in Adults With Diabetes Mellitus (from the ACCORD Study). Am J Cardiol 2020 Jun 1;125(11):1638-43.
(558) Guazzi M, Belletti S, Bianco E, Lenatti L, Guazzi MD. Endothelial dysfunction and exercise performance in lone atrial fibrillation or associated with hypertension or diabetes: Different results with cardioversion. American Journal of Physiology - Heart and Circulatory Physiology 2006;291(2):H921-H928.
(559) Chen LY, Bigger JT, Hickey KT, Chen H, Lopez-Jimenez C, Banerji MA, Evans G, Fleg JL, Papademetriou V, Thomas A, Woo V, Seaquist ER, et al. Effect of Intensive Blood Pressure Lowering on Incident Atrial Fibrillation and P-Wave Indices in the ACCORD Blood Pressure Trial. Am J Hypertens 2016 Nov 1;29(11):1276-82.
(560) Zethelius B, Gudbjornsdottir S, Eliasson B, Eeg-Olofsson K, Svensson A-M, Cederholm J. Risk factors for atrial fibrillation in type 2 diabetes. Report from the Swedish National Diabetes Register: NDR. Diabetologia Conference: 50th Annual Meeting of the European Association for the Study of Diabetes, EASD 2014;September.
(561) Oldgren J, Healey JS, Ezekowitz M, Commerford P, Avezum A, Pais P, Zhu J, Jansky P, Sigamani A, Morillo CA, Liu L, Damasceno A, et al. Variations in cause and management of atrial fibrillation in a prospective registry of 15 400 emergency department patients in 46 countries: The RE-LY atrial fibrillation registry. Circulation 2014;129(15):1568-76.

(562) Thiruganasambandamoorthy V, Kwong K, Stiell IG, Swampillai J, Toarta C, Sumner G, Kuriachan VP, Mukarram M, Kim S, Taljaard M, Hazra S, Wells GA, et al. Short-term risk of arrhythmias among emergency department syncope patients with non-sinus rhythm. Canadian Journal of Emergency Medicine Conference: 2015;(Supplement 2):May.
(563) Atar I, Konas D, Acikel S, Kulah E, Atar A, Bozbas H, Gulmez O, Sezer S, Yildirir A, Ozdemir N, Muderrisoglu H, Ozin B. Frequency of atrial fibrillation and factors related to its development in dialysis patients. International Journal of Cardiology 2006;106(1):47-51.
(564) Liao J-N, Chao T-F, Liu C-J, Wang K-L, Chen S-J, Lin Y-J, Chang S-L, Lo L-W, Hu Y-F, Tuan T-C, Chung F-P, Chen T-J, et al. Incidence and risk factors for new-onset atrial fibrillation among patients with end-stage renal disease undergoing renal replacement therapy. Kidney International 2015;87(6):1209-15.
(565) Saeed O, Jermyn R, Kargoli F, Madan S, Mannem S, Gunda S, Nucci C, Farooqui S, Hassan S, McLarty A, Bloom M, Zolty R, et al. Blood Pressure and Adverse Events during Continuous Flow Left Ventricular Assist Device Support. Circulation: Heart Failure 2015;8(3):551-6.
(566) De SJ, Pardaens S, Audenaert T, Weytjens C, Vande KB, Willems A-M, De Laet N, Van CG. Clinical characteristics and short-term outcome of patients admitted with heart failure in Belgium: Results from the BIO-HF registry. Acta Cardiologica 2015;70(4):375-85.
(567) Conde-Martel A, Arkuch ME, Formiga F, Manzano-Espinosa L, ramburu-Bodas O, Gonzalez-Franco A, vila-Ramos MF, Suarez-Pedreira I, Herrero-Domingo A, Montero-PeRez-Barquero M. Gender related differences in clinical profile and outcome of patients with heart failure. Results of the RICA Registry. [Spanish]. Revista Clinica Espanola 2015;215(7):363-70.
(568) Mesquita D, Sousa C, Santos L, Almeida H, Almeida M, Tavares J. Atrial fibrillation in heart failure: Does maintaining sinus rhythm in acute heart failure matters? European Heart Journal: Acute Cardiovascular Care Conference: Acute Cardiovascular Care 2015;October.
(569) Gunes H, Sokmen A, Kaya H, Gungor O, Kerkutluoglu M, Guzel FB, Sokmen G. Evaluation of Atrial Electromechanical Delay to Predict Atrial Fibrillation in Hemodialysis Patients. Medicina (Kaunas) 2018 Aug 25;54(4):58.
(570) Chang TI, Liu S, Airy M, Niu J, Turakhia MP, Flythe JE, Montez-Rath ME, Winkelmayer WC. Blood Pressure and Incident Atrial Fibrillation in Older Patients Initiating Hemodialysis. Clin J Am Soc Nephrol 2019 Jul 5;14(7):1029-38.
(571) Davis RC, Hobbs FDR, Kenkre JE, Roalfe AK, Iles R, Lip GYH, Davies MK. Prevalence of atrial fibrillation in the general population and in high-risk groups: The ECHOES study. Europace 2012;14(11):1553-9.
(572) Bohm M, Schumacher H, Linz D, Reil J, Ukena C, Lonn E, Teo K, Sliwa K, Schmieder RE, Sleight P, Yusuf S. Low resting heart rates are associated with new-onset atrial fibrillation in patients with vascular disease: Results of the ONTARGET/TRANSCEND studies. Journal of Internal Medicine 2015;278(3):303-12.
(573) Cabrera S, Valles E, Benito B, Alcalde O, Jimenez J, Fan R, Marti-Almor J. Simple predictors for new onset atrial fibrillation. Int J Cardiol 2016 Oct 15;221:515-20.
(574) Okubo Y, Nakano Y, Ochi H, Onohara Y, Tokuyama T, Motoda C, Amioka M, Hironobe N, Okamura S, Ikeuchi Y, Miyauchi S, Chayama K, et al. Predicting atrial fibrillation using a combination of genetic risk score and clinical risk factors. Heart Rhythm 2020 May;17(5 Pt A):699-705.
(575) Feliz V, Saiyad S, Ramarao SM, Khan H, Guglin M. Melphalan induced atrial arrhythmias: Incidence and risk factors. Journal of the American College of Cardiology Conference: American College of Cardiology's 59th Annual Scientific Session and i2 Summit: Innovation in Intervention Atlanta, GA United States Conference Publication 2010;55 (10 SUPPL 1):A9.
(576) Pierdomenico SD, Lapenna D, Cuccurullo F. Risk of atrial fibrillation in dipper and nondipper sustained hypertensive patients. Blood Pressure Monitoring 193;13(4):August.

(577) Korostovtseva L, Sviryaev Y, Zvartau N, Rotar O, Konradi A, Shlyakhto E. The incidence of atrial fibrillation in hypertensive patients: The role of obstructive sleep apnea. Journal of Sleep Research Conference: 22nd Congress of the European Sleep Research Society Tallinn Estonia Conference Publication 207;23 (SUPPL.1):September.
(578) Ciaroni S, Cuenoud L, Bloch A. Clinical study to investigate the predictive parameters for the onset of atrial fibrillation in patients with essential hypertension. American Heart Journal 2000;139(5):814-9.
(579) Badran HM, Eid MAE, Michael A. Doppler-derived indexes and B-type natriuretic peptide in prediction of paroxysmal atrial fibrillation in essential hypertension: A prospective study. Echocardiography 2007;24(9):911-22.
(580) Vlcek M, Bur A, Woisetschlager C, Herkner H, Laggner AN, Hirschl MM. Association between hypertensive urgencies and subsequent cardiovascular events in patients with hypertension. Journal of Hypertension 2008;26(4):657-62.
(581) Ariansen I, Wachtell K, Hecht OM, Ibsen H, Devereux RB, Okin PM, Kjeldsen SE, Dahlof B. Impact of alcohol and smoking habits on the risk of new-onset atrial fibrillation in hypertensive patients with ecg left ventricular hypertrophy: the life study. European Heart Journal Conference: European Society of Cardiology, ESC Congress 2009;September.
(582) Cabrera Sole RM, Lucas CT, Fernandez R, Gonzalez JC, Landa RG, Masia J, Martinez LP. Risk factors to develop atrial fibrillation in patients with well controlled hypertension. Prospective study. Journal of Clinical Hypertension Conference: 24th Annual Scientific Meeting and Exposition of the American Society of Hypertension, ASH San Francisco, CA United States Conference Publication 2009;11 (4 SUPPL.1):A96-A97.
(583) Haywood LJ, Ford CE, Crow RS, Davis BR, Massie BM, Einhorn PT, Williard A. Atrial Fibrillation at Baseline and During Follow-Up in ALLHAT (Antihypertensive and Lipid-Lowering Treatment to Prevent Heart Attack Trial). Journal of the American College of Cardiology 2009;54(22):2023-31.
(584) Schaer BA, Schneider C, Jick SS, Conen D, Osswald S, Meier CR. Risk for incident atrial fibrillation in patients who receive antihypertensive drugs: a nested case-control study. Ann Intern Med 2010 Jan 19;152(2):78-84.
(585) Hu Y-F, Chen S-A, Yeh H-I. Metabolic syndrome does not impose greater atrial fibrillation risk in elderly hypertensive patients. Acta Cardiologica 2010;65(6):653-9.
(586) Scheltens T, De Beus MF, Hoes AW, Rutten FH, Numans ME, Mosterd A, Kors JA, Grobbee DE, Bots ML. The potential yield of ECG screening of hypertensive patients: The Utrecht Health Project. Journal of Hypertension 2010;28(7):1527-33.
(587) Morillas P, Pallares V, Llisterri JL, Sanchis C, Sanchez T, Facila L, Perez-Alonso M, Castillo J, Redon J, Bertomeu V. Prevalence of atrial fibrillation and use of antithrombotics in hypertensive patients aged >=65 years. The FAPRES trial. [Spanish]. Revista espanola de cardiologia 2010;63(8):943-50.
(588) Horio T, Iwashima Y, Kamide K, Tokudome T, Yoshihara F, Nakamura S, Kawano Y. Chronic kidney disease as an independent risk factor for new-onset atrial fibrillation in hypertensive patients. Journal of Hypertension 2010;28(8):1738-44.
(589) Liu T, Zhang X, Korantzopoulos P, Wang S, Li G. Serum uric acid is an independent risk factor for atrial fibrillation in hypertensive patients. Heart Rhythm Conference: 31st Annual Scientific Sessions of the Heart Rhythm Society, Heart Rhythm 2010;May.
(590) Vyssoulis G, Karpanou E, Adamopoulos D, Kyvelou S-M, Tzamou V, Stefanadis C. Metabolic syndrome and atrial fibriallation in essential hypertensive patients. European Heart Journal Conference: European Society of Cardiology, ESC Congress 2010;September.
(591) Vyssoulis G, Karpanou E, Adamopoulos D, Kyvelou S-M, Tzamou V, Michaelidis A, Stefanadis C. Metabolic syndrome and atrial fibrillation in patients with essential hypertension. Nutrition, Metabolism and Cardiovascular Diseases 2013;23(2):109-14.
(592) Okin PM, Hille DA, Larstorp ACK, Wachtell K, Kjeldsen SE, Dahlof B, Devereux RB. Lower achieved systolic pressure (=130 mm hg) is associated with a decreased risk of new

atrial fibrillation in hypertensive patients with electrocardiographic left ventricular hypertrophy: The life study. Circulation Conference: American Heart Association 2013;26.

(593) Jong GP, Chen HY, Li SY, Liou YS. Long-term effect of antihypertensive drugs on the risk of new-onset atrial fibrillation: a longitudinal cohort study. Hypertens Res 2014 Oct;37(10):950-3.

(594) Marott SC, Nielsen SF, Benn M, Nordestgaard BG. Antihypertensive treatment and risk of atrial fibrillation: a nationwide study. Eur Heart J 2014 May;35(18):1205-14.

(595) Alves-Cabratosa L, Garcia-Gil M, Comas-Cufi M, Ponjoan A, Marti R, Parramon D, Blanch J, Ramos R. Incident atrial fibrillation hazard in hypertensive population: a risk function from and for clinical practice. Hypertension 2015 Jun;65(6):1180-6.

(596) Losi M-A, Izzo R, De MM, Canciello G, Rapacciuolo A, Trimarco V, Stabile E, Rozza F, Esposito G, De LN, de SG, Trimarco B. Cardiovascular ultrasound exploration contributes to predict incident atrial fibrillation in arterial hypertension: The Campania Salute Network. International Journal of Cardiology 2015;199:290-5.

(597) Okin PM, Hille DA, Larstorp ACK, Wachtell K, Kjeldsen SE, Dahlof B, Devereux RB. Effect of Lower On-Treatment Systolic Blood Pressure on the Risk of Atrial Fibrillation in Hypertensive Patients. Hypertension 2015;66(2):368-73.

(598) Hsieh YC, Hung CY, Li CH, Liao YC, Huang JL, Lin CH, Wu TJ. Angiotensin-Receptor Blocker, Angiotensin-Converting Enzyme Inhibitor, and Risks of Atrial Fibrillation: A Nationwide Cohort Study. Medicine (Baltimore) 2016 May;95(20):e3721.

(599) Mourtzinis G, Schioler L, Kahan T, Bengtsson BK, Hjerpe P, Hasselstrom J, Manhem K. Antihypertensive control and new-onset atrial fibrillation: Results from the Swedish Primary Care Cardiovascular Database (SPCCD). Eur J Prev Cardiol 2017 Jul;24(11):1206-11.

(600) Konstantinidis D, Tsioufis C, Dimitriadis K, Kasiakogias A, Kouremeti M, Kalos T, Leontsinis I, Kintis K, Liatakis I, Koutra D, Tousoulis D. Isolated systolic hypertension versus combined systolic-diastolic hypertension as predictors of atrial fibrillation: Data from a Greek 8-year-follow-up study. European Heart Journal Conference: European Society of Cardiology, ESC Congress 2017;(Supplement 1):August.

(601) Larstorp ACK, Stokke IM, Kjeldsen SE, Hecht OM, Okin PM, Devereux RB, Wachtell K. Antihypertensive therapy prevents new-onset atrial fibrillation in patients with isolated systolic hypertension: the LIFE study. Blood Press 2019 Oct;28(5):317-26.

(602) Rapacciuolo A, Mancusi C, Canciello G, Izzo R, Strisciuglio T, De LN, Ammirati G, de SG, Trimarco B, Losi MA. CHA2DS2-VASc score and left atrial volume dilatation synergistically predict incident atrial fibrillation in hypertension: an observational study from the Campania Salute Network registry. Sci Rep 2019 May 27;9(1):7888.

(603) Chen C, Liu L, Yu Y, Shen G, Huang J, Huang Y, Lo K, Tang S, Feng Y. Association of systolic blood pressure with atrial fibrillation among treated hypertensive patients. Ann Palliat Med 2020 Jul;9(4):1752-63.

(604) Bhalia S, Pallangyo P, Dalidali A, Salum S, Kawajika R, Kajuna E, Kusiima H, Kifai E, Kisenge P, Waane T, Janabi M. Burden and correlates of atrial fibrillation among hypertensive patients attending a tertiary hospital in Tanzania. BMC Cardiovasc Disord 2020 May 19;20(1):235.

(605) Muria-Subirats E, Clua-Espuny JL, Ballesta-Ors J, Lorman-Carbo B, Lechuga-Duran I, Fernandez-Saez J, Pla-Farnos R, On Behalf Members Of Africat Group. Incidence and Risk Assessment for Atrial Fibrillation at 5 Years: Hypertensive Diabetic Retrospective Cohort. Int J Environ Res Public Health 2020 May 16;17(10):3491.

(606) Parcha V, Patel N, Kalra R, Kim J, Gutierrez OM, Arora G, Arora P. Incidence and Implications of Atrial Fibrillation/Flutter in Hypertension: Insights From the SPRINT Trial. Hypertension 2020 Jun;75(6):1483-90.

(607) Soliman EZ, Rahman AF, Zhang ZM, Rodriguez CJ, Chang TI, Bates JT, Ghazi L, Blackshear JL, Chonchol M, Fine LJ, Ambrosius WT, Lewis CE. Effect of Intensive Blood Pressure Lowering on the Risk of Atrial Fibrillation. Hypertension 2020 Jun;75(6):1491-6.

(608) Orozco-Beltran D, Quesada JA, Bertomeu-Gonzalez V, Lobos-Bejarano JM, Navarro-Perez J, Gil-Guillen VF, Garcia OL, Lopez-Pineda A, Castellanos-Rodriguez A, Lopez-Domenech A, Cardona-Llorens AFJ, Carratala-Munuera C. A new risk score to assess atrial fibrillation risk in hypertensive patients (ESCARVAL-RISK Project. Sci Rep 2020 Mar 16;10(1):4796.
(609) Belu E, Bataiosu C, Musetescu R, Ionescu DD, Cosulschi M, Florescu N. The incidence of atrial fibrillation in hypertensive patients with pacing. Journal of Hypertension Conference: 2020;June.
(610) Coccina F, Pierdomenico AM, De RM, Lorenzo B, Foglietta M, Petrilli I, Vitulli P, Pizzicannella J, Trubiani O, Cipollone F, Renda G, Pierdomenico SD. Risk of Atrial Fibrillation in Masked and White Coat Uncontrolled Hypertension. Am J Hypertens 2021 May 22;34(5):504-10.
(611) Coccina F, Pierdomenico AM, Ianni U, De RM, De LA, Pirro D, Pizzicannella J, Trubiani O, Cipollone F, Renda G, Pierdomenico SD. Ambulatory blood pressure and risk of new-onset atrial fibrillation in treated hypertensive patients. J Clin Hypertens (Greenwich) 2021 Jan;23(1):147-52.
(612) Bansal A, Chaudhary R, Gupta R, Gupta BS, Joad SHK, Jakhar P. Risk factors for cardiac arrhtymias in a medical ICU. Indian Journal of Critical Care Medicine Conference: 21st Annual Conference of Indian Society of Critical Care Medicine, ISCCM 2015;(13 Supplement 1):March.
(613) Svedjeholm R, Hakanson E. Predictors of atrial fibrillation in patients undergoing surgery for ischemic heart disease. Scandinavian Cardiovascular Journal 2000;34(5):516-21.
(614) Scheitz JF, Erdur H, Haeusler KG, Audebert HJ, Roser M, Laufs U, Endres M, Nolte CH. Insular Cortex Lesions, Cardiac Troponin, and Detection of Previously Unknown Atrial Fibrillation in Acute Ischemic Stroke. Stroke 2015;46(5):1196-201.
(615) Walsh SR, Oates JE, Anderson JA, Blair SD, Makin CA, Walsh CJ. Postoperative arrhythmias in colorectal surgical patients: Incidence and clinical correlates. Colorectal Disease 2006;8(3):212-6.
(616) Nicolau-Raducu R, Gitman M, Ganier D, Loss GE, Cohen AJ, Patel H, Girgrah N, Sekar K, Nossaman B. Adverse cardiac events after orthotopic liver transplantation: A cross-sectional study in 389 consecutive patients. Liver Transplantation 2015;21(1):13-21.
(617) Cardinale D, Martinoni A, Cipolla CM, Civelli M, Lamantia G, Fiorentini C, Mezzetti M. Atrial fibrillation after operation for lung cancer: Clinical and prognostic significance. Annals of Thoracic Surgery 1999;68(5):1827-31.
(618) Mason DP, Marsh DH, Alster JM, Murthy SC, McNeill AM, Budev MM, Mehta AC, Pettersson GB, Blackstone EH. Atrial Fibrillation After Lung Transplantation: Timing, Risk Factors, and Treatment. Annals of Thoracic Surgery 2007;84(6):1878-84.
(619) Lee G, Wu H, Kalman JM, Esmore D, Williams T, Snell G, Kistler PM. Atrial fibrillation following lung transplantation: Double but not single lung transplant is associated with long-term freedom from paroxysmal atrial fibrillation. European Heart Journal 2010;31(22):2774-82.
(620) Ivanovic B, Tadic M, Bradic Z, Zivkovic N, Stanisavljevic D, Celic V. The influence of the metabolic syndrome on atrial fibrillation occurrence and outcome after coronary bypass surgery: a 3-year follow-up study. Thorac Cardiovasc Surg 2014 Oct;62(7):561-8.
(621) Sorigue M, Gual-Capllonch F, Garcia O, Sarrate E, Franch-Sarto M, Ibarra G, Grau J, Orna E, Ribera J-M, Sancho J-M. Incidence, predictive factors, management, and survival impact of atrial fibrillation in non-Hodgkin lymphoma. Annals of Hematology 2018;97(9):1633-40.
(622) Erdogan A, Parahuleva M, Schaefer S, Guettler N, Neuhof C, Akcay B, Bilgin M, Mayer K, Reichenberger F, Schulz R. Prevalence of atrial fibrillation in obstructive sleep apnea. Somnologie 2009;13(4):211-4.

(623) Cadby G, McArdle N, Briffa T, Hillman DR, Simpson L, Knuiman M, Hung J. Severity of OSA is an independent predictor of incident atrial fibrillation hospitalization in a large sleep-clinic cohort. Chest 2015;148(4):945-52.
(624) Kosmala W, Saito M, Kaye G, Negishi K, Linker N, Gammage M, Marwick TH. Incremental value of left atrial structural and functional characteristics for prediction of atrial fibrillation in patients receiving cardiac pacing. Circulation: Cardiovascular Imaging 2015;8(4):e002942.
(625) Hai J, Chan P-H, Tse H-F, Siu C-W. Slow Heart Rate Predicts New Occurrence of Atrial Fibrillation. Heart Lung and Circulation 2015;24(11):1087-93.
(626) Li DL, Quispe R, Madan N, Zhang L, Taub CC. A risk score for predicting atrial fibrillation in individuals with preclinical diastolic dysfunction: a retrospective study in a single large urban center in the United States. BMC Cardiovasc Disord 2019 Feb 27;19(1):47.
(627) Park J, Yang P-S, Kim T-H, Uhm J-S, Kim J-Y, Joung B, Lee M-H, Hwang C, Pak H-N. Low left atrial compliance contributes to the clinical recurrence of atrial fibrillation after catheter ablation in patients with structurally and functionally normal heart. PLoS ONE 2015;10(12):e0143853.
(628) Donnellan E, Wolski K, Menon V, Kanj M, Wazni O, Nissen S, Tarakji K. Atrial fibrillation incidence, prevalence, and risk factors in the precision trial: A cohort study. Journal of the American College of Cardiology Conference: 68th Annual Scientific Session of the American College of Cardiology: ACC 19 United States 2019;73 (9 Supplement 1):338.
(629) Pokushalov E, Romanov A, Katritsis D, Strelnikov A, Artemenko S, Bayramova S, Losik D, Baranova V, Karaskov A, Steinberg J. Relationship between reduction of systolic blood pressure and atrial fibrillation burden after renal denervation and pulmonary vein isolation. Journal of the American College of Cardiology Conference: 65th Annual Scientific Session of the American College of Cardiology and i2 Summit: Innovation in Intervention, ACC 16 Chicago, IL United States Conference Publication 2016;67 (13 SUPPL.):05.
(630) Abbott KC, Reynolds JC, Taylor AJ, Agodoa LY. Hospitalized atrial fibrillation after renal transplantation in the United States. American Journal of Transplantation 2003;3(4):471-6.
(631) Kendzerska T, Gershon AS, Atzema C, Dorian P, Mangat I, Hawker G, Leung RS. Sleep Apnea Increases the Risk of New Hospitalized Atrial Fibrillation: A Historical Cohort Study. Chest 2018 Dec;154(6):1330-9.
(632) Khalfallah M, Elsheikh A. Incidence, predictors, and outcomes of new-onset atrial fibrillation in patients with ST-elevation myocardial infarction. Ann Noninvasive Electrocardiol 2020 Jul;25(4):e12746.
(633) Jorgensen HS, Nakayama H, Reith J, Raaschou HO, Olsen TS. Acute stroke with atrial fibrillation: The Copenhagen Stroke Study. Stroke 1996;27(10):1765-9.
(634) Haft JI, Teichholz LE. Echocardiographic and Clinical Risk Factors for Atrial Fibrillation in Hypertensive Patients With Ischemic Stroke. American Journal of Cardiology 2008;102(10):1348-51.
(635) Fauchier L, Clementy N, Pelade C, Collignon C, Nicolle E, Lip GY. Patients With Ischemic Stroke and Incident Atrial Fibrillation: A Nationwide Cohort Study. Stroke 2015 Sep;46(9):2432-7.
(636) Baturova MA, Lindgren A, Carlson J, Shubik YV, Olsson SB, Platonov PG. Predictors of new onset atrial fibrillation during 10-year follow-up after first-ever ischemic stroke. Int J Cardiol 2015 Nov 15;199:248-52.
(637) Favilla CG, Ingala E, Jara J, Fessler E, Cucchiara B, Messe SR, Mullen MT, Prasad A, Siegler J, Hutchinson MD, Kasner SE. Predictors of Finding Occult Atrial Fibrillation after Cryptogenic Stroke. Stroke 2015;46(5):1210-5.
(638) Bisson A, Bodin A, Clementy N, Babuty D, Lip GYH, Fauchier L. Prediction of Incident Atrial Fibrillation According to Gender in Patients With Ischemic Stroke From a Nationwide Cohort. Am J Cardiol 2018 Feb 15;121(4):437-44.

(639) Li YG, Bisson A, Bodin A, Herbert J, Grammatico-Guillon L, Joung B, Wang YT, Lip GYH, Fauchier L. C2 HEST Score and Prediction of Incident Atrial Fibrillation in Poststroke Patients: A French Nationwide Study. J Am Heart Assoc 2019 Jul 2;8(13):e012546.
(640) Ntaios G, Perlepe K, Lambrou D, Sirimarco G, Strambo D, Eskandari A, Karagkiozi E, Vemmou A, Koroboki E, Manios E, Makaritsis K, Vemmos K, et al. External Performance of the HAVOC Score for the Prediction of New Incident Atrial Fibrillation. Stroke 2020 Dec 12;51(2):457-61.
(641) Chen YL, Wang HT, Chen HC, Liu WH, Hsueh S, Chung WJ, Wu PJ, Liu CH, Chung CM, Lin YS. A risk stratification scoring system for new-onset atrial fibrillation after ischemic stroke: A National cohort study. Medicine (Baltimore) 2020 Jul 2;99(27):e20881.
(642) Jawad-Ul-Qamar M, Chua W, Purmah Y, Nawaz M, Varma C, Davis R, Maher A, Fabritz L, Kirchhof P. Detection of unknown atrial fibrillation by prolonged ECG monitoring in an all-comer patient cohort and association with clinical and Holter variables. Open Heart 2020 May;7(1):e001151.
(643) Arsava EM, Bas DF, Atalar E, Has AC, Oguz KK, Topcuoglu MA. Ischemic stroke phenotype in patients with nonsustained atrial fibrillation. Stroke 2015;46(3):634-40.
(644) Heywood EG, Drake TM, Bradburn M, Lee J, Wilson MJ, Lee MJ. Atrial Fibrillation After Gastrointestinal Surgery: Incidence and Associated Risk Factors. J Surg Res 2019 Jun;238:23-8.
(645) Arakawa M, Miyata H, Uchida N, Motomura N, Katayama A, Tamura K, Sueda T, Takamoto S. Postoperative atrial fibrillation after thoracic aortic surgery. Annals of Thoracic Surgery 2015;99(1):103-8.
(646) Verdecchia P, Dagenais G, Healey J, Gao P, Dans AL, Chazova I, Binbrek AS, Iacobellis G, Ferreira R, Holwerda N, Karatzas N, Keltai M, et al. Blood pressure and other determinants of new-onset atrial fibrillation in patients at high cardiovascular risk in the Ongoing Telmisartan Alone and in Combination With Ramipril Global Endpoint Trial/Telmisartan Randomized AssessmeNt Study in ACE iNtolerant subjects with cardiovascular Disease studies. J Hypertens 2012 May;30(5):1004-14.
(647) Nurozler F, Tokgozoglu L, Pasaoglu I, Boke E, Ersoy U, Bozer AY. Atrial fibrillation after coronary artery bypass surgery: Predictors and the role of MgSO4 replacement. Journal of Cardiac Surgery 1996;11(6):421-7.
(648) Adabag S, Mithani S, Al AB, Collins D, Bertog S, Bloomfield HE. First clinical evidence on the effects of gemfibrozil in preventing atrial fibrillation: Data from a large randomized controlled trial. Heart Rhythm Conference: 30th Annual Scientific Sessions of the Heart Rhythm Society, Heart Rhythm 2009;May.
(649) Vyssoulis G, Karpanou E, Adamopoulos D, Kyvelou S-M, Tzamou V, Pietri P, Spanos P, Stefanadis C. Metabolic syndrome and atrial fibrillation in patients with essential hypertension. Journal of Clinical Hypertension Conference: 25th Annual Scientific Meeting and Exposition of the American Society of Hypertension, Inc New York, NY United States Conference Publication 2010;12 (SUPPL.1):A13.
(650) Cabrera GS, Valles E, Benito B, Ramos P, Bruguera J, Marti-Almor J. Twenty-four hours holter monitoring and clinical predictors for new onset atrial fibrillation in a non-selected population. European Heart Journal Conference: European Society of Cardiology, ESC Congress 2015;01.
(651) Tsiachris D, Tsioufis C, Antonakis V, Dimitriadis K, Flessas D, Andrikou E, Aragiannis D, Kefala A, Stefanadis C, Tousoulis D. Predictive role of hypertension related organ damage and blood pressure control patterns for the incidence of new-onset atrial fibrillation in essential hypertensives. European Heart Journal Conference: European Society of Cardiology, ESC Congress 2015;01.
(652) Gupta A, Whiteley W, Godec T, Rostamian S, Whitehouse A, MacKay J, Sever P. Long term benefits of blood pressure treatment on the incidence of atrial fibrillation, heart failure and cardiovascular morbidity and mortality: 20-years follow-up of ascot-legacy. Journal of

Hypertension Conference: European Meeting on Hypertension and Cardiovascular Protection and International Society of Hypertension, ESH-ISH 2021;(SUPPL 1):April.
(653) Tsagalis G, Bakirtzi N, Psimenou E, Manios E, Chouliaras I, Koutroubas G, Makris F, Stamellou E, Pappagiannidou P, Akrivos T, Vemmos K. Atrial fibrillation in chronic hemodialysis patients: Prevalence, types, characteristics and treatment practices in Greece. NDT Plus Conference: 17th ERA-EDTA Congress - II DGfN Congress Munich Germany Conference Publication 2010;3 (SUPPL.3):192.
(654) Nakazawa Y, Ashihara T, Tsutamoto T, Ito M, Horie M. Endothelin-1 as a predictor of atrial fibrillation recurrence after pulmonary vein isolation. Heart Rhythm 2009;6(6):725-30.
(655) Pawlowska-Jenerowicz W, Dabrowski M. The metabolic syndrome as a risk factor for idiopathic paroxysmal atrial fibrillation: A one year follow-up after a first episode of atrial fibrillation. European Heart Journal Conference: European Society of Cardiology, ESC Congress 2009;September.
(656) Mason PK, Lake DE, Xiao Y, DiMarco JP, Ferguson JD, Mahapatra S, Mangrum JM, Moorman LP, Moorman JR. Gender differences in the clinical history and treatment of patients referred to an atrial fibrillation center. Heart Rhythm Conference: 31st Annual Scientific Sessions of the Heart Rhythm Society, Heart Rhythm 2010;May.
(657) Schutgens REG, Klamroth R, Pabinger I, Malerba M, Dolan G. Atrial fibrillation in patients with haemophilia: A cross-sectional evaluation in Europe. Haemophilia 2014;20(5):682-6.
(658) Krylova NS, Demkina AE, Khashieva FM, Kovalevskaya EA, Poteshkina NG. Atrial fibrillation and flutter in hypertrophic cardiomyopathy. [Russian]. Russian Journal of Cardiology 2015;121(5):64-70.
(659) Naydenov S, Runev N, Manov E, Vasileva D, Rangelov Y, Naydenova N. Risk Factors, Co-Morbidities and Treatment of In-Hospital Patients with Atrial Fibrillation in Bulgaria. Medicina (Kaunas) 2018 May 25;54(3):34.
(660) Bahloul A, Bouattour N, Triki F, Hammami R, Charfeddine S, Ellouze T, Mhiri C, Abid L, Kammoun S. Observational study of the prevalence of non-valvular atrial fibrillation and ischemic stroke and the factors associated with non-valvular atrial fibrillation in hypertensive patients: About 2887 cases. [Spanish]. Pan African Medical Journal 38(31) (pp 1-11), 2021;13.
(661) Fabbian F, Catalano C, Lambertini D, Tarroni G, Bordin V, Squerzanti R, Gilli P, Di LD, Cavagna R. Clinical characteristics associated to atrial fibrillation in chronic hemodialysis patients. Clinical Nephrology 2000;54(3):234-9.
(662) Planas F, Antunez F, Poblet T, Pujol M, Romero C, Sadurni J, Vazquez G, Moya A, Navarro LF. Paroxsysmal lone atrial fibrillation. Clinical profile (PAF registry). Revista espanola de cardiologia 2001;54(7):838-44.
(663) Bertomeu M, V, Morillas Blasco PJ, Gonzalez Juanatey JR, Alegria EE, Garcia Acuna JM, Gonzalez M, I, Frutos GA, Valero PR, Rodriguez Ortega JA. Antithrombotic treatment in hypertensive patients with chronic atrial fibrillation. CARDIOTENS 99 study. Medicina Clinica 2002;118(9):327-31.
(664) Greene HL. Baseline characteristics of patients with atrial fibrillation: The AFFIRM study. American Heart Journal 2002;143(6):991-1001.
(665) Genovesi S, Pogliani D, Faini A, Valsecchi MG, Riva A, Stefani F, Acquistapace I, Stella A, Bonforte G, DeVecchi A, DeCristofaro V, Buccianti G, et al. Prevalence of atrial fibrillation and associated factors in a population of long-term hemodialysis patients. American Journal of Kidney Diseases 2005;46(5):897-902.
(666) Cameron AC, Jenkins SMM, Dunn FG. The burden of atrial fibrillation in unselected acute medical admissions. Scottish Medical Journal 2008;53(2):42-61.
(667) Benedetto U, Melina G, Roscitano A, Ciavarella GM, Tonelli E, Sinatra R. Clinical Utility of Tissue Doppler Imaging in Prediction of Atrial Fibrillation After Coronary Artery Bypass Grafting. Annals of Thoracic Surgery 2007;83(1):83-8.

(668) Chang WL, Hou CJY, Wei SP, Tsai JP, Chen YJ, Chen ML, Chuech CC, Hung CL, Huang MY, Lee CH, Yeh HI, Shih SC. Utilization and Clinical Feasibility of a Handheld Remote Electrocardiography Recording Device in Cardiac Arrhythmias and Atrial Fibrillation: A Pilot Study. International Journal of Gerontology 2015;9(4).
(669) Aker A, Saliba W, Hislop E, Zafrir B. Blood pressure measurements during treadmill exercise testing and the risk for the future development of atrial fibrillation. Hypertens Res 2022 Apr 20.
(670) Kannel WB. Potency of vascular risk factors as the basis for antihypertensive therapy. European Heart Journal 1992;13 (SUPPL.G):34-42.
(671) Kalman JM, Tonkin AM. Atrial fibrillation: Epidemiology and the risk and prevention of stroke. PACE - Pacing and Clinical Electrophysiology 1992;15(9):1332-46.
(672) Kannel WB. Hypertension as a risk factor for cardiac events - Epidemiologic results of long-term studies. Journal of Cardiovascular Pharmacology 1993;21 (SUPPL.2):S27-S37.
(673) Levy S. Epidemiology and nosology of atrial fibrillation. [French]. Archives des maladies du coeur et des vaisseaux 1994;87 Spec No 3:11-5.
(674) Perinovic R, Todorovic G, Peric M, Tomic M. Atrial fibrillation in general medicine: Prevalence, etiology and therapeutic options. [Serbian]. Pharmaca 1994;32(1):33-41.
(675) Domanski MJ. The epidemiology of atrial fibrillation. Coronary Artery Disease 1995;6(2):95-100.
(676) Ukani ZA, Ezekowitz MD. Contemporary management of atrial fibrillation. Medical Clinics of North America 1995;79(5):1135-52.
(677) Berjon RJ, Olaz PF, de los Arcos LE. [Epidemiology, risk factors, and pathogeny of atrial fibrillation and atrial flutter]. Rev Esp Cardiol 1996;49 Suppl 2:1-7.
(678) Chandramouli BV, Kotler MN. Atrial fibrillation: Drug therapies for ventricular rate control and restoration of sinus rhythm. Geriatrics 1998;53(6):46-60.
(679) Aronow WS. Prevalence of heart disease in older women in a nursing home. Journal of Women's Health 1998;7(9):1105-12.
(680) Levy S. Epidemiology and classification of atrial fibrillation. Journal of Cardiovascular Electrophysiology 1998;9 (8 SUPPL.):S78-S82.
(681) Ryder KM, Benjamin EJ. Epidemiology and significance of atrial fibrillation. American Journal of Cardiology 1999;84 (9 SUPPL.1):131-8.
(682) Bhatia A, Sra J. Atrial fibrillation: epidemiology, mechanisms and management. Indian Heart J 2000 Mar;52(2):129-64.
(683) Sra J, Dhala A, Blanck Z, Deshpande S, Cooley R, Akhtar M, O'Rourke RA. Atrial fibrillation: Epidemiology, mechanisms, and management. Current Problems in Cardiology 2000;25(7):409-524.
(684) Olshonsky B, Guo H, Barakat T, Levy S. Epidemiology of atrial fibrillation. Disease Management and Clinical Outcomes 2000;2(1-2):50-6.
(685) Brown MJ, Haydock S. Pathoaetiology, epidemiology and diagnosis of hypertension. Drugs 2000;59 (SUPPL.2):1-12.
(686) Chugh SS, Blackshear JL, Shen WK, Hammill SC, Gersh BJ. Epidemiology and natural history of atrial fibrillation: clinical implications. J Am Coll Cardiol 2001 Feb;37(2):371-8.
(687) Hennersdorf MG, Strauer BE. Arterial hypertension and cardiac arrhythmias. J Hypertens 2001 Feb;19(2):167-77.
(688) Iuliano L, Micheletta F. Atrial fibrillation. Epidemiology and physiopathology. [Italian]. Haematologica 2001;86 (9 Suppl):1-4.
(689) Falk RH. Atrial fibrillation. New England Journal of Medicine 2001;344(14):1067-78.
(690) Levy S. Atrial fibrillation, the arrhythmia of the elderly, causes and associated conditions. Anadolu Kardiyoloji Dergisi 2002;2(1):55-60.
(691) Chatap G, Giraud K, Vincent J-P. Atrial fibrillation in the elderly: Facts and management. Drugs and Aging 2002;19(11):819-46.
(692) Jung J. [Atrial fibrillation as endpoint in studies of arterial hypertension]. Dtsch Med Wochenschr 2003 Nov 21;128(47):2493-6.

(693) Healey JS, Connolly SJ. Atrial fibrillation: hypertension as a causative agent, risk factor for complications, and potential therapeutic target. Am J Cardiol 2003 May 22;91(10A):9G-14G.

(694) Augoustides JG, Demers EA. Atrial fibrillation after cardiothoracic surgery: Incidence, risk factors, mechanisms, prevention and treatment. Progress in Anesthesiology 2003;17(16):247-58.

(695) Gersh BJ, Tsang TS, Seward JB. The changing epidemiology and natural history of nonvalvular atrial fibrillation: clinical implications. Transactions of the American Clinical and Climatological Association 2004;115:149-59.

(696) Gallagher D. Current management of atrial fibrillation. Medicine Today 2004;5(2):41-51.

(697) Crystal E, Connolly SJ. Atrial fibrillation: Guiding lessons from epidemiology. Cardiology Clinics 2004;22(1):1-8.

(698) Gersh BJ, Tsang TSM, Barnes ME, Seward JB. The changing epidemiology of non-valvular atrial fibrillation: The role of novel risk factors. European Heart Journal 2005;Supplement. 7 (C):C5-C11.

(699) Le HJY, Messali A, Gorka H, Taamallah K, Cohen I, Lavergne T, Pornin M. Atrial fibrillation and heart failure. [French]. Medecine Therapeutique - Cardio 2005;1(6):553-8.

(700) Hilleman DE, Hunter CB, Mohiuddin SM, Maciejewski S. Pharmacological management of atrial fibrillation following cardiac surgery. American Journal of Cardiovascular Drugs 2005;5(6):361-9.

(701) Tsang TSM, Miyasaka Y, Barnes ME, Gersh BJ. Epidemiological profile of atrial fibrillation: A contemporary perspective. Progress in Cardiovascular Diseases 2005;48(1):1-8.

(702) Pangallo A, Cutrona V. Atrial fibrillation: Epidemiology and clinical impact in Europe. Mediterranean Journal of Pacing and Electrophysiology 2005;7(2):71-3.

(703) Valderrama AL, Dunbar SB, Mensah GA. Atrial fibrillation: Public health implications. American Journal of Preventive Medicine 2005;29 (5 SUPPL.1):75-80.

(704) Healey JS, Morillo CA, Connolly SJ. Role of the renin-angiotensin-aldosterone system in atrial fibrillation and cardiac remodeling. Current Opinion in Cardiology 2005;20(1):31-7.

(705) Andrews M, Nelson BP. Atrial fibrillation. Mount Sinai Journal of Medicine 2006;73(1):482-92.

(706) Birnie DH, Gollob M, Healey JS. Clinical trials, the renin angiotensin system and atrial fibrillation. Current Opinion in Cardiology 2006;21(4):368-75.

(707) Roldan SC, Pablo TJ, Garcia Donaire JA, Segura De La MJ. Importance of the prevention of atrial fibrillation in the hypertensive patient. [Spanish]. Hipertension 2007;24(4):164-71.

(708) Aksnes TA, Flaa A, Strand A, Kjeldsen SE. Prevention of new-onset atrial fibrillation and its predictors with angiotensin II-receptor blockers in the treatment of hypertension and heart failure. Journal of Hypertension 2007;25(1):15-23.

(709) Leibovitch ER. Hypertension 2008, refining our treatment. Geriatrics 2008;63(10):14-20.

(710) Iravanian S, Dudley J. The renin-angiotensin-aldosterone system (RAAS) and cardiac arrhythmias. Heart Rhythm 2008;5 (6 SUPPL.):S12-S17.

(711) Breithardt G, Dobrev D, Doll N, Goette A, Hoffmann B, Kirchhof P, Koster I, Kuck K-H, Leute A, Meinertz T, Nabauer M, Oeff M, et al. The German Competence Network on Atrial Fibrillation (AFNET). Herz 2008;33(8):548-55.

(712) Aksnes TA, Flaa A, Strand A, Kjeldsen SE. Prevention of atrial fibrillation in hypertension. Current Hypertension Reports 2008;10(3):175-81.

(713) Nguyen JT, Benditt DG. Atrial fibrillation susceptibility in metabolic syndrome: Simply the sum of its parts? Circulation 2008;117(10):1249-51.

(714) Savelieva I, Camm J. Update on atrial fibrillation: Part I. Clinical Cardiology 2008;31(2):55-62.

(715) Schoonderwoerd BA, Smit MD, Pen L, Van G, I. New risk factors for atrial fibrillation: Causes of 'not-so-lone atrial fibrillation'. Europace 2008;10(6):668-73.

(716) Kannel WB, Benjamin EJ. Status of the Epidemiology of Atrial Fibrillation. Medical Clinics of North America 2008;92(1):17-40.
(717) Lakshminarayan K, Anderson DC, Herzog CA, Qureshi AI. Clinical epidemiology of atrial fibrillation and related cerebrovascular events in the United States. Neurologist 2008;14(3):143-50.
(718) Go O, Rosendorff C. Hypertension and atrial fibrillation. Curr Cardiol Rep 2009 Nov;11(6):430-5.
(719) Conen D, Osswald S, Albert CM. Epidemiology of atrial fibrillation. Swiss Med Wkly 2009 Jun 27;139(25-26):346-52.
(720) Solun B, Marcoviciu D, Dicker D. Does treatment of hypertension decrease the incidence of atrial fibrillation and cardioembolic stroke? Eur J Intern Med 2009 Mar;20(2):125-31.
(721) Aksnes TA, Kjeldsen SE, Schmieder RE. Hypertension and atrial fibrillation with emphasis on prevention. Blood Pressure 2009;18(3):94-8.
(722) Voroneanu L, Covic A. Arrhythmias in hemodialysis patients. Journal of Nephrology 2009;22(6):716-25.
(723) Ferro CRC, De O, Nunes FP, Piegas LS. Postoperative atrial fibrillation after cardiac surgery. [Portuguese, Spanish, English]. Arquivos Brasileiros de Cardiologia 93 (1) (pp 59-61), 2009;93(1):59-61.
(724) Naccarelli GV, Peacock F. Angiotensin II receptor blockers in the prevention of complications from atrial fibrillation. Vascular Health and Risk Management 2009;5:783-91.
(725) Sanoski CA. Clinical, economic, and quality of life impact of atrial fibrillation. Journal of Managed Care Pharmacy 2009;15 (6 SUPPL.B):S4-S9.
(726) Barra S, Silvestri N, Vitagliano G, Madrid A, Gaeta G. Angiotensin II receptor blockers in the prevention of atrial fibrillation. Expert Opinion on Pharmacotherapy 2009;10(9):1395-411.
(727) Ma C-S, Du X, Jiang C-X. Atrial fibrillation in China: A brief review. Chinese Medical Journal 2009;122(23):2803-6.
(728) Kannel WB, Benjamin EJ. Current Perceptions of the Epidemiology of Atrial Fibrillation. Cardiology Clinics 2009;27(1):13-24.
(729) Rosendorff C, Go O. Hypertension and atrial fibrillation. Current Cardiology Reports 2009;11(6):430-5.
(730) Moro C, Hernandez-Madrid A, Matia R. Non-antiarrhythmic drugs to prevent atrial fibrillation. American Journal of Cardiovascular Drugs 2010;10(3):165-73.
(731) Levine MJ, Schweitzer P. Angiotensin converting enzyme inhibitors and angiotensin II receptor blockers in atrial fibrillation. Vnitrni Lekarstvi 2010;56(11):1138-41.
(732) Aksnes TA, Kjeldsen SE. A link between hypertension and atrial fibrillation: Methods of treatment and prevention. Current Vascular Pharmacology 2010;8(6):769-74.
(733) McRobbie D. Atrial fibrillation: Clinical features and classification. Clinical Pharmacist 2010;2(11):355-7.
(734) Kozlowski D, Budrejko S, Lip GYH, Rysz J, Mikhailidis DP, Raczak G, Banach M. Lone atrial fibrillation: What do we know? Heart 2010;96(7):498-503.
(735) Kuhne M, Conen D. Atrial fibrillation in women. Therapy 2010;7(2):139-46.
(736) Webb AJS, Rothwell PM. Blood pressure variability and risk of new-onset atrial fibrillation: A systematic review of randomized trials of antihypertensive drugs. Stroke 2010;41(9):2091-3.
(737) Gbadebo TD, Okafor H, Darbar D. Differential impact of race and risk factors on incidence of atrial fibrillation. Am Heart J 2011 Jul;162(1):31-7.
(738) Kumar P, Gehi AK. Atrial Fibrillation and Metabolic Syndrome: Understanding the Connection. J Atr Fibrillation 2012 Oct;5(3):647.
(739) Schnabel RB. Can we predict the occurrence of atrial fibrillation? Clin Cardiol 2012 Jan;35 Suppl 1:5-9.

(740) Lau Y-F, Yiu K-H, Siu C-W, Tse H-F. Hypertension and atrial fibrillation: Epidemiology, pathophysiology and therapeutic implications. Journal of Human Hypertension 2012;26(10):563-9.
(741) Menezes AR, Lavie CJ, DiNicolantonio JJ, O'Keefe J, Morin DP, Khatib S, bi-Samra FM, Messerli FH, Milani RV. Cardiometabolic risk factors and atrial fibrillation. Rev Cardiovasc Med 2013;14(2-4):e73-e81.
(742) Ghiadoni L, Taddei S, Virdis A. Hypertension and Atrial Fibrillation: Any Change with the New Anticoagulants. Curr Pharm Des 2014;20(39):6096-105.
(743) Tadic M, Ivanovic B, Cuspidi C. What do we actually know about the relationship between arterial hypertension and atrial fibrillation? Blood Press 2014 Apr;23(2):81-8.
(744) Lau YC, Lip GYH. Atrial fibrillation. Medicine (United Kingdom) 2014;42(10):598-603.
(745) Nair GM, Nery PB, Redpath CJ, Birnie DH. The role of renin angiotensin system in atrial fibrillation. Journal of Atrial Fibrillation 2014;6(6):91-8.
(746) Rahman F, Kwan GF, Benjamin EJ. Global epidemiology of atrial fibrillation. Nature Reviews Cardiology 2014;11(11):639-54.
(747) Ogunsua AA, Shaikh AY, Ahmed M, McManus DD. Atrial Fibrillation and Hypertension: Mechanistic, Epidemiologic, and Treatment Parallels. Methodist Debakey Cardiovasc J 2015 Oct;11(4):228-34.
(748) Poli D, Antonucci E. Epidemiology, diagnosis, and management of atrial fibrillation in women. Int J Womens Health 2015;7:605-14.
(749) Kokubo Y, Iwashima Y. Higher blood pressure as a risk factor for diseases other than stroke and ischemic heart disease. Hypertension 2015 Aug;66(2):254-9.
(750) Menezes AR, Lavie CJ, De SA, Milani RV, O'Keefe J, DiNicolantonio JJ, Morin DP, bi-Samra FM. Lifestyle Modification in the Prevention and Treatment of Atrial Fibrillation. Progress in Cardiovascular Diseases 2015;58(2):117-25.
(751) Sheikh A, Patel NJ, Nalluri N, Agnihotri K, Spagnola J, Patel A, Asti D, Kanotra R, Khan H, Savani C, Arora S, Thakkar B, et al. Trends in Hospitalization for Atrial Fibrillation: Epidemiology, Cost, and Implications for the Future. Progress in Cardiovascular Diseases 2015;58(2):105-16.
(752) Tsiachris D, Tsioufis C, Mazzone P, Katsiki N, Stefanadis C. Atrial fibrillation and chronic kidney disease in hypertension: A common and dangerous triad. Current Vascular Pharmacology 2015;13(1):111-20.
(753) Hajhosseiny R, Matthews GK, Lip GYH. Metabolic syndrome, atrial fibrillation, and stroke: Tackling an emerging epidemic. Heart Rhythm 2015;12(11):2332-43.
(754) Panaich SS, Patel N, Agnihotri K, Arora S, Savani C, Patel NJ, Patel SV, Sonani R, Patel A, Lahewala S, Singh V, Thakkar B, et al. A Review of Hypertension Management in Atrial Fibrillation. Curr Hypertens Rev 2016;12(3):196-202.
(755) Seccia TM, Caroccia B, Muiesan ML, Rossi GP. Atrial fibrillation and arterial hypertension: A common duet with dangerous consequences where the renin angiotensin-aldosterone system plays an important role. Int J Cardiol 2016 Mar 1;206:71-6.
(756) Kokubo Y, Matsumoto C. Traditional cardiovascular risk factors for incident atrial fibrillation. Circulation Journal 2016;80(12):2415-22.
(757) Lip GYH, Coca A, Kahan T, Boriani G, Manolis AS, Olsen MH, Oto A, Potpara TS, Steffel J, Marin F, de Oliveira Figueiredo MJ, de SG, et al. Hypertension and cardiac arrhythmias: a consensus document from the European Heart Rhythm Association (EHRA) and ESC Council on Hypertension, endorsed by the Heart Rhythm Society (HRS), Asia-Pacific Heart Rhythm Society (APHRS) and Sociedad Latinoamericana de Estimulacion Cardiaca y Electrofisiologia (SOLEACE). Europace 2017 Jun 1;19(6):891-911.
(758) Lau DH, Nattel S, Kalman JM, Sanders P. Modifiable Risk Factors and Atrial Fibrillation. Circulation 2017 Aug 8;136(6):583-96.
(759) Wong CX, Brown A, Tse HF, Albert CM, Kalman JM, Marwick TH, Lau DH, Sanders P. Epidemiology of Atrial Fibrillation: The Australian and Asia-Pacific Perspective. Heart Lung Circ 2017 Sep;26(9):870-9.

(760) Lip GYH, Coca A, Kahan T, Boriani G, Manolis AS, Olsen MH, Oto A, Potpara TS, Steffel J, Marin F, de Oliveira Figueiredo MJ, de SG, et al. Hypertension and cardiac arrhythmias: executive summary of a consensus document from the European Heart Rhythm Association (EHRA) and ESC Council on Hypertension, endorsed by the Heart Rhythm Society (HRS), Asia-Pacific Heart Rhythm Society (APHRS), and Sociedad Latinoamericana de Estimulacion Cardiaca y Electrofisiologia (SOLEACE). Eur Heart J Cardiovasc Pharmacother 2017 Oct 1;3(4):235-50.
(761) Staerk L, Sherer JA, Ko D, Benjamin EJ, Helm RH. Atrial Fibrillation: Epidemiology, Pathophysiology, and Clinical Outcomes. Circ Res 2017 Apr 28;120(9):1501-17.
(762) Du X, Dong J, Ma C. Is Atrial Fibrillation a Preventable Disease? J Am Coll Cardiol 2017 Apr 18;69(15):1968-82.
(763) Dzeshka MS, Shahid F, Shantsila A, Lip GYH. Hypertension and Atrial Fibrillation: An Intimate Association of Epidemiology, Pathophysiology, and Outcomes. Am J Hypertens 2017 Aug 1;30(8):733-55.
(764) Allan V, Honarbakhsh S, Casas JP, Wallace J, Hunter R, Schilling R, Perel P, Morley K, Banerjee A, Hemingway H. Are cardiovascular risk factors also associated with the incidence of atrial fibrillation? A systematic review and field synopsis of 23 factors in 32 population-based cohorts of 20 million participants. Thromb Haemost 2017 May 3;117(5):837-50.
(765) Kokubo Y, Matsumoto C. Hypertension Is a Risk Factor for Several Types of Heart Disease: Review of Prospective Studies. Adv Exp Med Biol 2017;956:419-26.
(766) Gorenek CB, Pelliccia Co-Chair A, Benjamin EJ, Boriani G, Crijns HJ, Fogel RI, Van G, I, Halle M, Kudaiberdieva G, Lane DA, Bjerregaard LT, Lip GY, et al. European Heart Rhythm Association (EHRA)/European Association of Cardiovascular Prevention and Rehabilitation (EACPR) position paper on how to prevent atrial fibrillation endorsed by the Heart Rhythm Society (HRS) and Asia Pacific Heart Rhythm Society (APHRS). Eur J Prev Cardiol 2017 Jan;24(1):4-40.
(767) Nalliah CJ, Sanders P, Kalman JM. The Impact of Diet and Lifestyle on Atrial Fibrillation. Curr Cardiol Rep 2018 Oct 12;20(12):137.
(768) Li Y, Pastori D, Guo Y, Wang Y, Lip GYH. Risk factors for new-onset atrial fibrillation: A focus on Asian populations. Int J Cardiol 2018 Jun 15;261:92-8.
(769) Kallistratos MS, Poulimenos LE, Manolis AJ. Atrial fibrillation and arterial hypertension. Pharmacol Res 2018 Feb;128:322-6.
(770) Verdecchia P, Angeli F, Reboldi G. Hypertension and atrial fibrillation: Doubts and certainties from basic and clinical studies. Circulation Research 2018;122(2):352-68.
(771) Boriani G, Proietti M. Atrial fibrillation prevention: An appraisal of current evidence. Heart 2018;104(11):882-7.
(772) Andreadis EA, Geladari CV. Hypertension and atrial fibrillation: A bench to bedside perspective. Frontiers in Bioscience - Scholar 2018;10(2):276-84.
(773) Seccia TM, Caroccia B, Maiolino G, Cesari M, Rossi GP. Arterial Hypertension, Aldosterone, and Atrial Fibrillation. Curr Hypertens Rep 2019 Nov 18;21(12):94.
(774) Gumprecht J, Domek M, Lip GYH, Shantsila A. Invited review: hypertension and atrial fibrillation: epidemiology, pathophysiology, and implications for management. Journal of Human Hypertension 2019;33(12):824-36.
(775) Noubiap JJ, Nyaga UF. A review of the epidemiology of atrial fibrillation in sub-Saharan Africa. Journal of Cardiovascular Electrophysiology 2019;30(12):3006-16.
(776) Panchal G, Mahmood M, Lip GYH. Revisiting the risks of incident atrial fibrillation: A narrative review. Part 2. Kardiologia Polska 2019;77(5):515-24.
(777) Panchal G, Mahmood M, Lip GYH. Revisiting the risks of incident atrial fibrillation: A narrative review. Part 1. Kardiologia Polska 2019;77(4):430-6.
(778) Fuchs FD, Whelton PK. High Blood Pressure and Cardiovascular Disease. Hypertension 2020 Dec 23;75(2):285-92.

(779) Wingerter R, Steiger N, Burrows A, Estes NAM, III. Impact of Lifestyle Modification on Atrial Fibrillation. Am J Cardiol 2020 Jan 15;125(2):289-97.
(780) Vitolo M, Lip GYH, Shantsila A. Why is atrial fibrillation so frequent in hypertensive patients? Am J Hypertens 2020 Sep 23;doi: 10.1093/ajh/hpaa157.
(781) Chung MK, Eckhardt LL, Chen LY, Ahmed HM, Gopinathannair R, Joglar JA, Noseworthy PA, Pack QR, Sanders P, Trulock KM. Lifestyle and Risk Factor Modification for Reduction of Atrial Fibrillation: A Scientific Statement From the American Heart Association. Circulation 2020 Apr 21;141(16):e750-e772.
(782) Kavousi M. Differences in Epidemiology and Risk Factors for Atrial Fibrillation Between Women and Men. Front Cardiovasc Med 2020;7:3.
(783) Wu Z, Fang J, Wang Y, Chen F. Prevalence, outcomes, and risk factors of new-onset atrial fibrillation in critically ill patients a systematic review. International Heart Journal 2020;61(3):476-85.
(784) Ariyaratnam JP, Middeldorp M, Thomas G, Noubiap JJ, Lau D, Sanders P. Risk Factor Management Before and After Atrial Fibrillation Ablation. Card Electrophysiol Clin 2020 Jun;12(2):141-54.
(785) Pathak RK, Abhilash SP, Hendriks JM. A Team-Based Approach Toward Risk Factors of Atrial Fibrillation. Card Electrophysiol Clin 2021 Mar;13(1):257-62.
(786) Lau DH, Shenasa HA, Shenasa M. Hypertension, Prehypertension, Hypertensive Heart Disease, and Atrial Fibrillation. Card Electrophysiol Clin 2021 Mar;13(1):37-45.
(787) O'Keefe EL, Sturgess JE, O'Keefe JH, Gupta S, Lavie CJ. Prevention and Treatment of Atrial Fibrillation via Risk Factor Modification. Am J Cardiol 2021 Dec 1;160:46-52.
(788) Carna Z, Osmancik P. The effect of obesity, hypertension, diabetes mellitus, alcohol, and sleep apnea on the risk of atrial fibrillation. Physiol Res 2021 Dec 30;70(Suppl4):S511-S525.
(789) Marazzato J, Blasi F, Golino M, Verdecchia P, Angeli F, De PR. Hypertension and Arrhythmias: A Clinical Overview of the Pathophysiology-Driven Management of Cardiac Arrhythmias in Hypertensive Patients. J Cardiovasc Dev Dis 2022 Apr 6;9(4).
(790) Manolis AJ. Hypertension and atrial fibrillation. American Journal of Hypertension Conference: 2nd International Conference on Fixed Combination in the Treatment of Hypertension, Dyslipidemia and Diabetes Mellitus: An Ideal Approach for Improving Compliance and Combating Cardiovascular Disease Valencia 2009;November.
(791) Allan V, Honarbakhsh S, Casas JP, Wallace J, Hunter R, Schilling R, Perel P, Morley K, Banerjee A, Hemingway H. Are cardiovascular risk factors also associated with the incidence of atrial fibrillation? A systematic reviewand field synopsis of 23 factors in 32 initially healthy cohorts of 20 million participants. Europace Conference: Heart Rhythm Congress 2016;(Supplement 2):October.
(792) 2nd International Symposium on Hypertension. Kidney and Blood Pressure Research Conference: 2nd International Symposium on Hypertension Osijek Croatia Conference Publication 2010;33(6).
(793) Guedon-Moreau L, Kouakam C, Kacet S. Epidemiology of paroxysmal atrial fibrillation. [French]. Archives des maladies du coeur et des vaisseaux 1998;91(1):67-71.
(794) Neuss H. Epidemiology of atrial fibrillation. [German]. Herz Kreislauf 1998;30(1):11-8.
(795) Evers S, Hennersdorf MG, Perings C, Strauer BE. The epidemiology and causes of atrial fibrillation. [German]. Herzschrittmachertherapie und Elektrophysiologie 2001;12(2):59-67.
(796) Sack S. Epidemiology of atrial fibrillation. [German]. Herz 2002;27(4):294-300.
(797) Mattle HP, Schwerzmann M, Seiler C. Atrial fibrillation and stroke. [German]. Therapeutische Umschau 2003;60(9):527-34.
(798) Schuchert A, Gerth A, Nabauer M, Steinbeck G, Meinertz T. Epidemiology, clinical characteristics and prognosis of atrial fibrillation. [German]. Medizinische Welt 2005;56(9):361-5.
(799) Sotobata I, Yamauchi K, Tsuzuki M. Arrhythmias in the elderly patients: Atrial fibrillation. [Japanese]. Japanese Journal of Geriatrics 1985;22(4):325-33.

(800) Hueb TO. Atrial fibrillation. [Portuguese]. Revista Brasileira de Medicina 66 (4) (pp 87-91), 2009;66(4):87-91.
(801) Musil V. Risk factors of atrial fibrillation. [Slovak]. Kardiologicka Revue 2010;12(3):131-3.
(802) Lopez GM, Arribas F, Velazquez MT, Casas P. Hypertensive cardiopathy and arrhythmias. [Spanish]. Revista espanola de cardiologia 1997;50 Suppl 4:68-73.
(803) Alonso VB, Von W, Payeras AC. Prevention of atrial fibrillation in hypertensive patients. [Spanish]. Medicina Clinica 2007;128(4):148-54.
(804) Summaries for patients. Effect of antihypertensive drugs on risk for atrial fibrillation. Annals of internal medicine 2010;152(2):1-16.
(805) Li LH, Sheng CS, Hu BC, Huang QF, Zeng WF, Li GL, Liu M, Wei FF, Zhang L, Kang YY, Song J, Wang S, et al. The prevalence, incidence, management and risks of atrial fibrillation in an elderly Chinese population: a prospective study. BMC Cardiovasc Disord 2015 May 8;15:31.
(806) Alonso A, Roetker NS, Soliman EZ, Chen LY, Greenland P, Heckbert SR. Prediction of Atrial Fibrillation in a Racially Diverse Cohort: The Multi-Ethnic Study of Atherosclerosis (MESA). J Am Heart Assoc 2016 Feb 23;5(2).
(807) Casaclang-Verzosa G, Barnes ME, Blume G, Seward JB, Gersh BJ, Cha SS, Bailey KR, Tsang TSM. C-reactive protein, left atrial volume, and atrial fibrillation: A prospective study in high-risk elderly. Echocardiography 2010;27(4):394-9.
(808) Volgman AS, Dunn P, Sundberg A, Conard S, Chakravarty P, Htway Z, Waldo A, Albert C, Turakhia MP, Naccarelli GV. Risk factors for symptomatic atrial fibrillation-analysis of an outpatient database. Journal of Atrial Fibrillation 2019;12(1):2141.
(809) Velasco A, Ayers C, Das SR, de Lemos JA, Vongpatanasin W. Prognostic significance of isolated hypertension during the alerting reaction to predict 10-year risk of cardiovascular events in the Dallas heart study. Circulation Conference: American Heart Association's 2014;25.
(810) Benjamin EJ, Chen P-S, Bild DE, Mascette AM, Albert CM, Alonso A, Calkins H, Connolly SJ, Curtis AB, Darbar D, Ellinor PT, Go AS, et al. Prevention of atrial fibrillation. Report from a national heart, lung, and blood institute workshop. Circulation 2009;119(4):606-18.

Supplementary Table 2. Table of risk estimates and 95% confidence intervals from the nonlinear dose-response analyses of systolic and diastolic blood pressure and risk of atrial fibrillation

Systolic blood pressure		Diastolic blood pressure	
mmHg	RR (95% CI)	mmHg	RR (95% CI)

90	1.00	60	1.00
100	1.11 (1.08-1.13)	70	1.20 (1.15-1.25)
110	1.21 (1.17-1.26)	80	1.38 (1.28-1.49)
120	1.34 (1.27-1.41)	90	1.55 (1.40-1.71)
130	1.47 (1.37-1.56)	100	1.71 (1.52-1.91)
140	1.61 (1.49-1.73)	110	1.86 (1.64-2.11)
150	1.76 (1.62-1.92)	120	2.00 (1.75-2.29)
160	1.93 (1.76-2.12)	130	2.13 (1.85-2.46)
170	2.11 (1.90-2.34)	140	2.25 (1.95-2.61)
180	2.30 (2.06-2.57)	150	2.37 (2.04-2.75)
190	2.51 (2.22-2.83)	160	2.47 (2.13-2.88)
200	2.73 (2.39-3.11)		
$p_{nonlinearity}$	<0.0001	$p_{nonlinearity}$	<0.0001

Supplementary Figure 1. Funnel plot for hypertension and atrial fibrillation

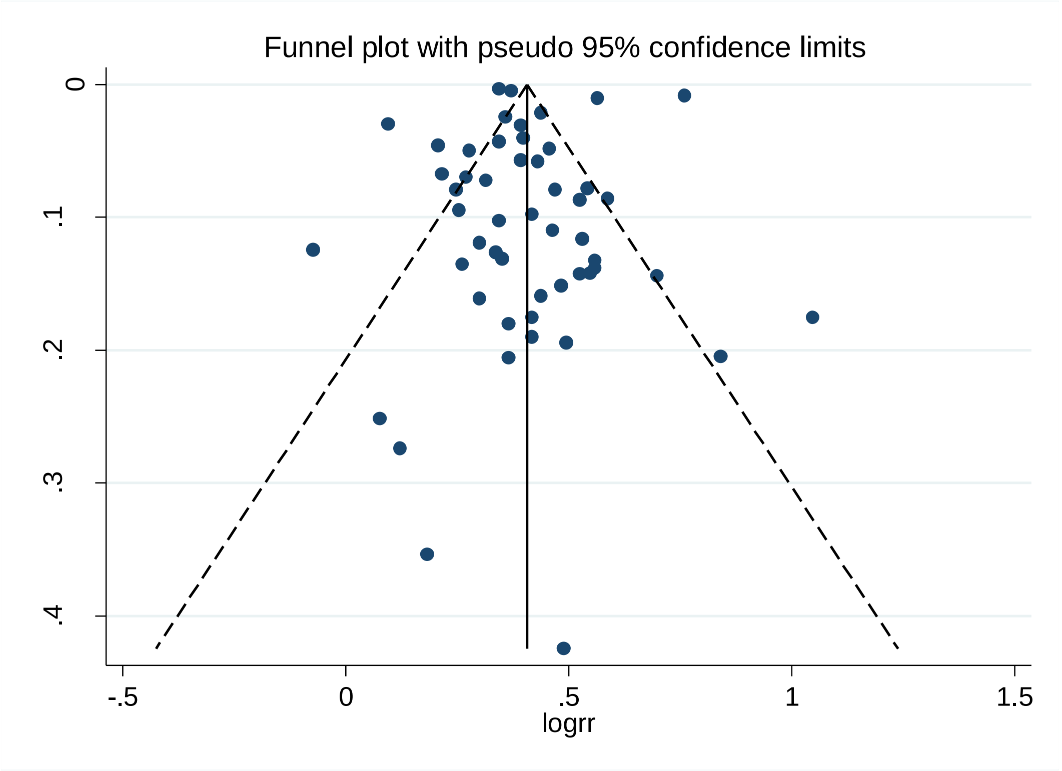

Supplementary Figure 2. Influence analysis of hypertension and atrial fibrillation

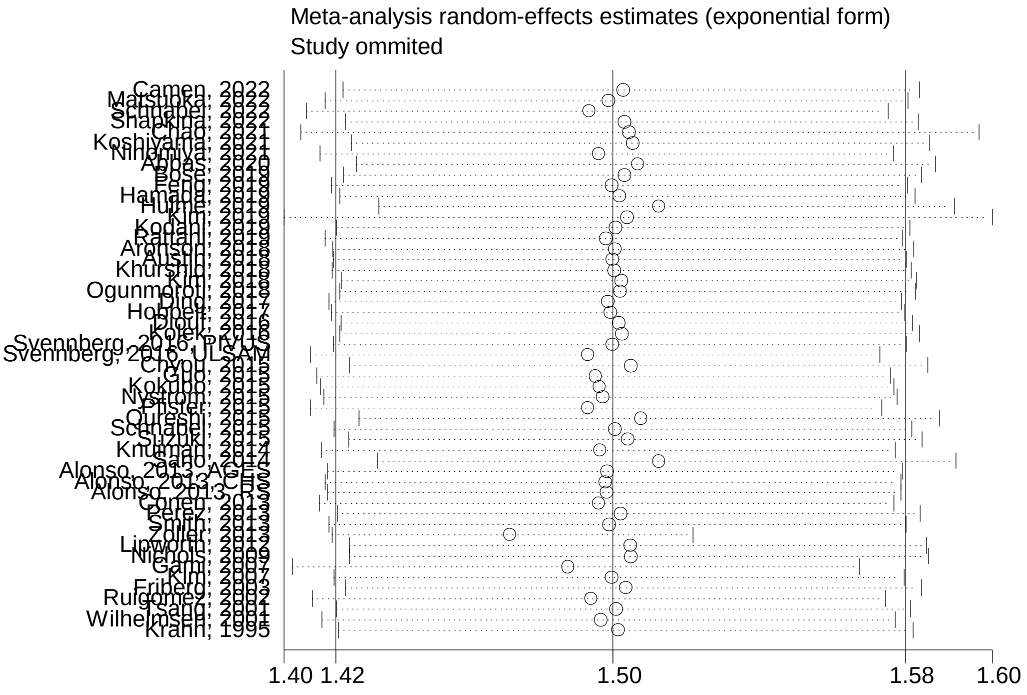

Study omitted	e^coef.	[95% Conf.	Interval]
Camen, 2022	1.4997672	1.4214001	1.5824549
Matsuoka, 2022	1.4955901	1.4162384	1.5793877
Schnabel, 2022	1.4901563	1.4110751	1.5736694
Shapkina, 2022	1.4999818	1.4220499	1.5821846
Chao, 2021	1.5013397	1.4094297	1.5992432
Koshiyama, 2021	1.5023394	1.4236654	1.585361
Ninomiya, 2021	1.4929079	1.4149643	1.5751451
Abbas, 2020	1.5038142	1.4250351	1.5869484
Bose, 2019	1.5000864	1.4214607	1.5830611
Feng, 2019	1.4964237	1.4181178	1.5790536
Hamada, 2019	1.4986777	1.4203949	1.581275
Hulme, 2019	1.5097263	1.4313128	1.5924356
Kim, 2019	1.5006677	1.4049261	1.6029338
Kodani, 2019	1.497528	1.4195752	1.5797613
Rattani, 2019	1.4947912	1.4162502	1.5776877
Aronson, 2018	1.4974464	1.4184625	1.5808284
Austin, 2018	1.4965996	1.4186085	1.5788783
Khurshid, 2018	1.497129	1.4183621	1.5802702
Kim, 2018	1.4991148	1.4209478	1.5815818
Ogunmoroti, 2018	1.4987319	1.4203335	1.5814576
Ding, 2017	1.4953101	1.4174446	1.5774529
Hobbelt, 2017	1.4961448	1.4181224	1.5784597
Diouf, 2016	1.4985143	1.4207449	1.5805407

Kolek, 2016	1.4993396	1.4205122	1.5825412
Svennberg, 2016, PIVUS	1.4966245	1.4186753	1.5788566
Svennberg, 2016, ULSAM	1.4897555	1.4122558	1.5715084
Chyou, 2015	1.5017442	1.4230419	1.5847992
Guo, 2015	1.4920665	1.4139124	1.5745405
Kokubo, 2015	1.4930539	1.4151267	1.5752724
Nystrom, 2015	1.4939882	1.4160229	1.5762461
Pfister, 2015	1.4899063	1.412199	1.5718895
Qureshi, 2015	1.5047543	1.425867	1.5880061
Schnabel, 2015	1.4973549	1.4187465	1.5803187
Suzuki, 2015	1.5009463	1.4229344	1.583235
Knuiman, 2014	1.4933128	1.4152731	1.5756558
Sano, 2014	1.5096912	1.4309905	1.5927203
Alonso, 2013, AGES	1.4951352	1.41696	1.5776232
Alonso, 2013, CHS	1.4946015	1.4162953	1.5772372
Alonso, 2013, RS	1.4951017	1.4171069	1.5773892
Conen, 2013	1.4928628	1.4147041	1.5753396
Perez, 2013	1.4989314	1.4196699	1.5826182
Smith, 2013	1.4958363	1.4173179	1.5787045
Zoller, 2013	1.4679326	1.4183581	1.5192399
Lipworth, 2012	1.5016656	1.4230964	1.5845727
Nichols, 2009	1.5018666	1.4230348	1.5850652
Gami, 2007	1.4843477	1.4071448	1.5657862
Kim, 2007	1.4964409	1.4188254	1.5783024
Friberg, 2003	1.5004478	1.42212	1.5830895
Ruigomez, 2002	1.4907293	1.4127061	1.5730618
Tsang, 2001	1.4976336	1.4196048	1.5799513
Wilhelmsen, 2001	1.4934211	1.4154878	1.5756452
Krahn, 1995	1.4982611	1.4200191	1.5808141
Combined	1.4968944	1.4194031	1.5786163

Supplementary Figure 3. Funnel plot for systolic blood pressure and atrial fibrillation

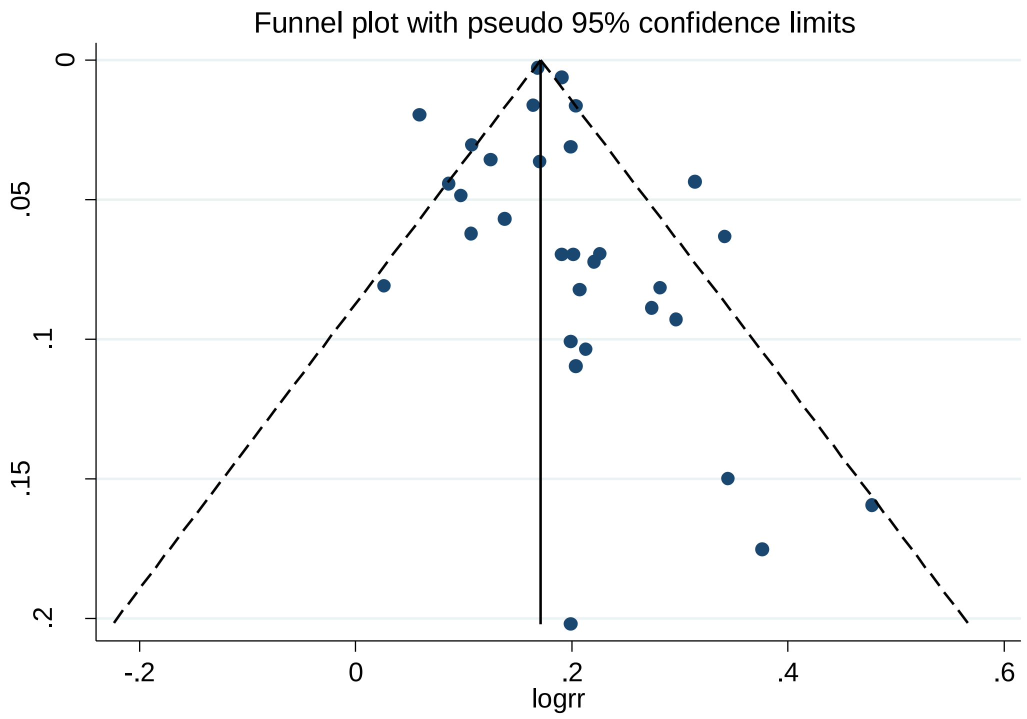

Supplementary Figure 4. Trim and fill plot for systolic blood pressure and atrial fibrillation

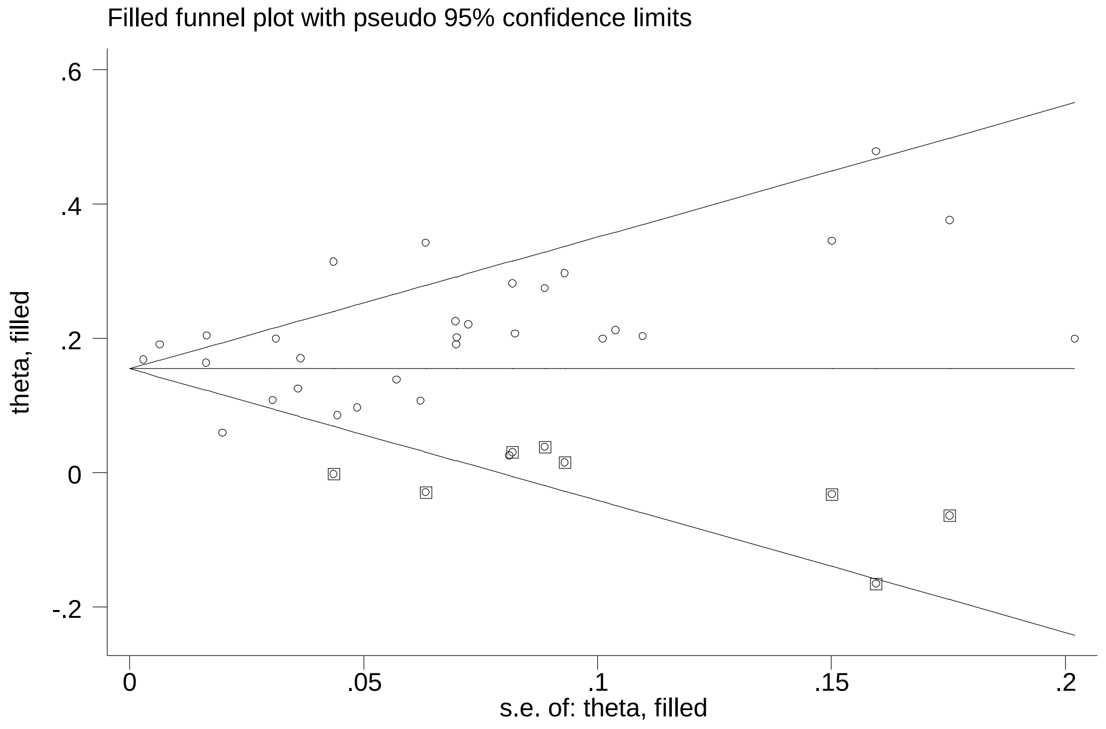

Supplementary Figure 5. Influence analysis of systolic blood pressure and atrial fibrillation

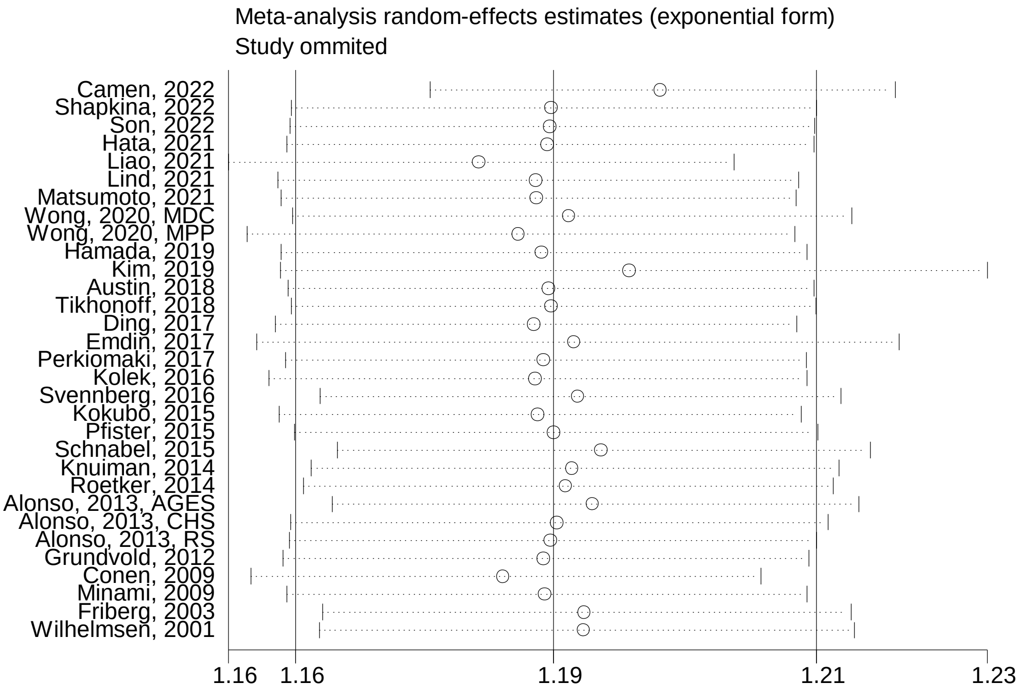

Study omitted	e^coef.	[95% Conf.	Interval]
Camen, 2022	1.1976388	1.1767863	1.2188607
Shapkina, 2022	1.1877569	1.1642468	1.2117418
Son, 2022	1.1876185	1.1641256	1.2115855
Hata, 2021	1.1874317	1.1638235	1.2115189
Liao, 2021	1.1812114	1.1585784	1.2042863
Lind, 2021	1.1863358	1.1630076	1.2101319
Matsumoto, 2021	1.1863893	1.1633377	1.2098978
Wong, 2020, MDC	1.1893901	1.1643606	1.2149576
Wong, 2020, MPP	1.1847706	1.1602399	1.2098198
Hamada, 2019	1.1868715	1.1633159	1.2109041
Kim, 2019	1.1948248	1.1632785	1.2272266
Austin, 2018	1.1874925	1.1639433	1.2115182
Tikhonoff, 2018	1.1877543	1.1642627	1.2117198
Ding, 2017	1.1861609	1.1628008	1.2099904
Emdin, 2017	1.1898341	1.1611429	1.2192342
Perkiomaki, 2017	1.1870571	1.1637495	1.2108315
Kolek, 2016	1.186323	1.1622369	1.2109084
Svennberg, 2016	1.1901798	1.1668754	1.2139497
Kokubo, 2015	1.1865392	1.1631473	1.2104015
Pfister, 2015	1.1879704	1.1645403	1.2118719
Schnabel, 2015	1.1922623	1.1684034	1.2166083
Knuiman, 2014	1.1896825	1.1660655	1.2137779
Roetker, 2014	1.1890799	1.165357	1.2132857

Alonso, 2013, AGES|	1.1915271	1.1679386	1.215592
Alonso, 2013, CHS |	1.1882532	1.1641929	1.2128106
Alonso, 2013, RS |	1.1876715	1.1640469	1.2117757
Grundvold, 2012 |	1.1870556	1.1634976	1.2110906
Conen, 2009 |	1.1834247	1.1605823	1.2067165
Minami, 2009 |	1.1871635	1.1638719	1.210921
Friberg, 2003 |	1.1907495	1.1670963	1.2148823
Wilhelmsen, 2001 |	1.1907203	1.1667671	1.2151653
Combined |	1.187972	1.1646698	1.2117404

Supplementary Figure 6. Funnel plot for diastolic blood pressure and atrial fibrillation

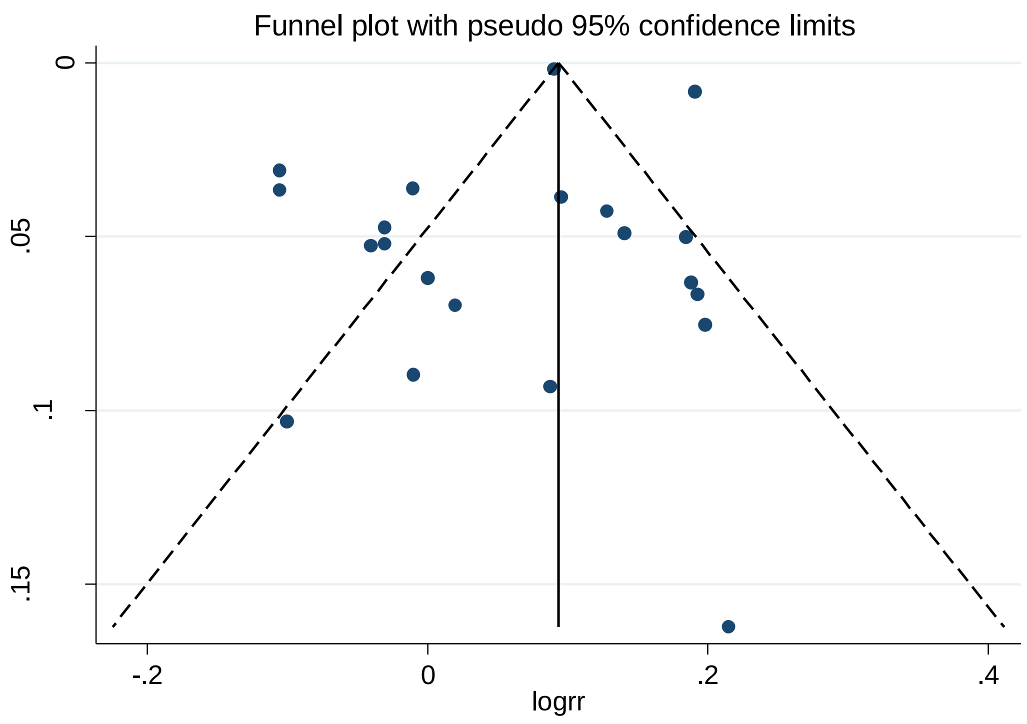

Supplementary Figure 7. Influence analysis of diastolic blood pressure and atrial fibrillation

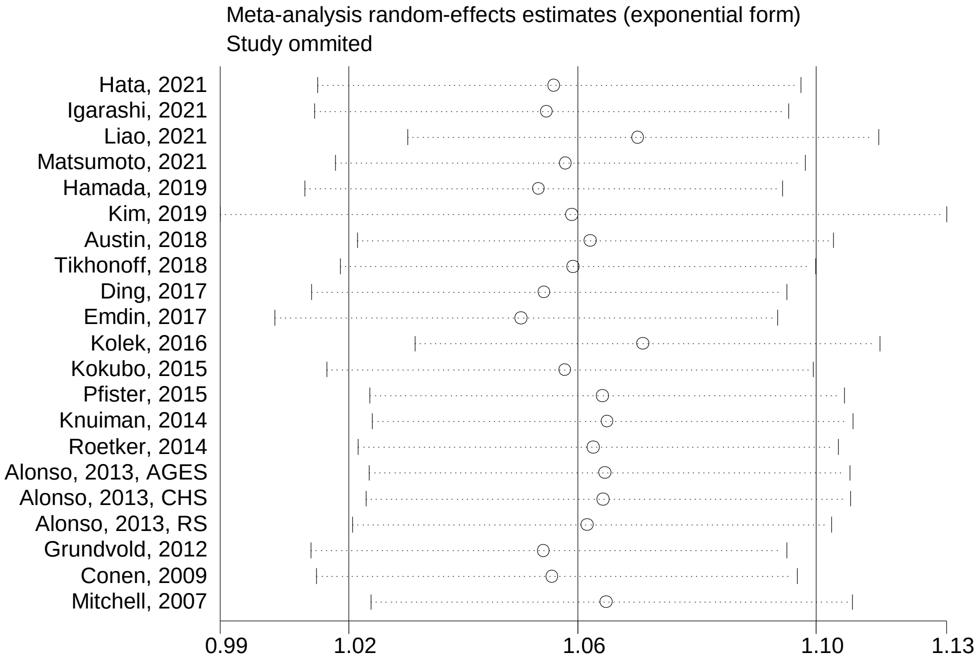

Study omitted	e^coef.	[95% Conf.	Interval]
Hata, 2021	1.0550935	1.0117558	1.1002877
Igarashi, 2021	1.0537671	1.0112333	1.0980898
Liao, 2021	1.0705012	1.0282471	1.1144917
Matsumoto, 2021	1.0572239	1.0150645	1.1011344
Hamada, 2019	1.0522801	1.0094311	1.0969479
Kim, 2019	1.0583782	0.99392426	1.1270119
Austin, 2018	1.0617326	1.0190111	1.106245
Tikhonoff, 2018	1.0585077	1.0158653	1.1029401
Ding, 2017	1.0533018	1.0106745	1.0977271
Emdin, 2017	1.0490129	1.0039659	1.0960813
Kolek, 2016	1.0713086	1.0295725	1.1147367
Kokubo, 2015	1.0570489	1.013441	1.1025332
Pfister, 2015	1.0638981	1.0213573	1.1082107
Knuiman, 2014	1.0648957	1.0218056	1.109803
Roetker, 2014	1.0622835	1.0192127	1.1071745
Alonso, 2013, AGES	1.0643589	1.0212222	1.1093179
Alonso, 2013, CHS	1.0640514	1.0205945	1.1093588
Alonso, 2013, RS	1.0611291	1.018162	1.1059092
Grundvold, 2012	1.0532248	1.0105425	1.0977099
Conen, 2009	1.0546952	1.0115693	1.0996599
Mitchell, 2007	1.0646656	1.0214742	1.1096832
Combined	1.0594561	1.0175355	1.1031037

Supplementary Figure 8: Hypertension and atrial fibrillation, analyses stratified by ethnicity

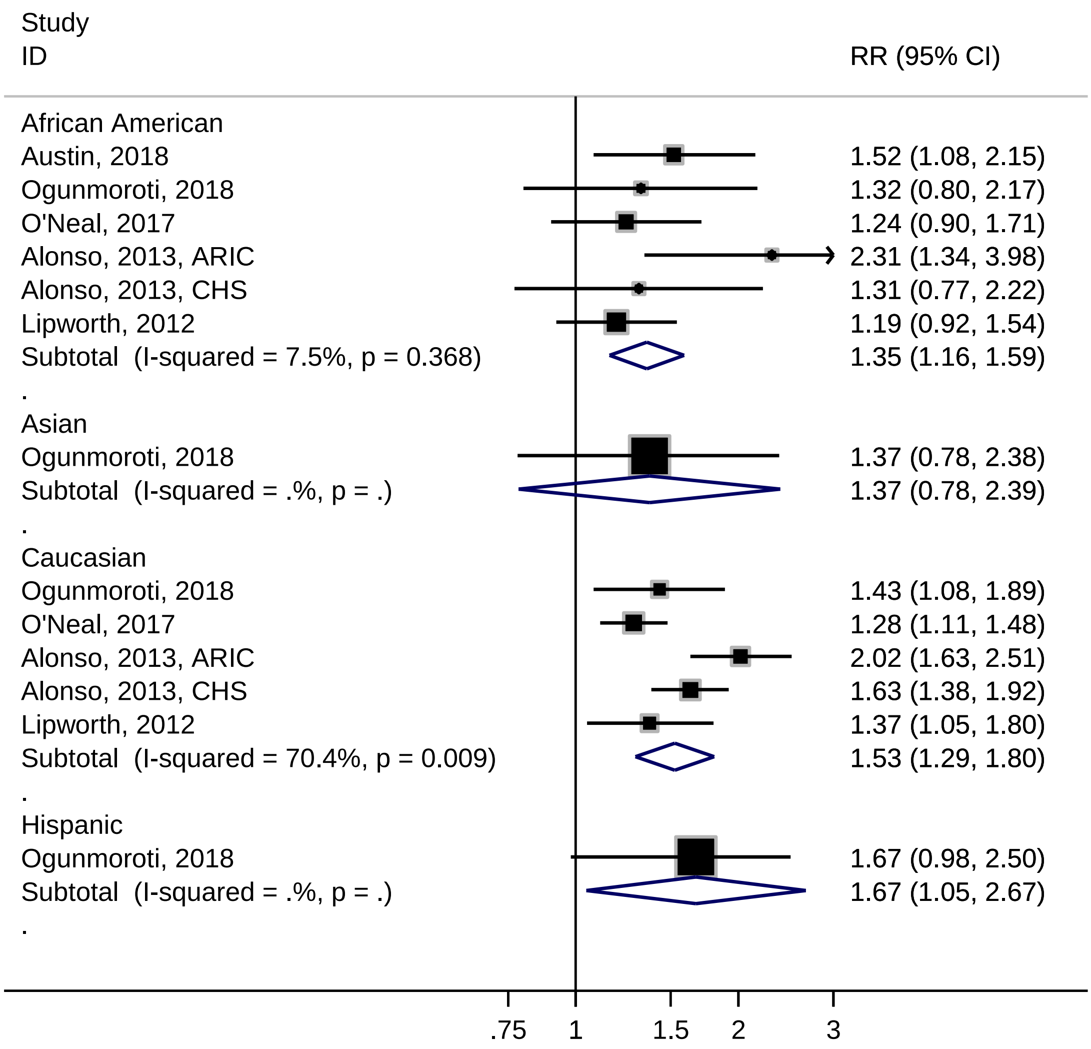
Study
ID
RR (95% CI)
African American
Austin, 2018
1.52 (1.08, 2.15)
Ogunmoroti, 2018
1.32 (0.80, 2.17)
O'Neal, 2017
1.24 (0.90, 1.71)
Alonso, 2013, ARIC
2.31 (1.34, 3.98)
Alonso, 2013, CHS
1.31 (0.77, 2.22)
Lipworth, 2012
1.19 (0.92, 1.54)
Subtotal (I-squared = 7.5%, p = 0.368)
1.35 (1.16, 1.59)
.
Asian
Ogunmoroti, 2018
1.37 (0.78, 2.38)
Subtotal (I-squared = .%, p = .)
1.37 (0.78, 2.39)
.
Caucasian
Ogunmoroti, 2018
1.43 (1.08, 1.89)
O'Neal, 2017
1.28 (1.11, 1.48)
Alonso, 2013, ARIC
2.02 (1.63, 2.51)
Alonso, 2013, CHS
1.63 (1.38, 1.92)
Lipworth, 2012
1.37 (1.05, 1.80)
Subtotal (I-squared = 70.4%, p = 0.009)
1.53 (1.29, 1.80)
.
Hispanic
Ogunmoroti, 2018
1.67 (0.98, 2.50)
Subtotal (I-squared = .%, p = .)
1.67 (1.05, 2.67)
.
.75
1
1.5
2
3
